# Enzyme-Responsive Chemiluminescent Probes Assembled with Iridium(III) Photosensitizers via Host–Guest Chemistry for Chemiluminescence-Induced Photodynamic Therapy

**DOI:** 10.1021/jacs.6c01728

**Published:** 2026-07-01

**Authors:** Jia-Hao Wang, Lawrence Cho-Cheung Lee, Alex Man-Hei Yip, Justin Shum, Qi-Hang Cheng, Eunice Chiu-Lam Mak, Kenneth Kam-Wing Lo

**Affiliations:** † Department of Chemistry, 53025 City University of Hong Kong, Tat Chee Avenue, Hong Kong, P. R. China; ‡ Laboratory for Synthetic Chemistry and Chemical Biology Limited, Units 1503−1511, 15/F, Building 17 W, Hong Kong Science Park, New Territories, Hong Kong, P. R. China; § State Key Laboratory of Terahertz and Millimeter Waves, City University of Hong Kong, Tat Chee Avenue, Hong Kong, P. R. China

## Abstract

Photodynamic therapy (PDT) is effective for localized cancers, but its reliance on external light limits treatment depth and uniformity. Herein, we report a programmable supramolecular chemiluminescence (CL)-induced PDT platform that generates reactive oxygen species (ROS) in the absence of light irradiation and displays intrinsic cancer selectivity. This platform is constructed through the supramolecular interaction between enzyme-responsive chemiluminescent spiroadamantyl phenoxy-1,2-dioxetane probes (**ADO**_**P**_, **ADO**_**E**_, and **ADO**_**G**_) and luminescent iridium­(III) complexes bearing a trimethyl-β-cyclodextrin (TMCD) host [Ir­(N^∧^C)_2_(bpy-TMCD)]­(Cl) (HN^∧^C = 2-phenylpyridine (Hppy) (**1a**), 2-phenylquinoline (Hpq) (**2a**), and 2-(1-naphthyl)­benzothiazole (Hbsn) (**3a**)). The chemiluminescent probes exhibited CL upon reaction with alkaline phosphatase (ALP), porcine liver esterase (PLE), and β-galactosidase (β-gal); while the iridium­(III) complexes showed intense phosphorescence with efficient singlet oxygen production, distinct intracellular localization, and tunable (photo)­cytotoxicity. In aqueous solutions, mixing the chemiluminescent probes with TMCD-tagged iridium­(III) complexes afforded supramolecular adducts that, upon enzyme activation, underwent chemiluminescence resonance energy transfer (CRET) from the dioxetane donors to the iridium­(III) acceptors. Remarkably, the ALP-responsive conjugate formed from probe **ADO**_**P**_ and complex **3a** (adduct **ADO**_**P**_ **–3a**) selectively generated ROS and elicited potent cytotoxicity in cancerous HeLa cells and spheroids under light-free conditions, with apoptosis as the predominant cell death pathway, while remaining noncytotoxic in normal HEK293 cells. This modular platform couples supramolecular host–guest assembly, enzyme-specific activation, and CRET-induced ROS generation to overcome the limitations of conventional PDT, enabling cancer-selective, light-free therapy.

## Graphical abstract

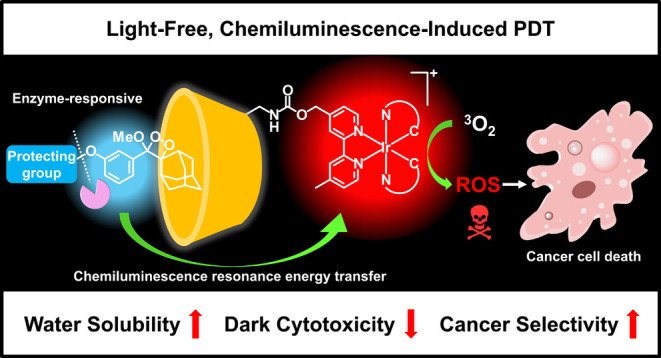

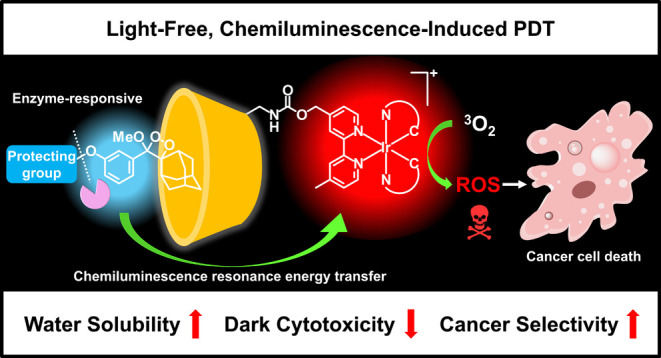

## Introduction

Photodynamic therapy (PDT), as a clinically validated therapeutic modality, has attracted significant attention for its minimal invasiveness and high spatial precision.^1,2^ It utilizes photosensitizers (PSs) to produce reactive oxygen species (ROS) upon photoirradiation. Photofunctional transition metal complexes have played a central role in PS development for PDT due to their remarkable photophysical and photochemical properties, including high photostability, long triplet excited-state lifetimes, and efficient ROS generation.^3–5^ However, these complexes are often associated with challenges such as low aqueous solubility, high dark cytotoxicity, and poor cancer selectivity. To address these issues, recent efforts have focused on enhancing the PDT effectiveness of these complexes through strategies such as modification with polymers,^6–8^ conjugation to peptides or proteins,^9–11^ encapsulation within nanomaterials,^12–14^ and stimuli-responsive PS activation.^15–22^ Despite their efficacy, the limited penetration of light in biological tissues remains a major obstacle, reducing the effectiveness of PDT in treating deep-seated malignancies and solid tumors and thus hindering its wider clinical adoption.^23^

Chemiluminescence (CL)-mediated PDT has emerged as a promising solution by leveraging chemiluminescent agents that emit light upon chemical reactions to excite nearby PSs, thereby eliminating the need for external light excitation.^24^ Common chemiluminogens, including luminol and its derivatives,^25^ peroxyoxalate compounds,^26^ *Cypridina* luciferin analogs,^27^ and phenoxy-1,2-dioxetanes,^28^ have been widely used in biological imaging because they provide superior signal-to-noise ratios.^29–31^ Phenoxy-1,2-dioxetanes are particularly attractive due to their excellent thermal stability conferred by steric hindrance and their tunable protecting groups that allow selective activation by a wide range of biological triggers such as ions,^32–34^ small molecules,^35–39^ and enzymes.^40–47^ Ruthenium­(II) and iridium­(III) complexes have been conjugated to spiroadamantyl phenoxy-1,2-dioxetanes for excitation-free oxygen sensing^48,49^ and CL-induced PDT.^50^ However, their hydrophobicity and limited aqueous solubility have restricted their use in broader biomedical applications.

Shabat and co-workers introduced a supramolecular encapsulation strategy using trimethyl-β-cyclodextrin (TMCD) as a host for spiroadamantyl phenoxy-1,2-dioxetane probes.^51^ Typically, phenoxy-1,2-dioxetanes display low CL quantum yields in aqueous media due to efficient nonradiative decay of the chemiexcited benzoate intermediate via interactions with surrounding water molecules. Interestingly, TMCD encapsulation not only provides a local hydrophobic environment that protects the chemiexcited species from water but, when conjugated with a highly fluorescent dye, also enables efficient chemiluminescence resonance energy transfer (CRET), thereby amplifying CL intensity with possible CL color tuning. Notably, this supramolecular platform exhibits high modularity and allows facile derivatization of the spiroadamantyl phenoxy-1,2-dioxetane substrate with different recognition moieties serving as phenol protecting groups to target various biological triggers, while also permitting modulation of the energy acceptor for diverse imaging, therapeutic, or theranostic applications.

Building on these features, we hypothesized that functionalization of luminescent iridium­(III) complexes with a TMCD moiety will: 1) improve aqueous solubility and reduce dark cytotoxicity; and 2) facilitate the formation of stable host–guest adducts with spiroadamantyl phenoxy-1,2-dioxetane-based chemiluminescent probes (Scheme 1a), enabling CL-induced PDT via CRET-driven ROS generation under light-free conditions (Scheme 1b). Additionally, coupling stimuli-responsive chemiluminescent probes to iridium­(III) acceptors will enhance cancer selectivity via biomarker activation. The versatility of phenoxy-1,2-dioxetanes allows the targeting of a wide range of cancer biomarkers beyond the typical oxidants such as hydrogen peroxide (H_2_O_2_) that most CL-induced PDT platforms focus on.^52–57^ Although hydrophobic luminescent iridium­(III)–dioxetane constructs have been reported for CL imaging,^48,49^ water-soluble iridium­(III)–dioxetane systems tailored for CL-induced PDT have not been explored.

**Scheme 1:**
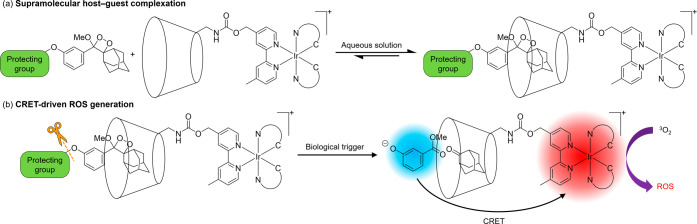
Schematic Diagrams Showing (a) Supramolecular Complexation between Spiroadamantyl Phenoxy-1,2-dioxetanes and Iridium­(III) TMCD Complexes, and (b) Biological Trigger-Initiated CL Activation and Subsequent CRET-Driven ROS Generation

In this work, we designed, synthesized, and characterized three luminescent and photofunctional cyclometalated iridium­(III) polypyridine complexes bearing a TMCD unit [Ir­(N^∧^C)_2_(bpy-TMCD)]­(Cl) (bpy-TMCD = 4-(*N*-trimethyl-β-cyclodextrin-mono-6-deoxy-6-amino­carbonyl­oxymethyl)-4′-methyl-2,2′-bipyridine; HN^∧^C = 2-phenylpyridine (Hppy) (**1a**), 2-phenylquinoline (Hpq) (**2a**), and 2-(1-naphthyl)­benzothiazole (Hbsn) (**3a**)), alongside their TMCD-free counterparts [Ir­(N^∧^C)_2_(bpy-C4)]­(Cl) (bpy-C4 = 4-(*N*-*n*-butylamino­carbonyl­oxymethyl)-4′-methyl-2,2′-bipyridine; HN^∧^C = Hppy (**1b**), Hpq (**2b**), and Hbsn (**3b**)) (Chart 1) for comparison. We evaluated their photophysical properties, singlet oxygen (^1^O_2_) photosensitization, cellular uptake and localization, and (photo)­cytotoxicity. We also utilized three enzyme-responsive spiroadamantyl phenoxy-1,2-dioxetane probes **ADO**_**P**_, **ADO**_**E**_, and **ADO**_**G**_ (Chart 2) targeting alkaline phosphatase (ALP), porcine liver esterase (PLE), and β-galactosidase (β-gal), respectively. In aqueous solutions, probes **ADO**_**P**_, **ADO**_**E**_, and **ADO**_**G**_ formed host–guest adducts with TMCD-functionalized complexes **1a** – **3a**, and these assemblies showed CRET from the probes to the iridium­(III) acceptors. We further examined enzyme-activated CRET-driven ROS generation and assessed the adducts as agents for CL-induced PDT in both 2D and 3D cell cultures.

**Chart 1:**
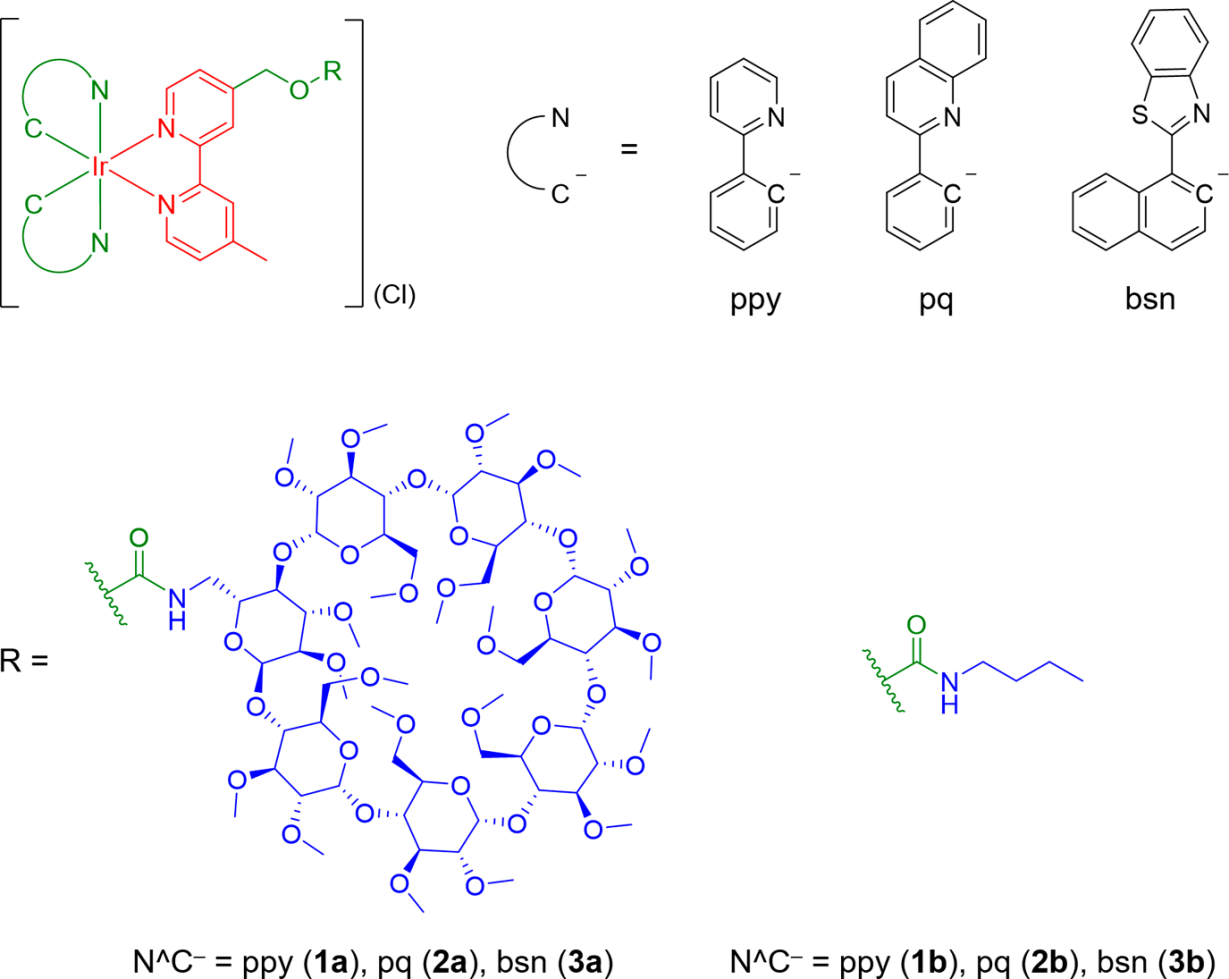
Structures of Iridium­(III) TMCD Complexes 1a – 3a and Their TMCD-Free Counterparts 1b – 3b

**Chart 2:**
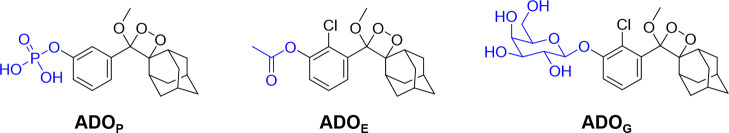
Structures of Probes ADO_P_, ADO_E_, and ADO_G_

## Results and Discussion

### Design and Synthesis

In designing the TMCD-functionalized complexes, cyclometalated iridium­(III) complexes were selected for their excellent photophysical and photochemical properties and tunable cellular behavior.^58–60^ We anticipate that appending a TMCD moiety to iridium­(III) complexes will not only improve aqueous solubility and reduce dark cytotoxicity, but also enable the formation of noncovalent host–guest adducts with chemiluminescent spiroadamantyl phenoxy-1,2-dioxetanes. Synthetic routes for the ligands and complexes are illustrated in Scheme 2. The ligand bpy-TMCD was obtained by coupling 4-(4-nitrophenyloxy­carbonyloxymethyl)-4′-methyl-2,2′-bipyridine (bpy-NPC)^61^ with permethylated 6-monoamino-6-monodeoxy-β-cyclodextrin (TMCD-NH_2_).^51^ Subsequent reaction of bpy-TMCD with the iridium­(III) dimers [Ir_2_(N^∧^C)_4_Cl_2_] (HN^∧^C = Hppy, Hpq, and Hbsn) in CH_2_Cl_2_/MeOH, followed by column chromatography, afforded complexes **1a** – **3a**. The TMCD-free ligand bpy-C4 was prepared by reacting bpy-NPC with *n*-butylamine.^62^ Complexes **1b** – **3b** were obtained by reacting [Ir_2_(N^∧^C)_4_Cl_2_] (HN^∧^C = Hppy, Hpq, and Hbsn) with bpy-C4 in CH_2_Cl_2_/MeOH and purified by column chromatography. All the complexes were isolated as air-stable yellow-to-red solids and characterized by HR-ESI-MS, ESI-MS, MALDI-TOF-MS, ^1^H and ^13^C NMR, and IR spectroscopy. Complexes **1b** – **3b** were soluble in common organic solvents such as CH_2_Cl_2_ and CH_3_CN, but displayed poor aqueous solubility, whereas the TMCD complexes **1a** – **3a** were soluble in both water and common organic solvents.

**Scheme 2:**
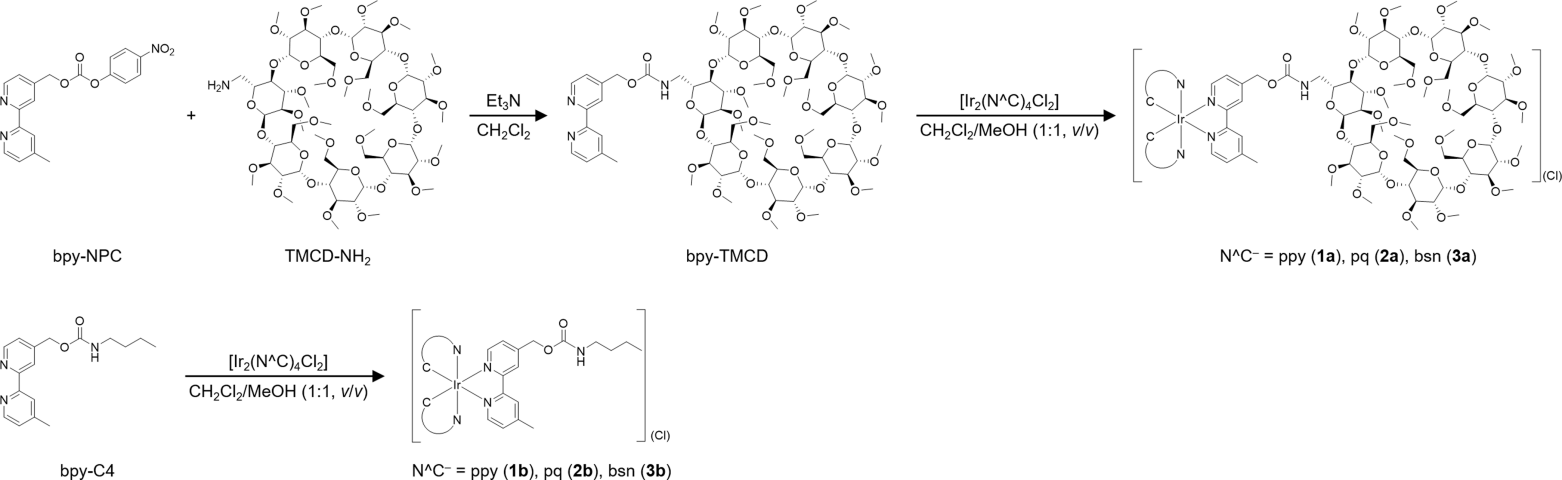
Synthetic Routes to Complexes 1a – 3a and 1b – 3b

Three enzyme-responsive spiroadamantyl phenoxy-1,2-dioxetane probes **ADO**_**P**_, **ADO**_**E**_, and **ADO**_**G**_ were selected for this study owing to their blue CL and enzyme-triggered selectivity in cancer cells. Probe **ADO**_**P**_ was obtained commercially. Probe **ADO**_**E**_ was synthesized by acetylation of **ADE**_**OH**_ ^63^ with acetic anhydride, followed by methylene blue-catalyzed photooxidation (Scheme 3), and was characterized by ESI-MS and ^1^H NMR spectroscopy. Probe **ADO**_**G**_ was prepared according to published procedures.^63,64^

**Scheme 3:**
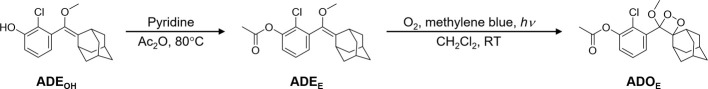
Synthetic Route to Probe ADO_E_

### Photophysical and Photosensitization Properties

The UV–vis absorption data for the complexes are summarized in Table S1, and the spectra are depicted in Figures 1a, S1, and S2. Consistent with related mixed-ligand cyclometalated iridium­(III) polypyridine systems, complexes **1a** – **3a** and **1b** – **3b** exhibited intense spin-allowed intraligand (^1^IL) (π → π*) (N^∧^N and N^∧^C) transitions at *ca*. 260–351 nm, along with weaker spin-allowed metal-to-ligand charge-transfer (^1^MLCT) (dπ­(Ir) → π*­(N^∧^N and N^∧^C)) features at *ca*. 381–475 nm.^62,65,66^ Additionally, much weaker tailing absorption beyond *ca*. 500 nm was attributed to spin-forbidden ^3^MLCT (dπ­(Ir) → π*­(N^∧^N and N^∧^C)) transitions. Upon photoexcitation, all the complexes showed intense greenish-yellow to red emission at ambient temperature and in low-temperature alcohol glass (Table 1 and Figures 1b, S3, and S4). The ppy complexes (**1a** and **1b**) displayed a broad and structureless emission band with positive solvatochromism in fluid solutions at 298 K. Upon cooling to 77 K, their emission maxima exhibited significant blue shifts, suggestive of a mixed triplet ^3^MLCT (dπ­(Ir) → π*­(N^∧^N and N^∧^C))/ligand-to-ligand charge-transfer (^3^LLCT) (π­(N^∧^C) → π*­(N^∧^N)) state.^62,65,66^ In contrast, the pq (**2a** and **2b**) and bsn (**3a** and **3b**) complexes showed vibronically structured emission features with longer emission lifetimes in fluid solutions at 298 K. Additionally, their emission properties were less sensitive to solvent polarity, indicating substantial ^3^IL (π → π*) (N^∧^C) character in their emissive states.^62,65,66^ However, the contribution of ^3^MLCT (dπ­(Ir) → π*­(N^∧^N)) character in the excited states of complex **2a** cannot be ruled out, as evidenced by the biexponential decay in H_2_O (Table 1). Notably, the emission quantum yields of the TMCD complexes **1a** – **3a** (Φ_em_ = 0.015–0.66 in degassed solvents; Table 1) are slightly lower than those of the TMCD-free complexes **1b** – **3b** (Φ_em_ = 0.029–0.71 in degassed solvents; Table 1), probably due to increased nonradiative decay pathways introduced by the appended TMCD unit.

**Figure 1:**
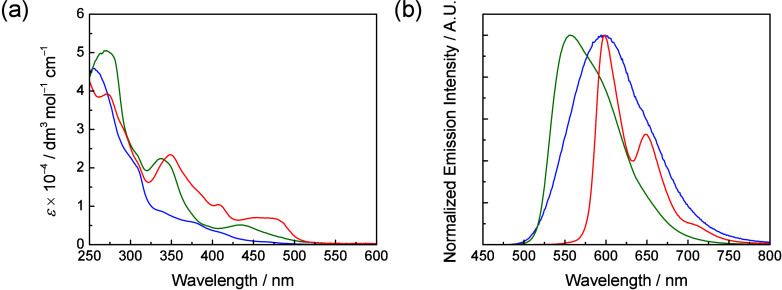
(a) UV–vis absorption spectra and (b) normalized emission spectra of complexes **1a** (blue), **2a** (green), and **3a** (red) in H_2_O at 298 K.

**Table 1:** Photophysical Data of Complexes 1a – 3a and 1b – 3b

Complex	Medium (*T*/K)	λ_em_/nm^a^	*τ*_o_/μs^b^	Φ_em_ ^c^
**1a**	CH_2_Cl_2_ (298)	587	0.57	0.26
	CH_3_CN (298)	608	0.34	0.10
	H_2_O (298)	599	0.05	0.015
	Glass^d^ (77)	517, 544 sh	4.85	
**2a**	CH_2_Cl_2_ (298)	558, 588 sh	2.47	0.66
	CH_3_CN (298)	561, 592 sh	2.58	0.60
	H_2_O (298)	557, 595 sh	1.68 (81%), 0.55 (19%)	0.35
	Glass^d^ (77)	543 (max), 587, 633 sh	5.28	
**3a**	CH_2_Cl_2_ (298)	595 (max), 646, 706 sh	3.90	0.21
	CH_3_CN (298)	595 (max), 647, 706 sh	3.82	0.18
	H_2_O (298)	598 (max), 649, 709 sh	3.38	0.10
	Glass^d^ (77)	591 (max), 609, 643, 663 sh, 704	6.00	
**1b**	CH_2_Cl_2_ (298)	588	0.59	0.28
	CH_3_CN (298)	607	0.35	0.14
	H_2_O^e^ (298)	590 (max), 652 sh	0.09	0.029
	Glass^d^ (77)	515, 543 sh	4.84	
**2b**	CH_2_Cl_2_ (298)	556, 587 sh	2.55	0.71
	CH_3_CN (298)	561, 592 sh	2.51	0.62
	H_2_O^e^ (298)	557 (max), 600 sh	1.91	0.44
	Glass^d^ (77)	543 (max), 585, 633 sh	5.07	
**3b**	CH_2_Cl_2_ (298)	596 (max), 647, 708 sh	3.92	0.23
	CH_3_CN (298)	596 (max), 647, 709 sh	3.56	0.21
	H_2_O^e^ (298)	541 sh, 594 (max), 644, 698 sh	2.86	0.071
	Glass^d^ (77)	591 (max), 609, 641, 662 sh, 704	5.73	

a *λ*_ex_ = 350 nm.

b The lifetimes were measured at the emission maxima (*λ*_ex_ = 355 nm).

c [Ru­(bpy)_3_]­Cl_2_ was used as the reference (Φ_em_ = 0.040 in aerated H_2_O, *λ*_ex_ = 455 nm).^67^

d EtOH/MeOH (4:1, *v*/*v*).

e H_2_O/MeOH (7:3, *v*/*v*).

The ^1^O_2_ generation quantum yields (Φ_Δ_) of the complexes in aerated CH_3_CN were examined using [Ru­(bpy)_3_]­Cl_2_ (Φ_Δ_ = 0.57)^68^ as a reference (Table 2). The Φ_Δ_ values of the TMCD complexes **1a** – **3a** (0.45–0.87) are comparable to those of their TMCD-free counterparts **1b** – **3b** (0.46–0.83), suggesting that the TMCD group exerts minimal influence. The highest ^1^O_2_ generation quantum yields of the bsn complexes **3a** and **3b** are attributed to their longer-lived triplet excited states.

**Table 2:** ^1^O_2_ Generation Quantum Yields of Complexes 1a – 3a and 1b – 3b in Aerated CH_3_CN at 298 K

Complex	Φ_Δ_ ^a^
**1a**	0.45
**2a**	0.67
**3a**	0.87
**1b**	0.46
**2b**	0.63
**3b**	0.83

a *λ*_ex_ = 450 nm and [Ru­(bpy)_3_]­Cl_2_ was used as the reference (Φ_Δ_ = 0.57 in aerated CH_3_CN).^68^

### Cellular Uptake and (Photo)­cytotoxicity Studies

We evaluated the cellular uptake of the iridium­(III) complexes in HeLa cells by inductively coupled plasma-mass spectrometry (ICP-MS) measurements. All the complexes displayed efficient accumulation after 2 h of incubation, and the intracellular iridium levels followed the order: **3a** > **2a** > **1a** and **3b** > **2b** > **1b** (Table 3), consistent with the hydrophobicity of the cyclometalating ligands (bsn > pq > ppy). The uptake of the TMCD complexes **1a** – **3a** ([Ir] = 0.088–0.76 fmol) was lower than that of their TMCD-free counterparts **1b** – **3b** ([Ir] = 1.5–4.6 fmol), which should be a consequence of the increased molecular size imparted by the TMCD moiety.

**Table 3:** Uptake of Complexes 1a – 3a and 1b – 3b in HeLa Cells

Complex	Amount of iridium/fmol^a^
**1a**	0.088 ± 0.007
**2a**	0.47 ± 0.05
**3a**	0.76 ± 0.06
**1b**	1.5 ± 0.1
**2b**	1.7 ± 0.1
**3b**	4.6 ± 0.2

a Amount of iridium associated with an average HeLa cell upon incubation with the complexes (5 μM) at 37 °C for 2 h, as determined by ICP-MS.

We then investigated the (photo)­cytotoxicity of the complexes in HeLa cells using the MTT assay. In the dark, the TMCD complexes **1a** – **3a** exhibited lower cytotoxicity (IC_50,dark_ = 14–590 μM; Table 4) than the TMCD-free complexes **1b** – **3b** (IC_50,dark_ = 6.7–18 μM; Table 4). The reduced dark cytotoxicity of complexes **1a** – **3a** is attributed to the presence of a TMCD unit, which decreased cellular uptake (Table 3) and hindered interactions with intracellular biomolecules. Upon irradiation, the cytotoxicity of the TMCD complexes increased markedly with the IC_50,light_ values decreasing sharply (Table 4), most likely due to efficient photoinduced formation of ^1^O_2_. The highest photocytotoxicity observed for complex **3a** among the TMCD complexes is attributable to its efficient cellular uptake (Table 3) and high ^1^O_2_ generation quantum yield (Φ_Δ_ = 0.87; Table 2). Remarkably, complexes **2a** and **3a** showed significant photocytotoxicity coupled with relatively low dark cytotoxicity, yielding high photocytotoxicity indices of 388 and 483, respectively, which suggests strong potential as PDT agents. By contrast, although complexes **2b** and **3b** also showed notable photocytotoxic activity and high photocytotoxicity indices, their considerable dark cytotoxicity may limit their applicability in biological systems.

**Table 4:** Cytotoxicity of Complexes 1a – 3a and 1b – 3b toward HeLa Cells under Dark or Light Conditions (*λ*_ex_ = 450 nm, 15.5 mW cm^–2^, 10 min)

Complex	IC_50,dark_/μM	IC_50,light_/μM	Photocytotoxicity index^a^
**1a**	590 ± 8	16 ± 1	37
**2a**	31 ± 1	0.080 ± 0.001	388
**3a**	14 ± 1	0.029 ± 0.001	483
**1b**	18 ± 1	0.17 ± 0.01	106
**2b**	7.6 ± 0.1	0.010 ± 0.001	760
**3b**	6.7 ± 0.6	0.009 ± 0.001	744

a Photocytotoxicity index is defined as IC_50,dark_ /IC_50,light_.

### Live-Cell Confocal Imaging

The localization of the complexes in HeLa cells was examined by laser-scanning confocal microscopy (LSCM). After incubation with complexes **1a** – **3a** and **1b** – **3b**, the cells displayed strong intracellular emission (Figures 2 and 3). Co-staining experiments with MitoTracker Deep Red confirmed mitochondrial localization, with Pearson’s correlation coefficients (PCCs) of 0.84–0.96 (Figures 2 and 3). The pronounced mitochondrial specificity of complexes **1a** – **3a** and **1b** – **3b** is attributed to their monocationic and lipophilic character.^6,69,70^

**Figure 2:**
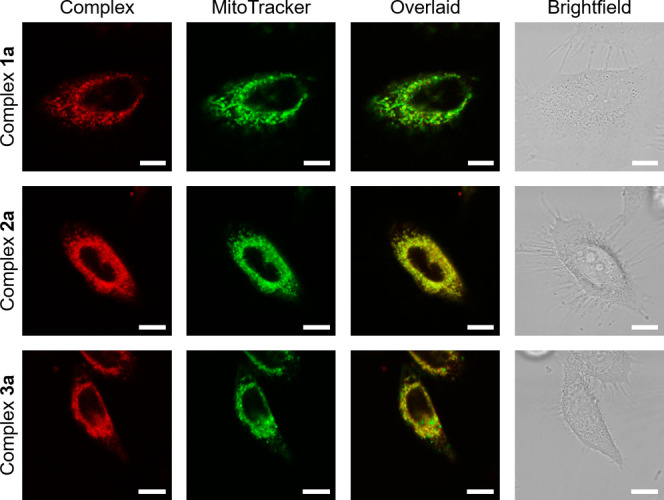
LSCM images of live HeLa cells incubated with complex **1a** (20 μM, 2 h), complex **2a** (5 μM, 2 h), or complex **3a** (5 μM, 2 h), and stained with MitoTracker Deep Red (100 nM, 20 min). Complexes **1a** – **3a**: *λ*_ex_ = 405 nm, *λ*_em_ = 550–650 nm. MitoTracker Deep Red: *λ*_ex_ = 635 nm, *λ*_em_ = 650–680 nm. PCC = 0.86 (complex **1a**), 0.96 (complex **2a**), and 0.84 (complex **3a**). Scale bar = 10 μm.

**Figure 3:**
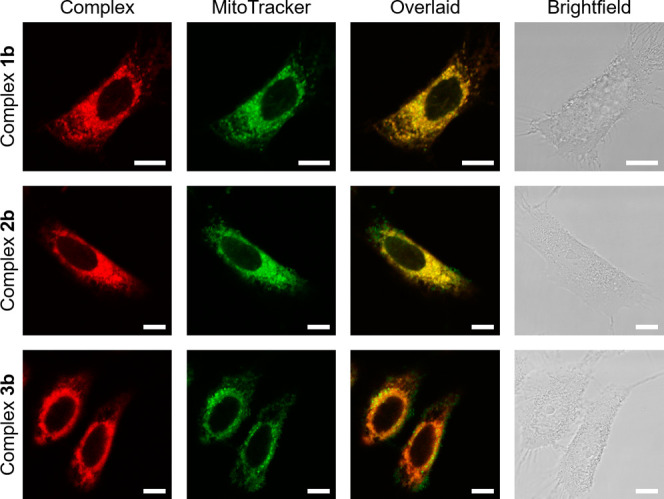
LSCM images of live HeLa cells incubated with complexes **1b** – **3b** (5 μM, 2 h), and stained with MitoTracker Deep Red (100 nM, 20 min). Complexes **1b** – **3b**: *λ*_ex_ = 405 nm, *λ*_em_ = 550–650 nm. MitoTracker Deep Red: *λ*_ex_ = 635 nm, *λ*_em_ = 650–680 nm. PCC = 0.95 (complex **1b**), 0.96 (complex **2b**), and 0.88 (complex **3b**). Scale bar = 10 μm.

### Chemiluminescence Resonance Energy Transfer (CRET)

It has been established that CRET can be achieved between chemiluminescent spiroadamantyl phenoxy-1,2-dioxetanes and TMCD-modified fluorescent dyes, facilitated by supramolecular interaction between adamantane and TMCD.^51^ Thus, we examined the interaction between spiroadamantyl phenoxy-1,2-dioxetanes and the TMCD-containing iridium­(III) complexes using probe **ADO**_**P**_ and complex **1a** as models. ^31^P NMR titration experiments were conducted using a fixed concentration of the chemiluminescent probe **ADO**_**P**_ (4 mM) and varying concentrations of complex **1a** (0.5–4 mM) in D_2_O. Increasing the concentration of complex **1a** induced a progressive upfield shift in the ^31^P resonance of the phosphate group of the probe (Figure S5), indicating inclusion complex formation between **ADO**_**P**_ and complex **1a**. Fitting the titration data using the Benesi–Hildebrand equation afforded a linear plot of 1/Δδ against 1/[**1a**] (Figure S6a), indicating a 1:1 host–guest complexation stoichiometry. The association constant (*K*_a_) was determined to be 192.9 ± 20.9 M^–1^ at 298 K, which is comparable to the previously reported host–guest system involving **ADO**_**P**_ and TMCD (*K*_a_ = 253 M^–1^).^51^ Similar values were also obtained for probe **ADO**_**P**_ with complexes **2a** and **3a** (*K*_a_ = 260.3 ± 43.9 and 214.0 ± 22.3 M^–1^, respectively; Figure S6b and c).

As the TMCD-functionalized iridium­(III) complexes formed host–guest adducts with spiroadamantyl phenoxy-1,2-dioxetanes, we explored the possibility of CRET occurring from probes **ADO**_**P**_ – **ADO**_**G**_ to complexes **1a** – **3a**. Probe **ADO**_**P**_ was selected as the model due to its relatively high aqueous solubility. The CL spectrum of **ADO**_**P**_ treated with ALP, and the absorption spectra of complexes **1a** – **3a** were compared to evaluate spectral overlap (Figure 4), which is essential for CRET to take place. Additionally, the overlap integral (*J*) was calculated from the spectral data. The results show that among the three TMCD complexes, complex **1a** exhibited the smallest spectral overlap with **ADO**_**P**_ (*J* = 2.18 × 10^15^ M^–1^ cm^–1^ nm^4^; Figure 4a), suggesting the lowest CRET efficiency between the two entities. In contrast, complexes **2a** and **3a** displayed a larger spectral overlap with **ADO**_**P**_ (*J* = 9.54 × 10^15^ and 19.66 × 10^15^ M^–1^ cm^–1^ nm^4^, respectively; Figure 4b and c), indicating a high likelihood of efficient CRET.

**Figure 4:**
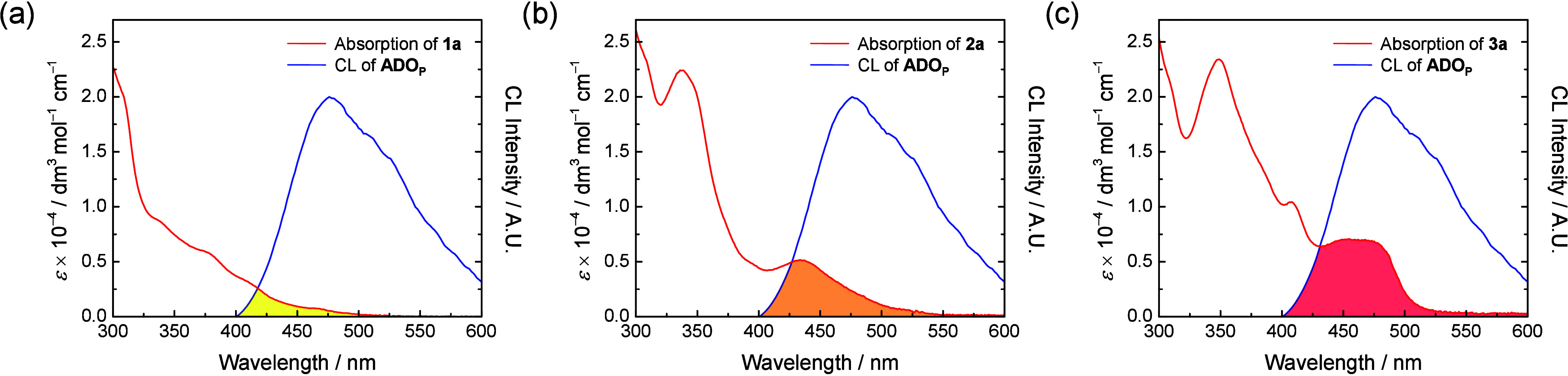
Absorption spectra (red) of (a) complex **1a**, (b) complex **2a**, and (c) complex **3a** in H_2_O overlaid with the CL spectrum of probe **ADO**_**P**_ (blue) in Tris buffer (50 mM, pH 9.0) at 298 K in the presence of ALP (1 unit mL^–1^). Shaded regions indicate spectral overlap.

Next, we measured the CL spectrum of probe **ADO**_**P**_ (100 μM) and compared it to those of the host–guest adducts **ADO**_**P**_ **–1a**, **ADO**_**P**_ **–2a**, and **ADO**_**P**_ **–3a** ([**ADO**_**P**_] = 100 μM, [Ir] = 100 μM). These measurements were performed in the presence of ALP (1 unit mL^–1^) in Tris buffer (50 mM) at pH 9.0 at 298 K, where the activity of ALP is enhanced and thereby favors CL induction. Probe **ADO**_**P**_ alone exhibited blue CL (λ_max_ = 478 nm) (Figure 5a) upon ALP activation. Interestingly, adduct **ADO**_**P**_ **–1a** showed an additional emission peak at 536 nm in its CL spectrum (Figure 5b), characteristic of the emission from the ppy complex **1a**, indicating successful CRET from **ADO**_**P**_ to complex **1a**. Quantitative determination of the CRET efficiency between the two entities, calculated as the ratio of the acceptor emission integral to the total emission integral under chemiexcitation,^71^ revealed a moderate CRET efficiency of 75%, likely due to the relatively small spectral overlap between **ADO**_**P**_ and complex **1a** (Figure 4a). For comparison, we also measured the CL spectrum of a solution mixture of probe **ADO**_**P**_ (100 μM), TMCD (100 μM), and the TMCD-free complex **1b** (100 μM) in the presence of ALP (1 unit mL^–1^) (Figure 5c). Notably, the intensity of the iridium­(III) emission was lower than that of the intrinsic CL from **ADO**_**P**_. The lower CRET efficiency (48%) between **ADO**_**P**_ and complex **1b** is attributed to the absence of a covalently linked TMCD moiety to bring the two entities into close proximity for efficient CRET. Remarkably, the CL spectra of adducts **ADO**_**P**_ **–2a** and **ADO**_**P**_ **–3a** displayed one single emission band at 564 and 604 nm (Figure 5d and e, black), respectively, corresponding to the emission of pq complex **2a** and bsn complex **3a**. The absence of **ADO**_**P**_ emission peak at 480 nm suggests that CRET between **ADO**_**P**_ and complexes **2a** and **3a** was highly efficient (>99%) under our experimental conditions, consistent with their larger spectral overlap with the CL of **ADO**_**P**_ (Figure 4b and c). A single emission band was also observed for solution mixtures containing probe **ADO**_**P**_ (100 μM), TMCD (100 μM), and TMCD-free complex **2b** or **3b** (100 μM) (Figure 5d and e, red), but the intensities of their respective iridium­(III) emission were substantially lower than those in adducts **ADO**_**P**_ **–2a** and **ADO**_**P**_ **–3a**. Importantly, negligible emission was observed for probe **ADO**_**P**_ and adducts **ADO**_**P**_ **–1a**, **ADO**_**P**_ **–2a**, and **ADO**_**P**_ **–3a** in the absence of ALP (Figure S7), confirming ALP as the specific trigger for CL activation. These results demonstrate that CRET occurred from **ADO**_**P**_ to complexes **1a** – **3a** upon ALP activation, and highlight the important role of TMCD modification in positioning the iridium­(III) acceptor in close proximity to the chemiexcited donor via supramolecular host–guest binding, enabling efficient CRET that enhanced CL intensity and red-shifted CL color. We also measured the CL spectrum of the host–guest adduct formed between probe **ADO**_**P**_ and the rhodium­(III) counterpart of complex **3a**, [Rh­(bsn)_2_(bpy-TMCD)]­(Cl) (**3c**), which shared high physical and chemical similarities with its iridium­(III) congener but lacked intense photoluminescence.^72^ Despite spectral overlap, the resulting adduct **ADO**_**P**_ **–3c** exhibited very weak CL response, underscoring the necessity of a strongly luminescent complex as the energy acceptor for CL enhancement with possible CL color tuning.

**Figure 5:**
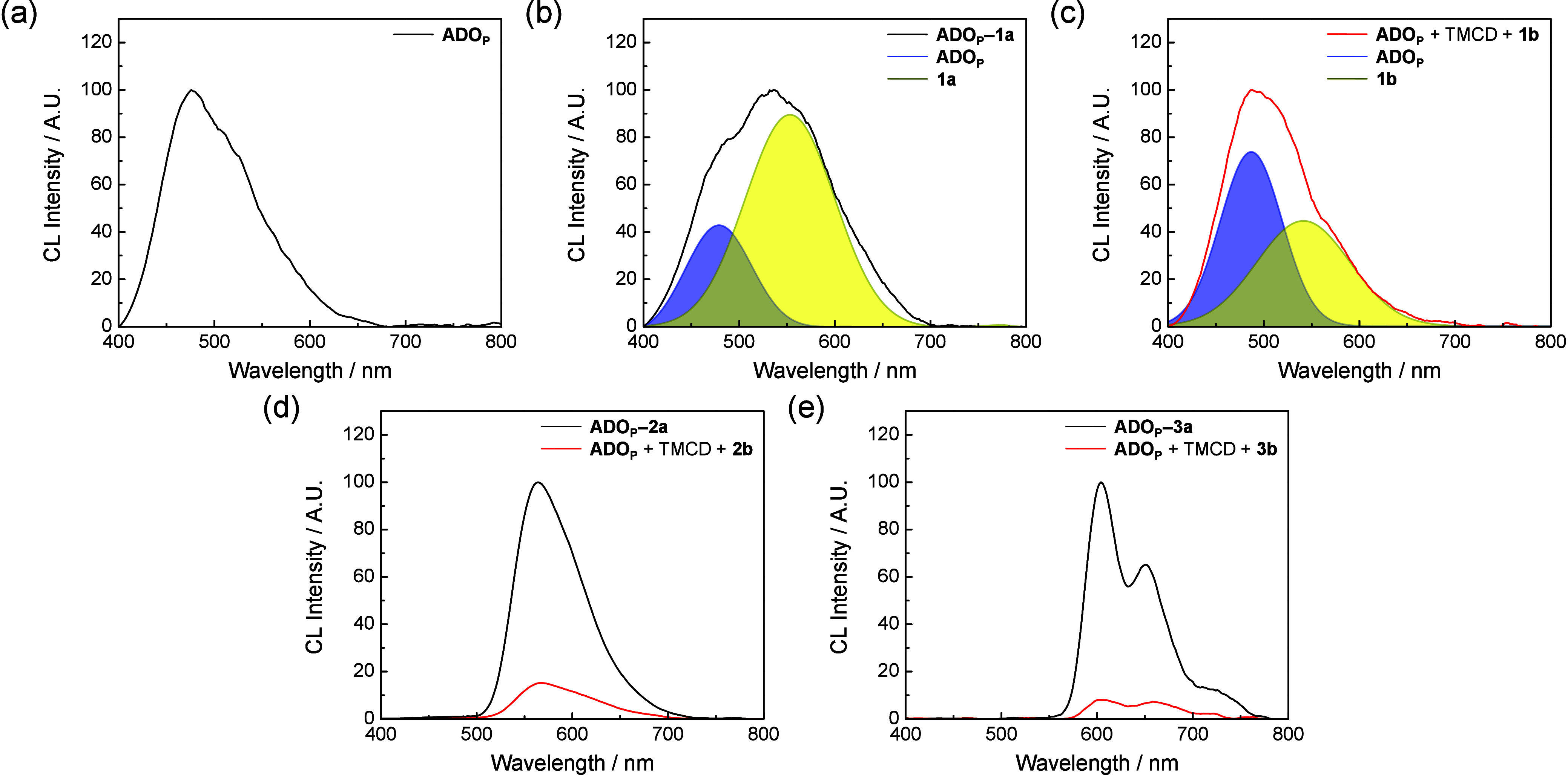
CL spectra of (a) probe **ADO**_**P**_, (b) adduct **ADO**_**P**_ **–1a**, (c) **ADO**_**P**_/TMCD/complex **1b** mixture, (d) adduct **ADO**_**P**_ **–2a** (black) and **ADO**_**P**_/TMCD/complex **2b** mixture (red), and (e) adduct **ADO**_**P**_ **–3a** (black) and **ADO**_**P**_/TMCD/complex **3b** mixture (red) in Tris buffer (50 mM, pH 9.0) in the presence of ALP (1 unit mL^–1^). [**ADO**_**P**_] = 100 μM, [TMCD] = 100 μM, [Ir] = 100 μM. Shaded curves represent deconvoluted luminescence spectra.

### Enzyme-Triggered CL Responses

To examine the CL changes of probe **ADO**_**P**_ when bound to the TMCD complexes **1a** – **3a**, we compared the CL kinetic profiles of **ADO**_**P**_ (10 μM) with and without TMCD or complexes **1a** – **3a** (100 μM). The kinetic profile of **ADO**_**P**_ in the presence of ALP showed characteristic CL behavior, reaching peak intensity before gradually decaying to baseline (Figure 6a, blue). Its CL quantum yield (Φ_CL_) and half-life (*t*_1/2_) were determined to be 1.12 × 10^–2^ Einstein mol^–1^ and 22.7 min (Table S2), respectively. Upon activation of adduct **ADO**_**P**_–TMCD by ALP, we observed a slight increase in total photons emitted from **ADO**_**P**_ (Figure 6, green), with the Φ_CL_ and *t*_1/2_ values increasing to 1.58 × 10^–2^ Einstein mol^–1^ and 60.7 min (Table S2), respectively, which is attributable to the TMCD encapsulation effect.^51^ Adduct **ADO**_**P**_ **–1a** displayed no significant changes in total light emission upon ALP activation (Φ_CL_ = 1.31 × 10^–2^ Einstein mol^–1^, *t*_1/2_ = 37.5 min; Table S2 and Figure 6, black) relative to adduct **ADO**_**P**_–TMCD, due to modest CRET from **ADO**_**P**_ to complex **1a** (75%) and the relatively low emission quantum yield of the ppy complex **1a** (Φ_em_ = 0.015 in degassed H_2_O; Table 1). Remarkably, adducts **ADO**_**P**_ **–2a** and **ADO**_**P**_ **–3a** exhibited substantially enhanced CL efficiencies (Figure 6, orange and red) with Φ_CL_ values of 28.43 × 10^–2^ and 6.01 × 10^–2^ Einstein mol^–1^ (Table S2), respectively, which are 25.4- and 5.4-fold larger than that of **ADO**_**P**_ (Φ_CL_ = 1.12 × 10^–2^ Einstein mol^–1^). The CL half-lives of adducts **ADO**_**P**_ **–2a** and **ADO**_**P**_ **–3a** were also extended to 84.0 and 83.9 min, respectively (Table S2). It is conceivable that the high CRET efficiency (>99%) between **ADO**_**P**_ and complex **2a**, along with the high emission quantum yield of the pq complex **2a** (Φ_em_ = 0.35 in degassed H_2_O; Table 1), allows adduct **ADO**_**P**_ **–2a** to act as an efficient CL enhancer applicable for biological imaging. For adduct **ADO**_**P**_ **–3a**, despite smaller CL enhancement, the high ^1^O_2_ generation quantum yield of the bsn complex **3a** (Φ_Δ_ = 0.87 in aerated CH_3_CN; Table 2) suggests potential applications in CL-induced PDT. Crucially, no similar CL enhancement was observed for the host–guest adducts **ADO**_**P**_–TMCD, **ADO**_**P**_ **–1a**, **ADO**_**P**_ **–2a**, and **ADO**_**P**_ **–3a** when ALP was pretreated with phosphatase inhibitor cocktail III (PIC3) (Figure S8), further substantiating ALP as the specific trigger for CL activation.

**Figure 6:**
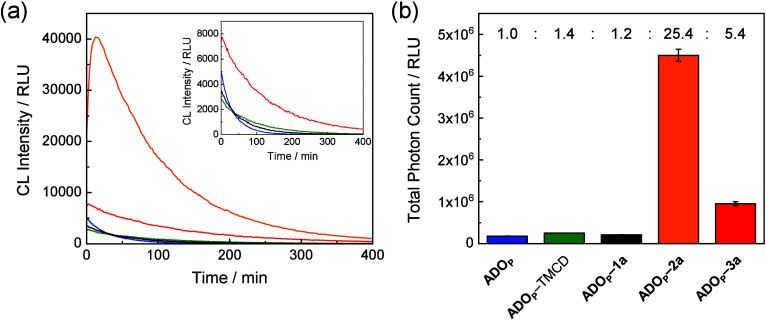
(a) CL kinetic profiles and (b) total photon counts for probe **ADO**_**P**_ (10 μM) (blue), adduct **ADO**_**P**_–TMCD ([**ADO**_**P**_] = 10 μM, [TMCD] = 100 μM) (green), adduct **ADO**_**P**_ **–1a** (black), adduct **ADO**_**P**_ **–2a** (orange), and adduct **ADO**_**P**_ **–3a** (red) ([**ADO**_**P**_] = 10 μM, [Ir] = 100 μM) in Tris buffer (50 mM, pH 9.0) at 298 K in the presence of ALP (1 unit mL^–1^). Inset: Enlarged view of the CL kinetic profiles for probe **ADO**_**P**_ (blue), adduct **ADO**_**P**_–TMCD (green), adduct **ADO**_**P**_ **–1a** (black), and adduct **ADO**_**P**_ **–3a** (red) in the presence of ALP.

The CL activation mechanism of spiroadamantyl phenoxy-1,2-dioxetane probes relies on the cleavage of protecting groups from the phenolic unit, allowing different phenol protecting groups to serve as enzyme-specific triggering substrates. To expand detection capabilities, we designed two new probes targeting different enzymes: PLE and β-gal. Specifically, probe **ADO**_**E**_ was functionalized with an acetyl group for esterase recognition, while probe **ADO**_**G**_ incorporated a galactopyranoside as a β-gal substrate (Chart 2). Both **ADO**_**E**_ and **ADO**_**G**_ were modified with an *ortho*-chlorine substituent adjacent to the phenolic oxygen, unlike probe **ADO**_**P**_. This chlorine substitution was strategically introduced to lower the p*K*_a_ of the liberated phenol following enzymatic cleavage, thereby facilitating the chemiexcitation process of 1,2-dioxetane under physiological conditions.^64^ A comparative evaluation of CL signals was performed for **ADO**_**E**_ and **ADO**_**G**_ in the absence and presence of TMCD or TMCD-modified iridium­(III) complexes **1a** – **3a** (Figure 7). Enzymes including PLE and β-gal were employed to activate **ADO**_**E**_ or **ADO**_**G**_, respectively, in phosphate-buffered saline (PBS) at pH 7.4, an optimal pH for their catalytic function. Upon PLE or β-gal activation, **ADO**_**E**_ and **ADO**_**G**_ showed minimal intrinsic CL (Figure 7, blue) due to water quenching. Inclusion of these probes in TMCD induced small CL enhancements of 2.4- and 1.5-fold, respectively (Figure 7, green). Similar increases were also observed for adducts **ADO**_**E**_ **–1a** (1.9-fold) and **ADO**_**G**_ **–1a** (1.3-fold) when reacted with their corresponding enzymes (Figure 7, black). Notably, the CL efficiencies of **ADO**_**E**_ and **ADO**_**G**_ increased significantly in the presence of complex **2a** or **3a**; adducts **ADO**_**E**_ **–2a** and **ADO**_**G**_ **–2a** displayed 44.0- and 28.9-fold CL enhancement (Figure 7, orange), while adducts **ADO**_**E**_ **–3a** and **ADO**_**G**_ **–3a** exhibited 5.6- and 4.9-fold increases (Figure 7, red) upon activation by PLE and β-gal, respectively. Importantly, the CL efficiencies of adducts **ADO**_**E**_ **–2a**, **ADO**_**E**_ **–3a**, **ADO**_**G**_ **–2a**, and **ADO**_**G**_ **–3a** remained low when PLE and β-gal were pretreated with their respective inhibitors bis­(4-nitrophenyl) phosphate (BNPP)^73^ (10 mM; Figure S9) and ethylenediaminetetraacetic acid (EDTA)^74^ (20 mM; Figure S10), providing further support that enzymatic reactions serve as the specific triggers for CL induction.

**Figure 7:**
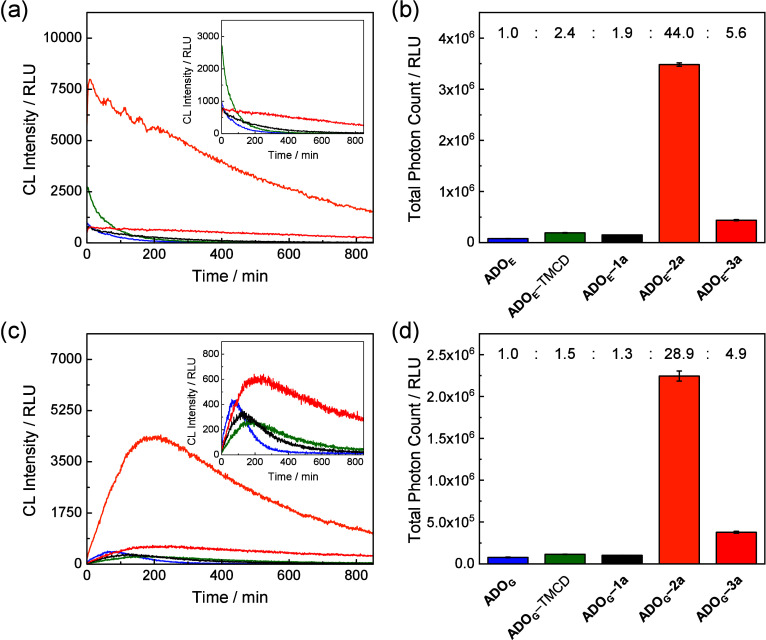
(a) CL kinetic profiles and (b) total photon counts for probe **ADO**_**E**_ (10 μM) (blue), adduct **ADO**_**E**_–TMCD ([**ADO**_**E**_] = 10 μM, [TMCD] = 100 μM) (green), adduct **ADO**_**E**_ **–1a** (black), adduct **ADO**_**E**_ **–2a** (orange), and adduct **ADO**_**E**_ **–3a** (red) ([**ADO**_**E**_] = 10 μM, [Ir] = 100 μM) in PBS (1X, pH 7.4) at 298 K in the presence of PLE (1 unit mL^–1^). Inset: Enlarged view of the CL kinetic profiles for probe **ADO**_**E**_ (blue), adduct **ADO**_**E**_–TMCD (green), adduct **ADO**_**E**_ **–1a** (black), and adduct **ADO**_**E**_ **–3a** (red) in the presence of PLE. (c) CL kinetic profiles and (d) total photon counts for probe **ADO**_**G**_ (10 μM) (blue), adduct **ADO**_**G**_–TMCD ([**ADO**_**G**_] = 10 μM, [TMCD] = 100 μM) (green), adduct **ADO**_**G**_ **–1a** (black), adduct **ADO**_**G**_ **–2a** (orange), and adduct **ADO**_**G**_ **–3a** (red) ([**ADO**_**G**_] = 10 μM, [Ir] = 100 μM) in PBS (1X, pH 7.4) at 298 K in the presence of β-gal (1 unit mL^–1^). Inset: Enlarged view of the CL kinetic profiles for probe **ADO**_**G**_ (blue), adduct **ADO**_**G**_–TMCD (green), adduct **ADO**_**G**_ **–1a** (black), and adduct **ADO**_**G**_ **–3a** (red) in the presence of β-gal.

To further confirm that the CL amplification is driven by noncovalent supramolecular host–guest interaction, we selected probe **ADO**_**E**_ for further studies due to its largest CL enhancement in the presence of the TMCD complexes. While adducts **ADO**_**E**_–TMCD, **ADO**_**E**_ **–1a**, **ADO**_**E**_ **–2a**, and **ADO**_**E**_ **–3a** showed strong CL signals upon PLE activation, addition of excess 1-adamantanemethylamine (AD) (1 mM) to the solutions significantly reduced the CL of these adducts (Figures 8 and S11). The pronounced attenuation indicates that free AD competitively disrupted the inclusion complex and significantly reduced CL intensity, directly linking the CL amplification effect to supramolecular complexation between the spiroadamantyl phenoxy-1,2-dioxetane probes and the TMCD moiety of complexes **1a** – **3a**, which brings the two entities into close proximity to facilitate CRET.

**Figure 8:**
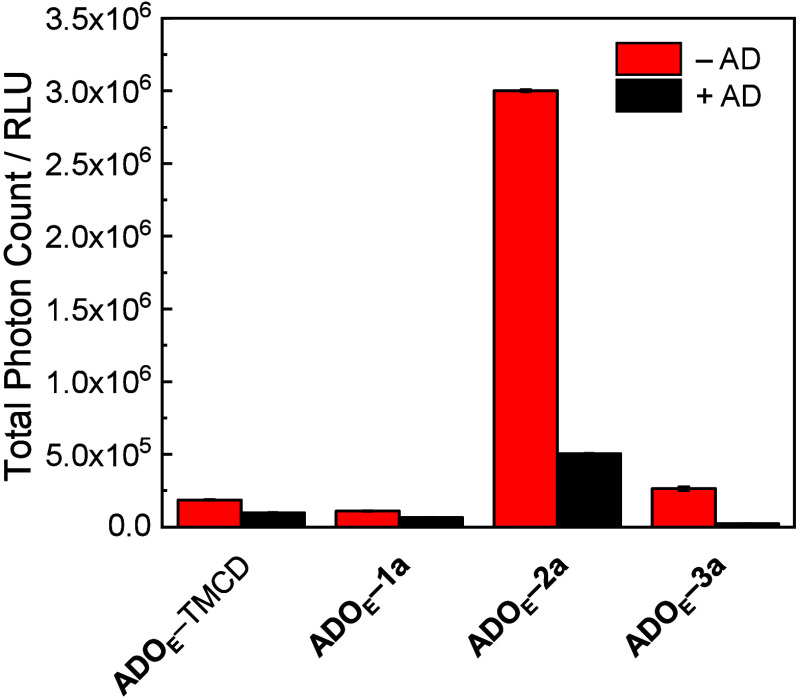
Total photon counts for adduct **ADO**_**E**_–TMCD ([**ADO**_**E**_] = 10 μM, [TMCD] = 100 μM), adduct **ADO**_**E**_ **–1a**, adduct **ADO**_**E**_ **–2a**, and adduct **ADO**_**E**_ **–3a** ([**ADO**_**E**_] = 10 μM, [Ir] = 100 μM) in PBS (1X, pH 7.4) in the presence of PLE (1 unit mL^–1^) without (red) or with (black) the addition of AD (1 mM).

### CRET-Induced ROS Generation *In Vitro*

Since many luminescent iridium­(III) complexes are efficient ROS photosensitizers, we investigated whether CRET from probes **ADO**_**P**_ – **ADO**_**G**_ to complexes **1a** – **3a** in the adducts can induce ROS formation. We selected probe **ADO**_**P**_ for its excellent aqueous solubility and complex **3a** for its efficient CRET from **ADO**_**P**_ (>99%) and high ^1^O_2_ generation quantum yield (Table 2). The generation of ROS was assessed using 2′,7′-dichlorodihydrofluorescein (DCFH_2_), which is oxidized to fluorescent 2′,7′-dichlorofluorescein (DCF) upon exposure to ROS. A mixture of **ADO**_**P**_ (500 μM) and complex **3a** (5 μM) in PBS (1X, pH 7.4) was supplemented with DCFH_2_ (20 μM). Fluorescence was measured at 1-h intervals, with the samples protected from light between readings. Upon addition of ALP (1 unit mL^–1^), the fluorescence at 525 nm increased substantially over time (Figure 9a and c, red). However, only negligible fluorescence increase was observed when ALP was absent (Figure 9b and c, black), attributed to the direct excitation of complex **3a** during measurements, which produced minimal ROS. These results indicate that ROS production occurred under light-free conditions via CRET.

**Figure 9:**
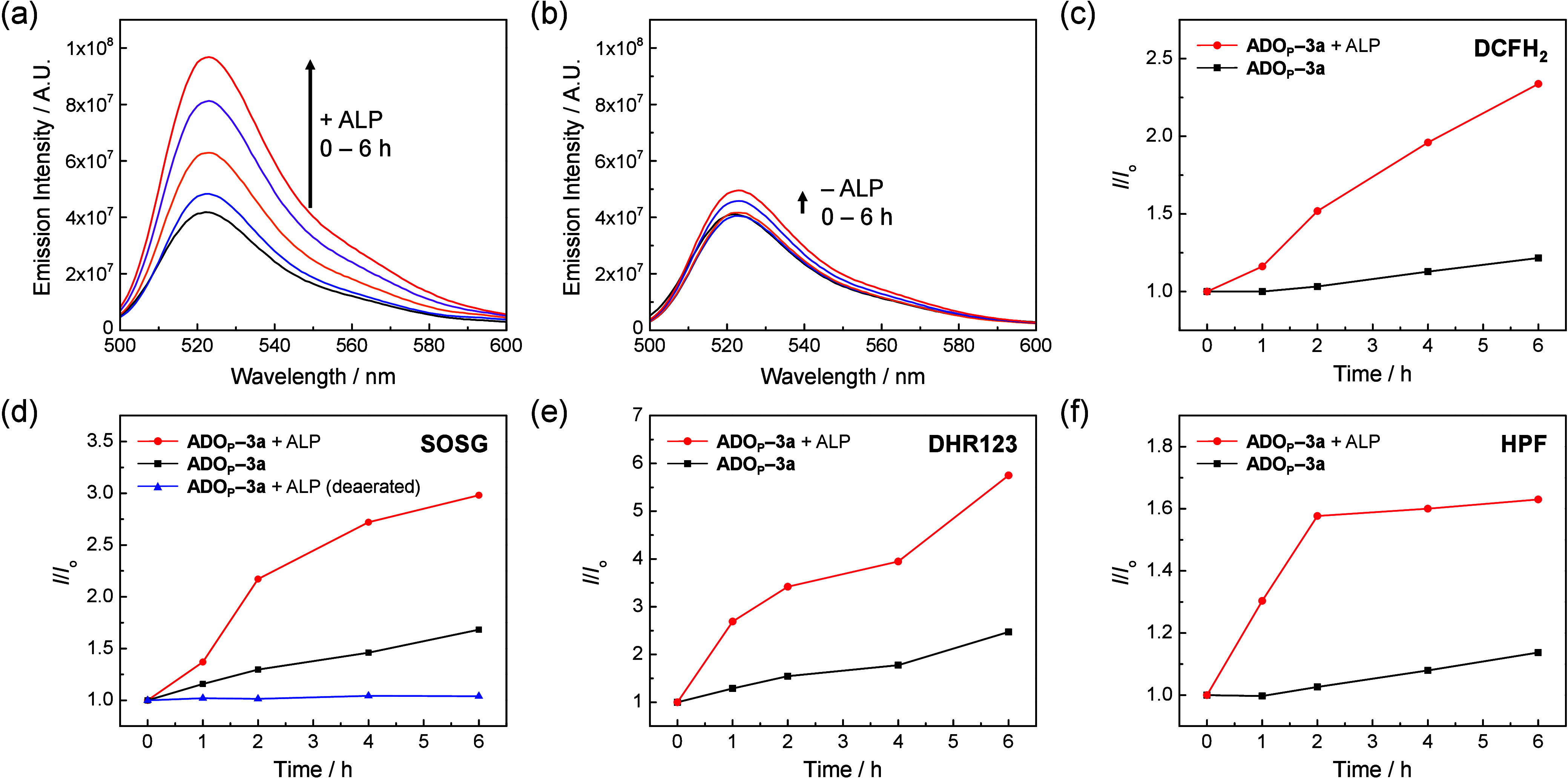
Time-dependent fluorescence spectra of DCFH_2_ (20 μM) treated with adduct **ADO**_**P**_ **–3a** ([**ADO**_**P**_] = 500 μM, [**3a**] = 5 μM) in aerated PBS (1X, pH 7.4) at 298 K, recorded from 0 to 6 h (a) upon the addition of ALP (1 unit mL^–1^) or (b) without ALP. (c) Changes in the fluorescence intensity of DCFH_2_ (20 μM; λ_em_ = 525 nm) treated with adduct **ADO**_**P**_ **–3a** ([**ADO**_**P**_] = 500 μM, [**3a**] = 5 μM) in aerated PBS (1X, pH 7.4) at 298 K, recorded from 0 to 6 h upon the addition of ALP (1 unit mL^–1^) (red) or without ALP (black). (d) Changes in the fluorescence intensity of SOSG (10 μM; λ_em_ = 530 nm) treated with adduct **ADO**_**P**_ **–3a** ([**ADO**_**P**_] = 500 μM, [**3a**] = 5 μM) in PBS (1X, pH 7.4) at 298 K, recorded from 0 to 6 h upon the addition of ALP (1 unit mL^–1^) under aerated (red) or deaerated (blue) conditions, or without ALP under aerated conditions (black). Changes in the fluorescence intensity of (e) DHR123 (10 μM; λ_em_ = 530 nm) and (f) HPF (10 μM; λ_em_ = 515 nm) treated with adduct **ADO**_**P**_ **–3a** ([**ADO**_**P**_] = 500 μM, [**3a**] = 5 μM) in aerated PBS (1X, pH 7.4) at 298 K, recorded from 0 to 6 h upon the addition of ALP (1 unit mL^–1^) (red) or without ALP (black).

To verify ^1^O_2_ generation under light-free conditions, we employed singlet oxygen sensor green (SOSG), a fluorogenic probe selective for ^1^O_2_ over other ROS. Similarly, a time-dependent fluorescence increase was observed in samples containing probe **ADO**_**P**_, complex **3a**, ALP, and SOSG (Figure 9d, red), while much weaker signals were detected in the absence of ALP (Figure 9d, black), indicating ^1^O_2_ production by adduct **ADO**_**P**_ **–3a** upon ALP activation. Importantly, SOSG fluorescence enhancement was substantially reduced under deaerated conditions (Figure 9d, blue), confirming molecular oxygen as the primary substrate for ^1^O_2_ generation. These results suggest that the ^1^O_2_ formation predominantly arose from a Type II photosensitization process that involves energy transfer from the chemiexcited iridium­(III) complex to surrounding molecular oxygen, rather than the dissociation of the intrinsic cyclic peroxide unit of **ADO**_**P**_. We also tested for other ROS such as superoxide anion radical (O_2_^•–^) and hydroxyl radical (HO^•^), using dihydrorhodamine 123 (DHR123) and hydroxyphenyl fluorescein (HPF) as the indicators, respectively. A notable time-dependent fluorescence increase was observed only in samples containing **ADO**_**P**_, complex **3a**, ALP, and DHR123 or HPF, but not in those lacking ALP (Figure 9e and f), indicating that in addition to the Type II mechanism, adduct **ADO**_**P**_ **–3a** also generated ROS via a Type I process under light-free conditions.

### CRET-Induced ROS Generation in Live Cells

Given the efficient ROS generation by adduct **ADO**_**P**_ **–3a** without external light excitation *in vitro*, we studied whether CRET-induced ROS production was also applicable in live cells. ALP is a ubiquitous membrane-bound glycoprotein and has served as an important cancer biomarker due to its overexpression in various tumor tissues,^75^ and thus, HeLa cells were selected as a model for their relatively high ALP expression. Intracellular ROS generation was monitored using the cell-permeable probe 2′,7′-dichlorodihydrofluorescein diacetate (DCFH-DA), which is deacetylated by intracellular esterase into nonfluorescent DCFH_2_ and subsequently oxidized by ROS to yield the highly green fluorescent product DCF. In our experiments, HeLa cells were treated with (1) blank medium, (2) probe **ADO**_**P**_ (500 μM) alone, (3) complex **3a** (5 μM) alone, or (4) adduct **ADO**_**P**_ **–3a** ([**ADO**_**P**_] = 500 μM, [**3a**] = 5 μM) in the dark for 4 h and stained with DCFH-DA (5 μM) for 30 min. No significant fluorescence was observed in cells treated with **ADO**_**P**_ or complex **3a** alone (Figures 10a and S12), indicating that neither of these components generated ROS on its own under light-free conditions. In contrast, strong green fluorescence of DCF was detected in cells treated with adduct **ADO**_**P**_ **–3a** (Figures 10a and S12), signifying ROS generation through CRET. Importantly, pretreatment of HeLa cells with 2,5-dimethoxy-*N*-(quinolin-3-yl)­benzenesulfonamide (DQB) (Figures 10b and S12), a highly selective inhibitor of tissue-nonspecific ALP,^76^ or phospholipase C (PLC) (Figures 10c and S12), an enzyme that cleaves glycosylphosphatidylinositol anchors and removes ALP from the cell membrane,^77^ led to a significant reduction in DCF fluorescence intensity. These results demonstrate that intracellular ROS generation by adduct **ADO**_**P**_ **–3a** was critically dependent on ALP activity, further validating ALP as the specific trigger for CRET-driven ROS generation. We also evaluated whether adduct **ADO**_**P**_ **–3a** generated ROS in HEK293 cells, which display lower ALP expression. Encouragingly, the cells exhibited negligible DCF signal when exposed to adduct **ADO**_**P**_ **–3a**, probe **ADO**_**P**_, or complex **3a** alone (Figures 10d and S12), confirming that adduct **ADO**_**P**_ **–3a** selectively generated ROS in cancer cells with elevated ALP levels.

**Figure 10:**
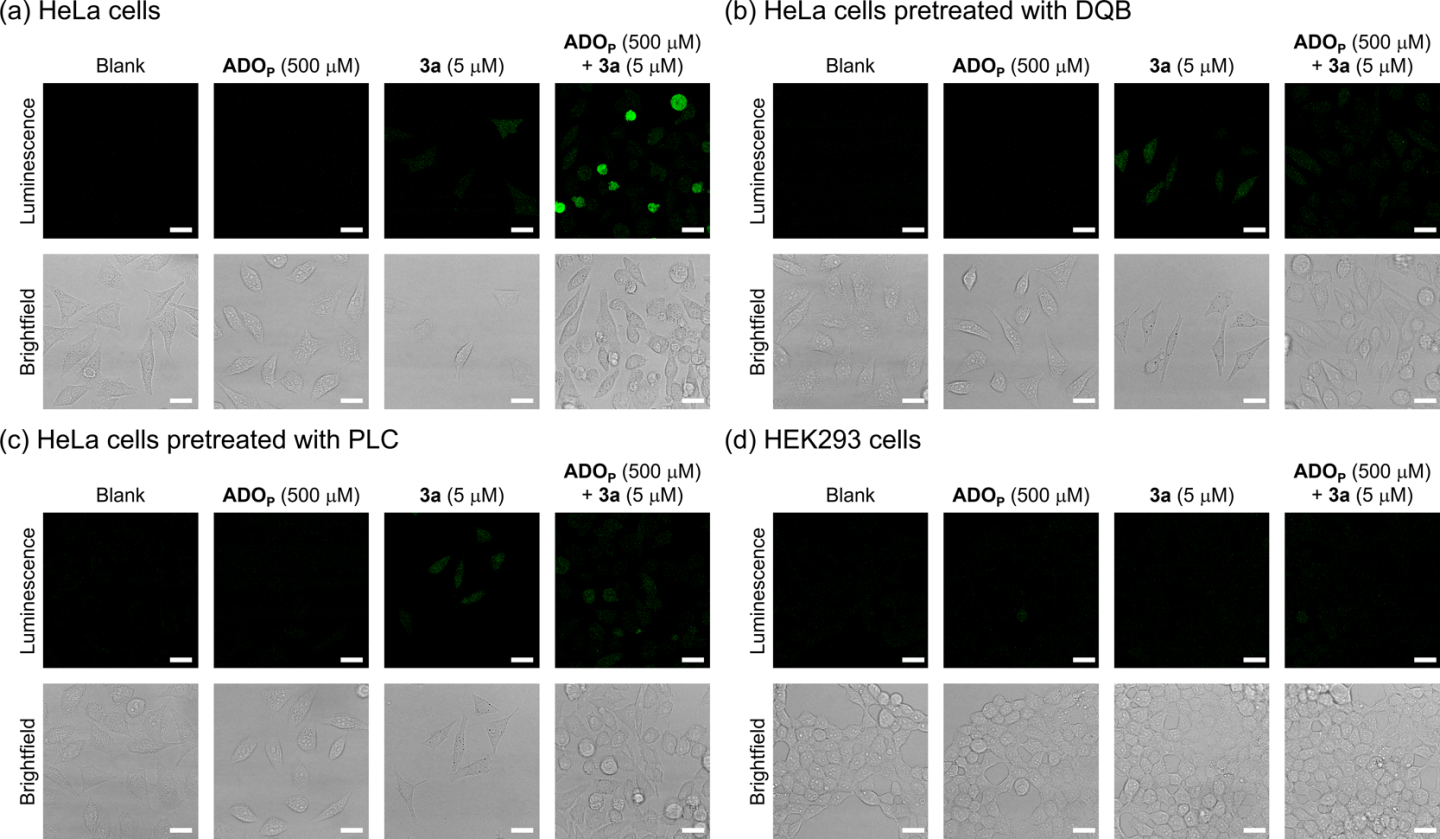
LSCM images of (a) HeLa cells without pretreatment, (b) HeLa cells pretreated with DQB (20 μM, 1 h), (c) HeLa cells pretreated with PLC (0.2 unit mL^–1^, 1 h), and (d) HEK293 cells, incubated without or with probe **ADO**_**P**_ (500 μM, 4 h), complex **3a** (5 μM, 4 h), or adduct **ADO**_**P**_ **–3a** ([**ADO**_**P**_] = 500 μM, [**3a**] = 5 μM, 4 h) in the dark, and stained with DCFH-DA (5 μM, 30 min; λ_ex_ = 488 nm, λ_em_ = 510–530 nm). Scale bar = 25 μm.

### “Dark” PDT in Cancer Cells

We hypothesized that adduct **ADO**_**P**_ **–3a** can act as an ALP-activatable, cancer-targeted CL-induced PDT agent. Thus, the cytotoxicity of probe **ADO**_**P**_, complex **3a**, and adduct **ADO**_**P**_ **–3a** was evaluated in cancerous HeLa cells (high ALP activity) and normal HEK293 cells (low ALP activity) using the MTT assay. Cells were incubated with **ADO**_**P**_ (0–800 μM) or adduct **ADO**_**P**_ **–3a** ([**ADO**_**P**_] = 0–800 μM, [**3a**] = 5 μM) for 4 h and then kept in the dark for an additional 20 h. Probe **ADO**_**P**_ was completely noncytotoxic in both cell lines (IC_50_ > 800 μM; Figure 11a). When cells were treated with complex **3a** alone, viability in both cell lines decreased to *ca*. 70%, reflecting the intrinsic dark cytotoxicity of complex **3a** (Figure 11b). Interestingly, in HeLa cells, co-incubation with complex **3a** (5 μM) and increasing concentrations of **ADO**_**P**_ (0–800 μM) markedly reduced the cell viability from *ca*. 65% ([**ADO**_**P**_] = 0 μM) to as low as 6% ([**ADO**_**P**_] = 800 μM) (Figure 11b). In contrast, HEK293 cell viability remained at *ca*. 70% under the same conditions (Figure 11b). These findings are consistent with our intracellular ROS detection experiments, which showed CL-induced ROS generation in HeLa cells but not in HEK293 cells (Figure 10), highlighting the potential of adduct **ADO**_**P**_ **–3a** for cancer-selective, light-free CL-induced PDT.

**Figure 11:**
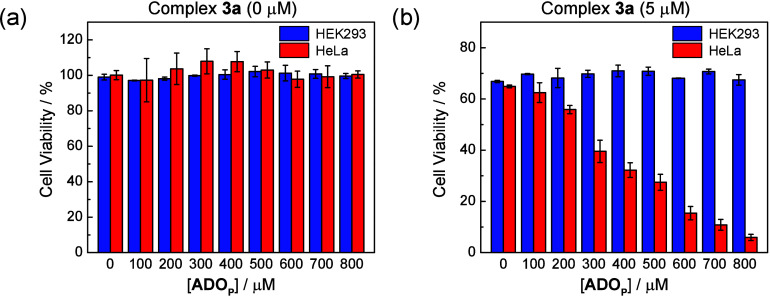
Viability of HEK293 (blue) and HeLa cells (red) exposed to varying concentrations of (a) probe **ADO**_**P**_ (0–800 μM) or (b) adduct **ADO**_**P**_ **–3a** ([**ADO**_**P**_] = 0–800 μM, [**3a**] = 5 μM) for 4 h and further incubated in the dark for an additional 20 h.

We further assessed the cell viability using the Calcein-AM/propidium iodide (PI) double staining assay. Calcein-AM stains viable cells with green fluorescence following esterase-mediated activation, whereas PI labels membrane-compromised (dead) cells with red fluorescence. HeLa cells treated with probe **ADO**_**P**_ (600 μM) or complex **3a** (5 μM) alone displayed strong Calcein-AM fluorescence and no PI signal (Figures 12 and S13), indicating high cell viability. In contrast, treatment with adduct **ADO**_**P**_ **–3a** ([**ADO**_**P**_] = 300 μM, [**3a**] = 5 μM) reduced Calcein-AM fluorescence and increased PI fluorescence. Increasing **ADO**_**P**_ concentration to 600 μM further decreased the cell viability, as evidenced by a pronounced increase in PI fluorescence. Extensive cell death was also observed when complex **3a**-treated cells were subjected to external light irradiation (Figures S13 and S14).

**Figure 12:**
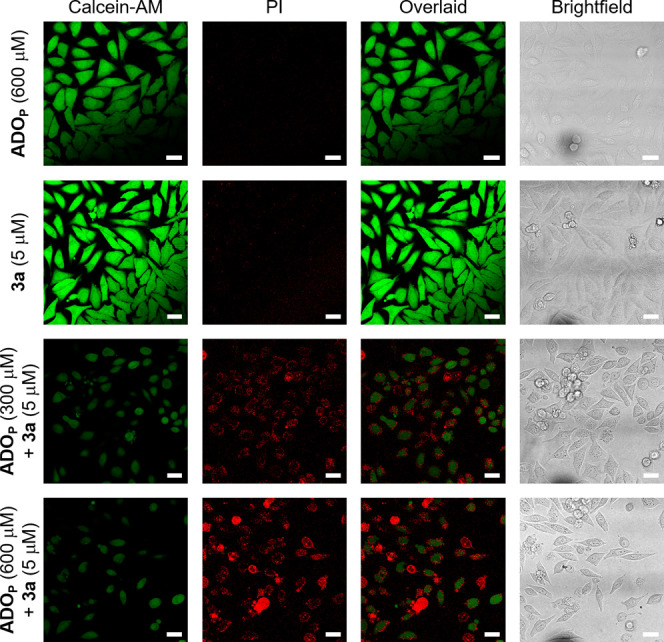
LSCM images of HeLa cells incubated with probe **ADO**_**P**_ (600 μM, 4 h), complex **3a** (5 μM, 4 h), or adduct **ADO**_**P**_ **–3a** ([**ADO**_**P**_] = 300 or 600 μM, [**3a**] = 5 μM, 4 h) in the dark, and stained with Calcein-AM (1 μM, 30 min; λ_ex_ = 488 nm, λ_em_ = 510–540 nm) and PI (10 μM, 30 min; λ_ex_ = 532 nm, λ_em_ = 610–640 nm). Scale bar = 25 μm.

Compared to conventional 2D monolayer cell cultures, 3D multicellular tumor spheroids (MCTSs) closely replicate key features of solid tumors, including extracellular matrix (ECM) deposition, cell–cell and cell–ECM interactions, and pathophysiologically relevant gradients of pH, oxygen, and nutrients.^78,79^ Large MCTSs develop a distinct structural organization with spatial heterogeneity, comprising a proliferative outer layer, a quiescent inner layer, and a hypoxic–necrotic core. This structure mimics the diffusion barriers and microenvironment-driven drug resistance observed *in vivo*, making MCTSs an effective model for assessing CL-induced PDT within a more realistic tumor-like architecture, especially in areas with limited light penetration and restricted oxygen availability. Thus, we established 3D HeLa MCTSs (with a diameter of *ca*. 400 μm) to evaluate the CL-induced PDT activity of adduct **ADO**_**P**_ **–3a**. As revealed by the Calcein-AM/PI assay, HeLa MCTSs treated with probe **ADO**_**P**_ (800 μM) or complex **3a** (5 μM) alone exhibited intense green fluorescence from Calcein-AM and minimal red fluorescence from PI (Figure 13). These signals were comparable to the untreated control, showing that cells within the MCTSs remained highly viable under these conditions. However, treatment of the MCTSs with adduct **ADO**_**P**_ **–3a** ([**ADO**_**P**_] = 800 μM, [**3a**] = 5 μM) significantly diminished Calcein-AM fluorescence and markedly increased PI fluorescence, indicating extensive cell death induced by the adduct. These findings demonstrate the potent CL-induced PDT activity of adduct **ADO**_**P**_ **–3a** toward HeLa cells and MCTSs.

**Figure 13:**
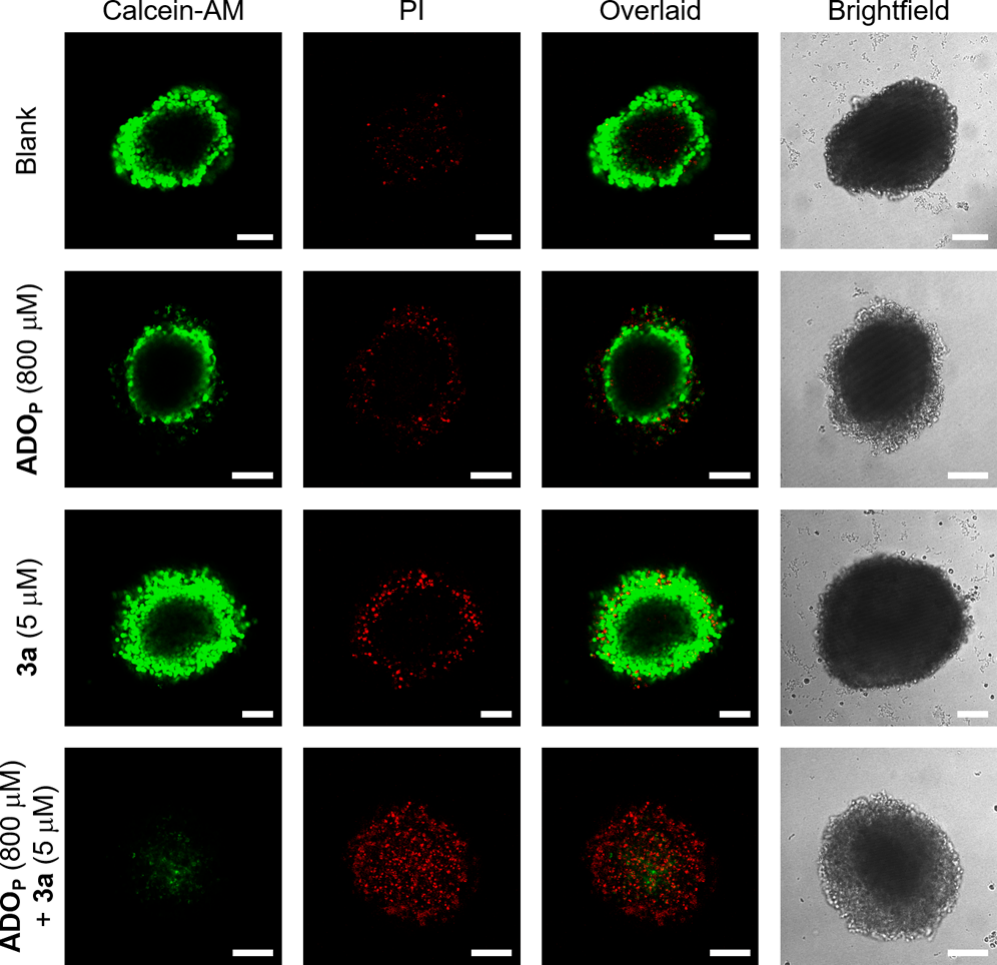
LSCM images of HeLa MCTSs incubated without or with probe **ADO**_**P**_ (800 μM, 4 h), complex **3a** (5 μM, 4 h), or adduct **ADO**_**P**_ **–3a** ([**ADO**_**P**_] = 800 μM, [**3a**] = 5 μM, 4 h) in the dark, and stained with Calcein-AM (1 μM, 30 min; λ_ex_ = 488 nm, λ_em_ = 510–540 nm) and PI (10 μM, 30 min; λ_ex_ = 532 nm, λ_em_ = 610–640 nm). Scale bar = 100 μm.

Since many iridium­(III)-based PSs induce apoptosis in cancer cells upon light irradiation,^65,66,80^ we investigated whether adduct **ADO**_**P**_ **–3a** also triggered apoptosis under light-free conditions. Annexin V staining was used to detect early apoptotic cells by binding phosphatidylserine exposed on the outer leaflet of the plasma membrane. Treatment with probe **ADO**_**P**_ (600 μM) or complex **3a** (5 μM) alone in the dark produced negligible Annexin V signal in HeLa cells (Figures 14 and S15), indicating no apoptosis induction under these conditions. In contrast, cells treated with adduct **ADO**_**P**_ **–3a** ([**ADO**_**P**_] = 600 μM, [**3a**] = 5 μM) showed clear membrane-associated Annexin V fluorescence. Similar Annexin V staining was also observed in complex **3a**-treated cells exposed to light irradiation (Figures S15 and S16). These results demonstrate that adduct **ADO**_**P**_ **–3a** induced apoptosis in the absence of external light excitation, supporting its application in CL-induced PDT.

**Figure 14:**
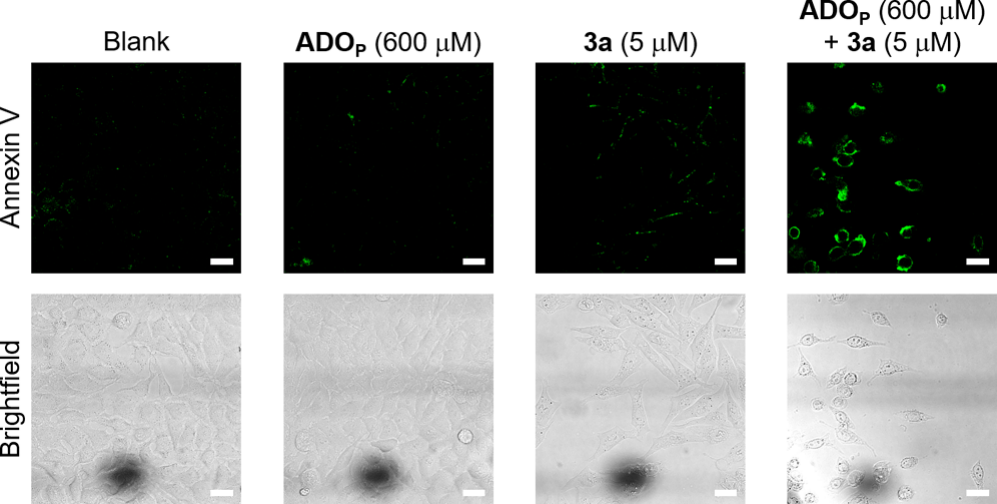
LSCM images of HeLa cells incubated without or with probe **ADO**_**P**_ (600 μM, 4 h), complex **3a** (5 μM, 4 h), or adduct **ADO**_**P**_ **–3a** ([**ADO**_**P**_] = 600 μM, [**3a**] = 5 μM, 4 h) in the dark, and stained with Alexa Fluor 647–Annexin V conjugate (5 μL, 15 min; λ_ex_ = 633 nm, λ_em_ = 650–680 nm). Scale bar = 25 μm.

## Conclusion

CL-induced PDT offers an efficient, light-free strategy to selectively activate iridium­(III)-based PSs in cancer cells. In this study, we designed three luminescent cyclometalated iridium­(III) polypyridine complexes bearing a TMCD moiety and utilized three enzyme-responsive, chemiluminescent spiroadamantyl phenoxy-1,2-dioxetane probes as internal light sources. The TMCD functionalization endowed the complexes with host–guest recognition capabilities, enhanced water solubility, and reduced dark cytotoxicity, while preserving high ^1^O_2_ generation efficiencies. Supramolecular complexation between spiroadamantyl phenoxy-1,2-dioxetane probes and TMCD-modified iridium­(III) complexes yielded stable adducts; upon enzyme activation, these assemblies displayed efficient CRET from the dioxetane probes to the iridium­(III) acceptors. Among the combinations tested, probe **ADO**_**P**_ and complex **3a** (adduct **ADO**_**P**_ **–3a**) were selected for further evaluation. Following ALP activation, adduct **ADO**_**P**_ **–3a** exhibited efficient CRET, red-shifted CL, robust ROS generation under light-free conditions, and successful activation in both buffer solutions and live cells, highlighting its potential for simultaneous imaging and therapy. Importantly, adduct **ADO**_**P**_ **–3a** selectively generated ROS and elicited potent cytotoxicity in cancerous HeLa cells with high ALP expression under light-free conditions, while remaining noncytotoxic in normal HEK293 cells. Additionally, the adduct induced apoptosis in HeLa cells without external light irradiation, paralleling the apoptotic response observed upon photoactivation of complex **3a**. Furthermore, adduct **ADO**_**P**_ **–3a** retained high CL-induced PDT activity in 3D HeLa MCTSs. While MCTSs recapitulate tumor hypoxia, they lack vasculature and dynamic fluid flow, which are critical for nutrient exchange, drug delivery, and clearance *in vivo*. Thus, *in vivo* studies will be the next step to address the remaining gaps and further substantiate the translational relevance of our findings.

In conclusion, our findings establish supramolecular assemblies of spiroadamantyl phenoxy-1,2-dioxetanes and iridium­(III) TMCD complexes as cancer-selective, light-free CL-induced PDT agents and highlight a supramolecular approach that integrates enzyme responsiveness, targeted activation, and efficient ROS generation. Crucially, the modularity of the adamantane-bearing guest allows this system to function as a versatile “plug-and-play” platform; by simply derivatizing phenoxy-1,2-dioxetanes with different recognition moieties, a broad spectrum of cancer-associated enzymes can be targeted. This platform opens a path toward theranostic applications and motivates further optimization and *in vivo* evaluation for precision oncology.

## Supplementary Material



## Notes

Synthetic procedures, characterization data, UV–vis absorption spectra, emission spectra, photophysical data, chemiluminescence spectra and kinetic profiles, and laser-scanning confocal images (PDF)
