# Supplementary material for: Enzyme-Responsive Chemiluminescent Probes Assembled with Iridium(III) Photosensitizers via Host–Guest Chemistry for Chemiluminescence-Induced Photodynamic Therapy
Source: J Am Chem Soc. 2026 Jul 1;148(27):28238–53. doi: 10.1021/jacs.6c01728 (PMC13383634; doi:10.1021/jacs.6c01728)
Supplement: Supplementary file 1 [file ja6c01728_si_001.pdf]

Supporting Information

Enzyme-Responsive Chemiluminescent Probes Assembled with Iridium(III) Photosensitizers via Host–Guest Chemistry for Chemiluminescence-Induced Photodynamic Therapy

Jia-Hao Wang,[†] Lawrence Cho-Cheung Lee,[†] Alex Man-Hei Yip,[†,‡] Justin Shum,[†,‡] Qi-Hang Cheng,[†] Eunice Chiu-Lam Mak,[†,Φ] and Kenneth Kam-Wing Lo*,[†,Φ]

[†]Department of Chemistry, City University of Hong Kong, Tat Chee Avenue, Hong Kong, P. R. China; Email: bhkenlo@cityu.edu.hk

[‡]Laboratory for Synthetic Chemistry and Chemical Biology Limited, Units 1503–1511, 15/F, Building 17 W, Hong Kong Science Park, New Territories, Hong Kong, P. R. China

[Φ]State Key Laboratory of Terahertz and Millimeter Waves, City University of Hong Kong, Tat Chee Avenue, Hong Kong, P. R. China

Experimental **S9**

Table S1 Electronic absorption spectral data of the iridium(III) complexes at 298 K. **S41**

Table S2 CL maxima (λ_{max}), quantum yields (Φ_{CL}), and half-lives ($t_{1/2}$) of probe **ADO$_P$** (10 μM), adduct **ADO$_P$**–TMCD ([**ADO$_P$**] = 10 μM, [TMCD] = 100 μM), and adducts **ADO$_P$–1a**, **ADO$_P$–2a**, and **ADO$_P$–3a** ([**ADO$_P$**] = 10 μM, [Ir] = 100 μM) in Tris buffer (50 mM, pH 9.0) at 298 K in the presence of ALP (1 unit mol^{-1}). **S43**

Figure S1 Electronic absorption spectra of complexes **1a** – **3a** in CH_2Cl_2 (black), CH_3CN (red), and H_2O (green) at 298 K. **S44**

Figure S2 Electronic absorption spectra of complexes **1b** – **3b** in CH_2Cl_2 (black) and CH_3CN (red) at 298 K. **S45**

Figure S3 Normalized emission spectra of complexes **1a** – **3a** in CH_2Cl_2 (black), CH_3CN (red), and H_2O (green) at 298 K and alcohol glass (blue) at 77 K. **S46**

Figure S4 Normalized emission spectra of complexes **1b** – **3b** in CH_2Cl_2 (black), CH_3CN (red), and H_2O/MeOH (7:3, *v*/*v*) (green) at 298 K and alcohol glass (blue) at 77 K. **S47**

Figure S5 ^{31}P NMR spectra of probe **ADO$_P$** (4 mM) in D_2O at 298 K in the absence and presence of complex **1a** (0.5, 1, 2, and 4 mM). **S48**

Figure S6 Benesi–Hildebrand plots for the supramolecular host–guest interaction of (a) complex **1a**, (b) complex **2a**, and (c) complex **3a** with **ADO$_P$** in D_2O at 298 K. **S49**

Figure S7 CL spectra of probe **ADO$_P$** (100 μM) (blue), adduct **ADO$_P$–1a** (black), adduct **ADO$_P$–2a** (orange), and adduct **ADO$_P$–3a** (red) ([**ADO$_P$**] = 100 μM, [Ir] = 100 μM) in Tris buffer (50 mM, pH 9.0) in the absence of ALP. **S50**

Figure S8 CL kinetic profiles of probe **ADO$_P$** (10 μM) (blue), adduct **ADO$_P$**–TMCD ([**ADO$_P$**] = 10 μM, [TMCD] = 100 μM) (green), adduct **ADO$_P$–1a** (black), adduct **ADO$_P$–2a** (orange), and adduct **ADO$_P$–3a** (red) ([**ADO$_P$**] = 10 μM, [Ir] = 100 μM) in Tris buffer (50 mM, pH 9.0) at 298 K in the presence of (a) ALP (1 unit mL^{-1}), (b) PIC3 (1:10 solution)-pretreated ALP (1 unit mL^{-1}), or (c) PIC3 (1:10 solution). Insets: Enlarged view of the CL kinetic profiles for probe **ADO$_P$** (blue), adduct **ADO$_P$**–TMCD (green), adduct **ADO$_P$–1a** (black), adduct **ADO$_P$–2a** (orange), and adduct **ADO$_P$–3a** (red) under the respective treatments. (d) Total photon counts for probe **ADO$_P$** (10 μM), adduct **ADO$_G$**–TMCD ([**ADO$_P$**] = 10 μM, [TMCD] = 100 μM), adduct **ADO$_P$–1a**, adduct **ADO$_P$–2a**, and adduct **ADO$_P$–3a** ([**ADO$_G$**] = 10 μM, [Ir] = 100 μM) in Tris buffer (50 mM, pH 9.0) at 298 K in the presence of ALP **S51**

(1 unit mL^{-1}) (red), PIC3 (1:10 solution)-pretreated ALP (1 unit mL^{-1}) (blue), or (c) PIC3 (1:10 solution) (black).

Figure S9 CL kinetic profiles of probe **ADO$_E$** (10 μM) (blue), adduct **ADO$_E$**–TMCD ([**ADO$_E$**] = 10 μM, [TMCD] = 100 μM) (green), adduct **ADO$_E$–1a** (black), adduct **ADO$_E$–2a** (orange), and adduct **ADO$_E$–3a** (red) ([**ADO$_E$**] = 10 μM, [Ir] = 100 μM) in PBS (1X, pH 7.4) at 298 K in the presence of (a) PLE (1 unit mL^{-1}), (b) BNPP (10 mM)-pretreated PLE (1 unit mL^{-1}), or (c) BNPP (10 mM). Insets: Enlarged view of the CL kinetic profiles for probe **ADO$_E$** (blue), adduct **ADO$_E$**–TMCD (green), adduct **ADO$_E$–1a** (black), adduct **ADO$_E$–2a** (orange), and adduct **ADO$_E$–3a** (red) under the respective treatments. (d) Total photon counts for probe **ADO$_E$** (10 μM), adduct **ADO$_E$**–TMCD ([**ADO$_E$**] = 10 μM, [TMCD] = 100 μM), adduct **ADO$_E$–1a**, adduct **ADO$_E$–2a**, and adduct **ADO$_E$–3a** ([**ADO$_E$**] = 10 μM, [Ir] = 100 μM) in PBS (1X, pH 7.4) at 298 K in the presence of PLE (1 unit mL^{-1}) (red), BNPP (10 mM)-pretreated PLE (1 unit mL^{-1}) (blue), or (c) BNPP (10 mM) (black). **S52**

Figure S10 CL kinetic profiles of probe **ADO$_G$** (10 μM) (blue), adduct **ADO$_G$**–TMCD ([**ADO$_G$**] = 10 μM, [TMCD] = 100 μM) (green), adduct **ADO$_G$–1a** (black), adduct **ADO$_G$–2a** (orange), and adduct **ADO$_G$–3a** (red) ([**ADO$_G$**] = 10 μM, [Ir] = 100 μM) in PBS (1X, pH 7.4) at 298 K in the presence of (a) β-gal (1 **S53**

unit mL^{-1}), (b) EDTA (20 mM)-pretreated β-gal (1 unit mL^{-1}), or (c) EDTA (20 mM). Insets: Enlarged view of the CL kinetic profiles for probe **ADO$_G$** (blue), adduct **ADO$_G$**–TMCD (green), adduct **ADO$_G$–1a** (black), adduct **ADO$_G$–2a** (orange), and adduct **ADO$_G$–3a** (red) under the respective treatments. (d) Total photon counts for probe **ADO$_G$** (10 μM), adduct **ADO$_G$**–TMCD ([**ADO$_G$**] = 10 μM, [TMCD] = 100 μM), adduct **ADO$_G$–1a**, adduct **ADO$_G$–2a**, and adduct **ADO$_G$–3a** ([**ADO$_G$**] = 10 μM, [Ir] = 100 μM) in PBS (1X, pH 7.4) at 298 K in the presence of β-gal (1 unit mL^{-1}) (red), EDTA (20 mM)-pretreated β-gal (1 unit mL^{-1}) (blue), or (c) EDTA (20 mM) (black).

Figure S11 CL kinetic profiles of (a) adduct **ADO$_E$**–TMCD ([**ADO$_E$**] = 10 μM, [TMCD] = 100 μM), (b) adduct **ADO$_E$–1a**, (c) adduct **ADO$_E$–2a**, and (d) adduct **ADO$_E$–3a** ([**ADO$_E$**] = 10 μM, [Ir] = 100 μM) in PBS (1X, pH 7.4) in the presence of PLE (1 unit mL^{-1}) without (red) or with (black) the addition of AD (1 mM). **S54**

Figure S12 Corrected cell fluorescence of HeLa cells without pretreatment, HeLa cells pretreated with DQB (20 μM, 1 h), HeLa cells pretreated with PLC (0.2 unit mL^{-1}, 1 h), and HEK293 cells, incubated with blank medium (4 h; black), probe **ADO$_P$** (500 μM, 4 h; green), complex **3a** (5 μM, 4 h; blue), or adduct **ADO$_P$–3a** ([**ADO$_P$**] = 500 μM, [**3a**] = 5 μM, 4 h; red), and stained with **S55**

DCFH-DA (5 μM, 30 min; λ_{ex} = 488 nm, λ_{em} = 510 – 530 nm). Error bars represent standard deviations of three independent replicates.

Figure S13 Corrected cell fluorescence of HeLa cells incubated with probe **ADO$_P$** (600 μM, 4 h), complex **3a** (5 μM, 4 h), adduct **ADO$_P$–3a** ([**ADO$_P$**] = 300 or 600 μM, [**3a**] = 5 μM, 4 h), or complex **3a** (5 μM, 4 h) followed by light irradiation (450 nm, 15.5 mW cm^{-2}, 10 min), and stained with Calcein-AM (1 μM, 30 min; λ_{ex} = 488 nm, λ_{em} = 510 – 540 nm; green) and PI (10 μM, 30 min; λ_{ex} = 532 nm, λ_{em} = 610 – 640 nm; red). Error bars represent standard deviations of three independent replicates. **S56**

Figure S14 LSCM images of HeLa cells incubated with complex **3a** (5 μM, 4 h) followed by light irradiation (450 nm, 15.5 mW cm^{-2}, 10 min), and stained with Calcein-AM (1 μM, 30 min; λ_{ex} = 488 nm, λ_{em} = 510 – 540 nm) and PI (10 μM, 30 min; λ_{ex} = 532 nm, λ_{em} = 610 – 640 nm). Scale bar = 25 μm. **S57**

Figure S15 Corrected cell fluorescence of HeLa cells incubated with blank medium (4 h), probe **ADO$_P$** (600 μM, 4 h), complex **3a** (5 μM, 4 h), adduct **ADO$_P$–3a** ([**ADO$_P$**] = 600 μM, [**3a**] = 5 μM, 4 h), or complex **3a** (5 μM, 4 h) followed by light irradiation (450 nm, 15.5 mW cm^{-2}, 10 min), and stained with Alexa Fluor 647–Annexin V conjugate (5 μL, 15 min; λ_{ex} = 633 nm, λ_{em} = 650 – **S58**

	680 nm). Error bars represent standard deviations of three independent replicates.	
**Figure S16**	LSCM images of HeLa cells incubated with complex **3a** (5 μM, 4 h) followed by light irradiation (450 nm, 15.5 mW cm^{-2}, 10 min), and stained with Alexa Fluor 647–Annexin V conjugate (5 μL, 15 min; λ_{ex} = 633 nm, λ_{em} = 650 – 680 nm). Scale bar = 25 μm.	**S59**
**Figure S17**	ESI mass spectrum of the ligand bpy-TMCD in MeOH.	**S60**
**Figure S18**	ESI mass spectra of complexes **1a** – **3a** in MeOH.	**S61**
**Figure S19**	HR-ESI mass spectra of complexes **1a** – **3a** in MeOH.	**S62**
**Figure S20**	MALDI-TOF mass spectra of complexes **1a** – **3a**.	**S63**
**Figure S21**	ESI mass spectra of complexes **1b** – **3b** in MeOH.	**S64**
**Figure S22**	HR-ESI mass spectra of complexes **1b** – **3b** in MeOH.	**S65**
**Figure S23**	^{1}H NMR spectrum of the ligand bpy-TMCD in CD_3CN at 298 K.	**S66**
**Figure S24**	^{1}H NMR spectrum of complex **1a** in $CDCl_3$ at 298 K.	**S67**
**Figure S25**	^{13}C NMR spectrum of complex **1a** in $CDCl_3$ at 298 K.	**S68**
**Figure S26**	^{1}H NMR spectrum of complex **2a** in $CDCl_3$ at 298 K.	**S69**
**Figure S27**	^{13}C NMR spectrum of complex **2a** in $CDCl_3$ at 298 K.	**S70**
**Figure S28**	^{1}H NMR spectrum of complex **3a** in $CDCl_3$ at 298 K.	**S71**
**Figure S29**	^{13}C NMR spectrum of complex **3a** in $CDCl_3$ at 298 K.	**S72**

**Figure S30**	^{1}H NMR spectrum of complex **1b** in $CDCl_3$ at 298 K.	**S73**
**Figure S31**	^{13}C NMR spectrum of complex **1b** in $CDCl_3$ at 298 K.	**S74**
**Figure S32**	^{1}H NMR spectrum of complex **2b** in $CDCl_3$ at 298 K.	**S75**
**Figure S33**	^{13}C NMR spectrum of complex **2b** in $CDCl_3$ at 298 K.	**S76**
**Figure S34**	^{1}H NMR spectrum of complex **3b** in $CDCl_3$ at 298 K.	**S77**
**Figure S35**	^{13}C NMR spectrum of complex **3b** in $CDCl_3$ at 298 K.	**S78**
**Figure S36**	^{1}H NMR spectrum of the intermediate **ADE_E** in CD_3OD at 298 K.	**S79**
**Figure S37**	^{1}H NMR spectrum of probe **ADO_E** in CD_3OD at 298 K.	**S80**
**Appendix**	Synthesis, Characterization, and Properties of $[Rh(bsn)_2(bpy\text{-}TMCD)](Cl)$ (**3c**)	**S81**
**References**		**S92**

EXPERIMENTAL

Materials and Reagents

All solvents were of analytical reagent grade and purified according to standard procedures.[1] Iodomethane, triphenylphosphine, sodium hydride, 4-nitrophenyl chloroformate, pyridine, acetic anhydride, methylene blue, and 1-adamantanemethylamine (AD) were purchased from Acros. 4,4'-Dimethyl-2,2'-bipyridine, selenium dioxide, sodium metabisulfite, iridium(III) chloride trihydrate, 2-phenylpridine (Hppy), 2-phenylquinoline (Hpq), Tris, alkaline phosphatase (ALP, from calf intestine), porcine liver esterase (PLE), β-galactosidase (β-gal, from *Escherichia coli*), ethylenediaminetetraacetic acid (EDTA), hydrogen peroxide (H_2O_2), 2,5-dimethoxy-*N*-(quinolin-3-yl)benzenesulfonamide (DQB), and phospholipase C (PLC) were purchased from Sigma-Aldrich. Sodium chloride, triethylamine, anhydrous magnesium sulfate, luminol, and bis(4-nitrophenyl) phosphate (BNPP) were purchased from Energy Chemical. α-Cyano-4-hydroxycinnamic acid (CCA), trimethyl-β-cyclodextrin (TMCD), 3-(4,5-dimethylthiazol-2-yl)-2,5-diphenyltetrazolium bromide (MTT), and 2',7'-dichlorodihydrofluorescein ($DCFH_2$) were purchased from Macklin. 3-(2'-Spiroadamantane)-4-methoxy-4-(3''-phosphoryloxy)phenyl-1,2-dioxetane (**ADO$_\mathbf{P}$**) and 2',7'-dichlorodihydrofluorescein diacetate (DCFH-DA) were purchased from Aladdin. Phosphatase inhibitor cocktail III (PIC3) was purchased from Calbiochem. All these chemicals were used without further purification. 2-(1-Naphthyl)benzothiazole (Hbsn),[2] 4-carboxaldehyde-4'-

methyl-2,2'-bipyridine (bpy-CHO),[3] 4-(4-nitrophenyloxycarbonyloxymethyl)-4'-methyl-2,2'-bipyridine (bpy-NPC),[4] permethylated 6-monoamino-6-monodeoxy-β-cyclodextrin (TMCD-NH_2),[5] 4-(*N*-*n*-butylaminocarbonyloxymethyl)-4'-methyl-2,2'-bipyridine (bpy-C4),[6] iridium(III) dimers $[Ir_2(N^\wedge C)_4Cl_2]$ (HN^C = Hppy, Hpq, and Hbsn),[7] 3-(((1*r*,3*r*,5*R*,7*S*)-adamantan-2-ylidene)(methoxy)methyl)-2-chlorophenol (**ADE$_{OH}$**),[8] and (2*R*,3*S*,6*S*)-2-(2-chloro-3-((1*R*,3*R*,5*R*,7*R*)-4'-methoxyspiro[adamantane-2,3'-[1,2]dioxetan]-4'-yl)phenoxy)-6-(hydroxymethyl)tetrahydro-2*H*-pyran-3,4,5-triol (**ADO$_G$**)[8] were prepared according to literature procedures. All buffer components were of biological grade and used as received. Autoclaved Milli-Q water was used for the preparation of the aqueous solutions. Dulbecco's modified Eagle's medium (DMEM), fetal bovine serum (FBS), phosphate-buffered saline (PBS, 10X) at pH 7.4, trypsin-EDTA, penicillin/streptomycin, MitoTracker Deep Red, singlet oxygen sensor green (SOSG), Calcein-AM, propidium iodide (PI), Alexa Fluor 647–Annexin V conjugate, and Annexin V binding buffer were purchased from Invitrogen. Dihydrorhodamine 123 (DHR123) and hydroxyphenyl fluorescein (HPF) were purchased from MedChemExpress. HeLa and HEK293 cells were obtained from American Type Culture Collection.

Synthesis and Characterization

4-(*N*-Trimethyl-β-cyclodextrin-mono-6-deoxy-6-aminocarbonyloxymethyl)-4'-methyl-2,2'-bipyridine (bpy-TMCD)

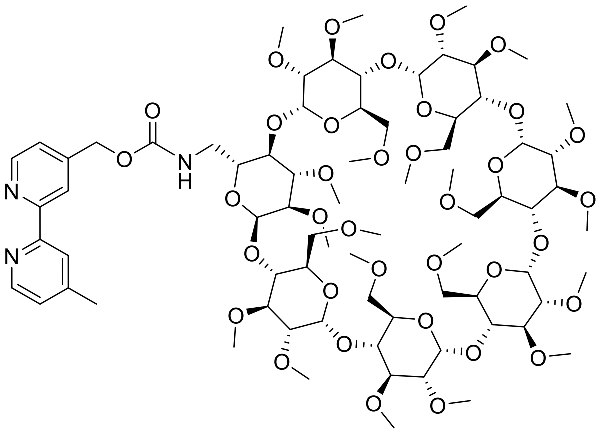

A mixture of bpy-NPC (155 mg, 0.42 mmol), TMCD-NH_2 (500 mg, 0.35 mmol), and triethylamine (118 μL, 0.84 mmol) in CH_2Cl_2 (20 mL) was stirred at room temperature under an inert atmosphere of N_2 for 18 h. Then, the solution was extracted with H_2O (20 mL). The aqueous phase was collected and further extracted with CH_2Cl_2 (30 mL × 2). The combined organic phase was dried over anhydrous $MgSO_4$, filtered, and evaporated to dryness under reduced pressure. The residual colorless oil was purified by column chromatography on silica gel using CH_2Cl_2/MeOH (20:1, *v*/*v*) as the eluent. The solvent was removed under reduced pressure to afford the product as a colorless oil. Yield: 366 mg (63%). ^{1}H NMR (400 MHz, CD_3CN, 298 K): δ 8.67 (d, J = 4.8 Hz, 1H, H6 of bpy), 8.56 (d, J = 5.2 Hz, 1H, H6' of bpy), 8.45 (s, 1H, H3 of bpy), 8.30 (s, 1H, H3' of bpy), 7.36 (d, J = 4.4 Hz, 1H, H5 of bpy), 7.24 (d, J = 4.8 Hz, 1H, H5' of bpy), 6.04 (s, 1H, NH), 5.29 – 4.89 (m, 9H, CH_2 of bpy, O_2C*H*CH of

TMCD), 3.94 – 2.93 (m, 102H, OC*H*(CH_2)(CH), OC*H*CH_2, NHCH_2, OCH_2, and OCH_3 of TMCD), 2.47 (s, 3H, CH_3 of bpy). ESI-MS (positive-ion mode) *m*/*z* found: 1641.1 $[M + H]^+$ calcd: 1639.77.

[Ir(ppy)$_2$(bpy-TMCD)](Cl) (**1a**)

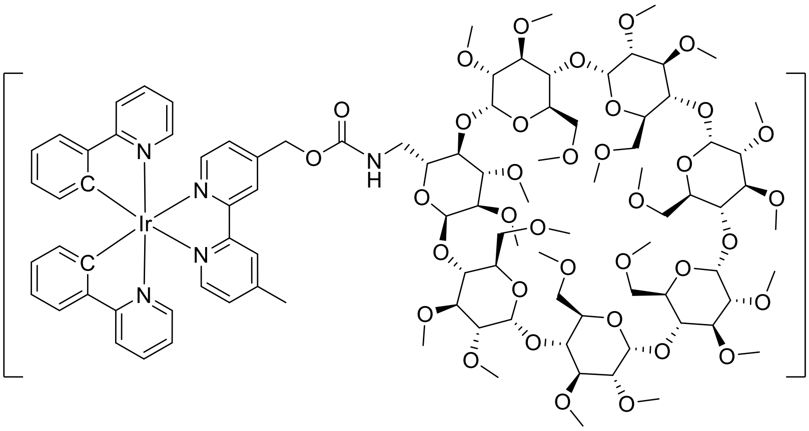

(Cl)

A mixture of [$Ir_2(ppy)_4Cl_2$] (43 mg, 0.04 mmol) and bpy-TMCD (100 mg, 0.06 mmol) in CH_2Cl_2/MeOH (20 mL) (1:1, *v*/*v*) was stirred at 298 K under an inert atmosphere of N_2 in the dark for 18 h. The solvent was removed under reduced pressure and the residual yellow solid was purified by column chromatography on silica gel using CH_2Cl_2/MeOH (15:1, *v*/*v*) as the eluent. The solvent was removed under reduced pressure to afford complex **1a** as a yellow solid. Yield: 114 mg (86%). ^{1}H NMR (300 MHz, $CDCl_3$, 298 K, TMS): δ 9.90 (s, 1H, H3 of bpy), 9.72 (s, 1H, H3' of bpy), 7.92 (d, J = 8.1 Hz, 2H, H6 and H6' of bpy), 7.83 – 7.72 (m, 3H, H5 of bpy and H3 of pyridyl ring of ppy), 7.71 – 7.63 (m, 3H, H5' of bpy and H3 of phenyl ring of ppy), 7.49 (d, J = 5.7 Hz, 2H, H4 of pyridyl ring of ppy), 7.39 (d, J = 5.4 Hz, 1H, NH), 7.25 – 7.12 (m, 2H, H6 of pyridyl ring of ppy), 7.09 – 6.97 (m, 4H, H5 of pyridyl ring and H4 of phenyl ring of ppy), 6.91 (t, J = 7.5 Hz, 2H, H5 of phenyl ring of ppy), 6.31 (d, J = 7.8 Hz, 2H, H6 of phenyl ring of ppy), 5.52 – 5.29 (m, 3H, CH_2 of bpy and ($NHCH_2CHO$)OC*H*CH of TMCD), 5.20 – 5.05 (m, 6H, O_2C*H*CH of TMCD), 3.99 – 3.10 (m, 102H, OC*H*(CH_2)(CH), OC*H*CH_2, NHC*H*$_2$, OCH_2, and OCH_3 of TMCD), 2.72 (s, 3H, CH_3 on bpy). ^{13}C NMR (150

MHz, $CDCl_3$, 298 K, TMS): δ 168.00, 167.95, 156.77, 156.59, 155.48, 153.23, 151.49, 150.87, 150.80, 149.13, 148.91, 148.43, 143.48, 143.44, 137.93, 137.88, 131.77, 131.74, 130.79, 130.74, 128.77, 128.53, 124.74, 124.50, 124.24, 123.13, 122.52, 122.46, 119.52, 99.02, 98.99, 98.94, 98.79, 98.67, 82.11, 82.03, 81.88, 81.86, 81.79, 81.59, 81.52, 81.11, 80.31, 80.21, 80.16, 80.02, 79.92, 79.66, 71.40, 71.35, 71.31, 71.19, 71.13, 71.03, 70.93, 70.85, 70.42, 63.42, 61.50, 61.47, 61.44, 61.20, 59.33, 59.07, 59.03, 58.99, 58.61, 58.53, 58.50, 58.43, 41.69, 21.40. IR (KBr) $\tilde{\nu}$/cm^{-1}: 3526 (N–H), 2972 (C–H), 1648 (C=O), 1034 (C–O–C). MALDI-TOF-MS (CCA) *m/z* found: 2144.3 [M – Cl$^-$]$^+$ calcd: 2141.4. HR-MS (ESI, positive mode) *m/z* found: 2140.8647 [M – Cl$^-$]$^+$ calcd for $IrC_{97}H_{137}N_5O_{36}$ 2140.8672.

[Ir(pq)$_2$(bpy-TMCD)](Cl) (**2a**)

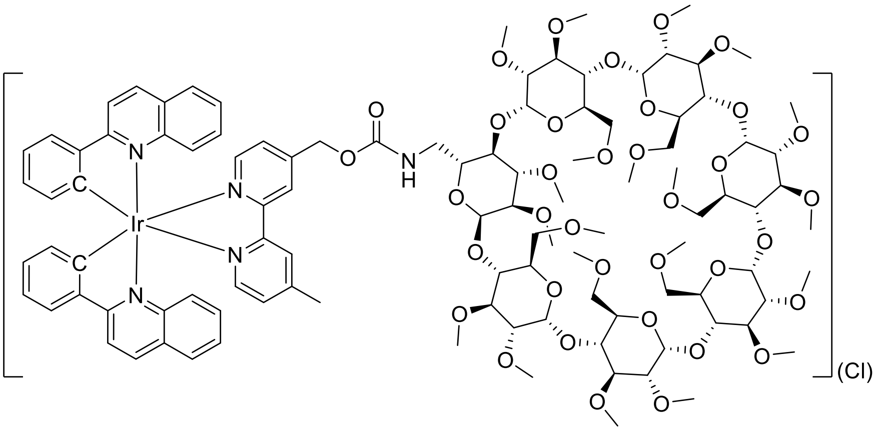

The synthetic procedure was similar to that of complex **1a**, except that [Ir$_2$(pq)$_4$Cl$_2$] (51 mg, 0.04 mmol) was used instead of [Ir$_2$(ppy)$_4$Cl$_2$]. The solvent was removed under reduced pressure to afford complex **2a** as an orange solid. Yield: 104 mg (75%). ^{1}H NMR (300 MHz, CDCl$_3$, 298 K, TMS): δ 9.58 (s, 1H, H3 of bpy), 9.31 (s, 1H, H3' of bpy), 8.29 – 8.10 (m, 4H, H6, and H6' of bpy and H4 of quinolinyl ring of pq), 8.04 – 7.92 (m, 3H, H5 of bpy and H3 of quinolinyl ring of pq), 7.87 (d, *J* = 5.7 Hz, 1H, H5' of bpy), 7.72 (d, *J* = 8.1 Hz, 2H, H3 of phenyl ring of pq), 7.68 – 7.52 (m, 1H, NH), 7.39 (t, *J* = 6.9 Hz, 2H, H8 of quinolinyl ring of pq), 7.28 – 7.21 (m, 2H, H5 of quinolinyl ring of pq), 7.20 – 7.08 (m, 4H, H6 and H7 of quinolinyl ring of pq), 7.05 – 6.92 (m, 2H, H4 of phenyl ring of pq), 6.82 (t, *J* = 7.5 Hz, 2H, H5 of phenyl ring of pq), 6.54 (t, *J* = 8.4 Hz, 2H, H6 of phenyl ring of pq), 5.42 (s, 1H, (NHCH$_2$CHO)OC*H*CH of TMCD), 5.25 (t, *J* = 7.5 Hz, 2H, CH$_2$ of bpy), 5.19 – 5.09 (m, 6H, O$_2$C*H*CH of TMCD), 3.99 – 3.12 (m, 102H, OC*H*(CH$_2$)(CH), OC*H*CH$_2$, NHC*H*$_2$, OCH$_2$, and OCH$_3$ of TMCD), 2.60 (s, 3H, CH$_3$ on bpy). ^{13}C NMR (150 MHz, CDCl$_3$, 298 K, TMS): δ 169.88, 156.84, 156.55, 155.38, 153.00, 151.80, 151.68, 151.02, 147.64, 147.54, 146.08,

145.90, 145.60, 145.58, 139.82, 139.71, 134.73, 134.64, 131.32, 130.83, 130.79, 128.91, 128.16, 128.04, 127.51, 127.46, 126.98, 126.79, 126.75, 124.89, 124.78, 123.72, 123.45, 122.78, 122.72, 117.15, 117.11, 99.08, 99.00, 98.93, 98.75, 98.68, 98.57, 82.11, 82.06, 81.96, 81.87, 81.80, 81.67, 81.53, 81.46, 81.05, 80.28, 80.22, 80.03, 79.60, 71.37, 71.30, 71.19, 71.14, 71.08, 70.91, 70.82, 70.42, 63.11, 61.52, 61.46, 61.16, 59.28, 59.07, 59.02, 58.97, 58.61, 58.60, 58.56, 58.50, 58.46, 58.42, 53.43, 41.58, 31.59, 22.66, 21.66, 14.12. IR (KBr) $\tilde{\nu}$/cm^{-1}: 3512 (N–H), 2926 (C–H), 1648 (C=O), 1037 (C–O–C). MALDI-TOF-MS (CCA) *m/z* found: 2244.8 [M – Cl^{-}]$^{+}$ calcd: 2241.5. HR-MS (ESI, positive mode) *m/z* found: 2240.9001 [M – Cl^{-}]$^{+}$ calcd for $IrC_{105}H_{141}N_5O_{36}$ 2240.8985.

[$Ir(bsn)_2(bpy\text{-}TMCD)$](Cl) (**3a**)

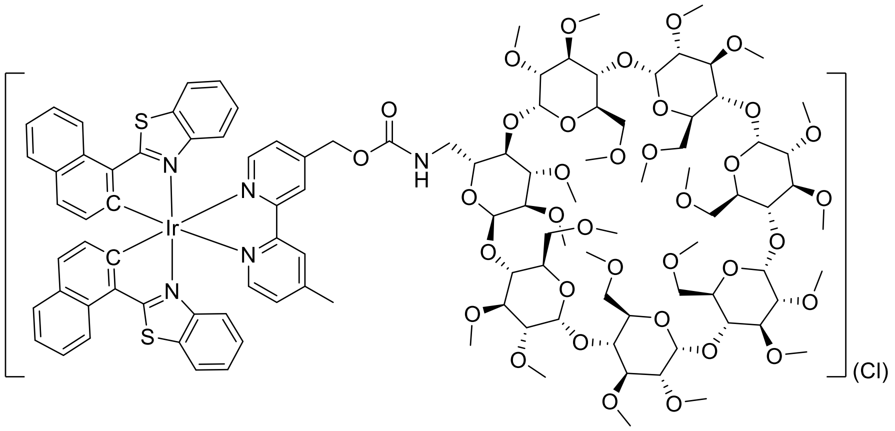

The synthetic procedure was similar to that of complex **1a**, except that [$Ir_2(bsn)_4Cl_2$] (60 mg, 0.04 mmol) was used instead of [$Ir_2(ppy)_4Cl_2$]. The solvent was removed under reduced pressure to afford complex **3a** as a red solid. Yield: 109 mg (76%). ^{1}H NMR (300 MHz, $CDCl_3$, 298 K, TMS): δ 10.09 (s, 1H, H3 of bpy), 9.77 (s, 1H, H3' of bpy), 8.68 (d, *J* = 6.3 Hz, 2H, H8 of naphthyl ring of bsn), 7.98 (d, *J* = 6.0 Hz, 2H, H5 of naphthyl ring of bsn), 7.86 (d, *J* = 4.5 Hz, 1H, H6 of bpy), 7.82 – 7.66 (m, 6H, H6' and H5 of bpy and H4 of benzothiazole ring and H7 of naphthyl ring of bsn), 7.53 – 7.40 (m, 4H, H5 and H6 of benzothiazole ring of bsn), 7.25 (s, 1H, H5' of bpy), 7.21 – 7.09 (m, 4H, H4 and H6 of naphthyl ring of bsn), 6.65 – 6.54 (m, 2H, H7 of benzothiazole ring of bsn), 6.38 – 6.28 (m, 2H, H3 of naphthyl ring of bsn), 5.49 – 5.29 (m, 3H, ($NHCH_2CHO$)OC*H*CH of TMCD and CH_2 of bpy), 5.21 – 5.10 (m, 6H, O_2C*H*CH of TMCD), 3.98 – 3.17 (m, 102H, OC*H*(CH_2)(CH), OC*H*CH_2, NHC*H*$_2$, OCH_2, and OCH_3 of TMCD), 2.73 (s, 3H, CH_3 on bpy). ^{13}C NMR (150 MHz, $CDCl_3$, 298 K, TMS): δ 178.32, 178.26, 160.73, 157.56, 156.56, 156.09, 153.97, 152.03, 148.60, 148.47, 147.91, 134.08, 134.05, 132.56, 131.66, 131.44, 131.38, 130.92, 130.89, 130.36, 128.79, 128.66, 128.51,

128.47, 125.78, 124.31, 124.13, 122.89, 121.62, 117.46, 117.34, 99.10, 99.00, 98.92, 98.71, 98.65, 98.58, 82.11, 82.06, 82.03, 81.96, 81.87, 81.84, 81.79, 81.66, 81.52, 81.12, 80.27, 80.21, 80.07, 79.98, 79.62, 71.35, 71.31, 71.15, 71.09, 70.90, 70.81, 70.47, 63.17, 61.52, 61.47, 61.46, 61.15, 61.12, 59.29, 59.07, 59.04, 58.99, 58.95, 58.61, 58.57, 58.49, 58.46, 58.42, 58.38, 53.43, 41.66, 29.70, 21.38. IR (KBr) $\tilde{\nu}$/cm^{-1}: 3444 (N–H), 2933 (C–H), 1630 (C=O), 1035 (C–O–C). MALDI-TOF-MS (CCA) *m/z* found: 2356.8 [M – Cl^{-}]$^{+}$ calcd: 2353.7. HR-MS (ESI, positive mode) *m*/*z* found: 2352.8438 [M – Cl^{-}]$^{+}$ calcd for $IrC_{109}H_{141}N_5O_{36}S_2$ 2352.8427.

[Ir(ppy)$_2$(bpy-C4)](Cl) (**1b**)

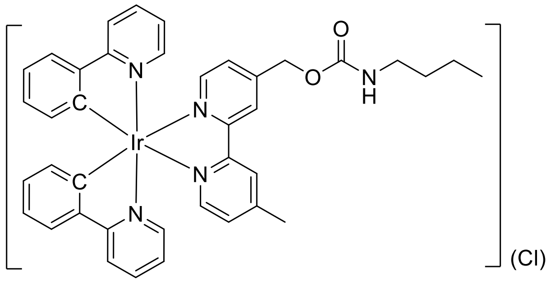

A mixture of [$Ir_2(ppy)_4Cl_2$] (64 mg, 0.06 mmol) and bpy-C4 (36 mg, 0.12 mmol) in CH_2Cl_2/MeOH (20 mL) (1:1, *v/v*) was stirred under an inert atmosphere of N_2 in the dark at 298 K for 18 h. The solvent was removed under reduced pressure and the residual yellow solid was purified by silica gel chromatography using CH_2Cl_2/MeOH (20:1, *v/v*) as the eluent. The solvent was removed under reduced pressure to afford complex **1b** as a yellow solid. Yield: 80 mg (80%). ^{1}H NMR (300 MHz, $CDCl_3$, 298 K, TMS): δ 9.95 (s, 1H, H3 of bpy), 9.51 (s, 1H, H3’ of bpy), 8.30 (t, J = 5.7 Hz, 1H, H5 of bpy), 7.97 – 7.83 (m, 2H, H6 and H6’ of bpy), 7.81 – 7.71 (m, 3H, H5’ of bpy and H3 of pyridyl ring of ppy), 7.70 – 7.61 (m, 3H, H3 of phenyl ring of ppy and NH), 7.46 (d, J = 5.7 Hz, 2H, H4 of pyridyl ring of ppy), 7.17 (t, J = 5.4 Hz, 2H, H6 of pyridyl ring of ppy), 7.05 – 6.94 (m, 4H, H5 of pyridyl ring and H4 of phenyl ring of ppy), 6.93 – 6.82 (m, 2H, H5 of phenyl ring of ppy), 6.29 (d, J = 7.5 Hz, 2H, H6 of phenyl ring of ppy), 5.33 – 5.26 (m, 2H, CH_2 of bpy), 3.19 (q, J = 7.2 Hz, 2H, $NHC\mathit{H}_2CH_2CH_2CH_3$), 2.74 (s, 3H, CH_3 on bpy), 1.59 (sext, J = 6.9 Hz, 2H, $NHCH_2C\mathit{H}_2CH_2CH_3$), 1.37 (sext, J = 7.5 Hz, 2H, $NHCH_2CH_2C\mathit{H}_2CH_3$), 0.88 (t, J = 7.5 Hz, 3H, $NHCH_2CH_2CH_2C\mathit{H}_3$). ^{13}C NMR (150 MHz, $CDCl_3$, 298 K, TMS): δ 157.04, 156.44, 155.38, 153.36, 151.50, 150.84, 150.78, 149.00,

148.93, 148.35, 143.49, 143.44, 137.99, 137.94, 131.76, 131.71, 128.86, 128.48, 124.78, 124.74, 124.41, 123.37, 123.11, 122.50, 122.44, 119.59, 119.55, 62.76, 53.47, 40.84, 31.82, 21.33, 20.15, 13.84. IR (KBr) $\tilde{\nu}$/cm^{-1}: 3467 (N–H), 1638 (C=O). ESI-MS (positive-ion mode) *m*/*z* found: 800.7 $[M - Cl^{-}]^{+}$ calcd: 800.0. HR-MS (ESI, positive mode) *m*/*z* found: 800.2515 $[M - Cl^{-}]^{+}$ calcd for $IrC_{39}H_{37}N_5O_2$ 800.2576.

[Ir(pq)$_2$(bpy-C4)](Cl) (**2b**)

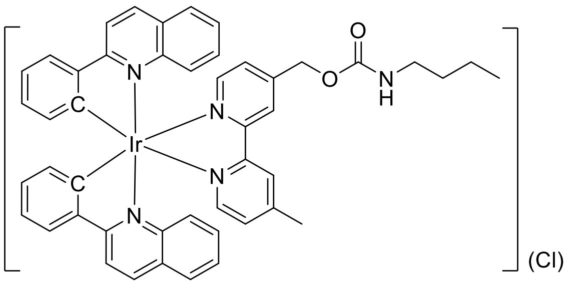

The synthetic procedure was similar to that of complex **1b**, except that [Ir$_2$(pq)$_4$Cl$_2$] (76 mg, 0.06 mmol) was used instead of [Ir$_2$(ppy)$_4$Cl$_2$]. The solvent was removed under reduced pressure to afford complex **2b** as an orange solid. Yield: 91 mg (81%). ^{1}H NMR (300 MHz, CDCl$_3$, 298 K, TMS): δ 9.66 (s, 1H, H3 of bpy), 9.17 (s, 1H, H3' of bpy), 8.50 – 8.39 (m, 1H, H5 of bpy), 8.27 – 8.11 (m, 4H, H6 and H6' of bpy and H4 of quinolinyl ring of pq), 8.04 – 7.93 (m, 3H, H5' of bpy and H3 of quinolinyl ring of pq), 7.91 – 7.82 (m, 1H, NH), 7.79 – 7.67 (m, 2H, H3 of phenyl ring of pq), 7.44 – 7.34 (m, 2H, H8 of quinolinyl ring of pq), 7.29 – 7.22 (m, 2H, H5 of quinolinyl ring of pq), 7.20 – 7.05 (m, 4H, H6 and H7 of quinolinyl ring of pq), 6.95 (t, *J* = 8.1 Hz, 2H, H4 of phenyl ring of pq), 6.79 (t, *J* = 7.5 Hz, 2H, H5 of phenyl ring of pq), 6.55 (t, *J* = 6.9 Hz, 2H, H6 of phenyl ring of pq), 5.20 (d, *J* = 6.0 Hz, 2H, CH$_2$ of bpy), 3.15 (q, *J* = 7.2 Hz, 2H, NHCH_2CH$_2$CH$_2$CH$_3$), 2.64 (s, 3H, CH$_3$ on bpy), 1.58 (sext, *J* = 4.8 Hz, 2H, NHCH$_2$CH_2CH$_2$CH$_3$), 1.37 (sext, *J* = 6.9 Hz, 2H, NHCH$_2$CH$_2$CH_2CH$_3$), 0.86 (t, *J* = 7.2 Hz, 3H, NHCH$_2$CH$_2$CH$_2$CH_3). ^{13}C NMR (150 MHz, CDCl$_3$, 298 K, TMS): δ 169.88, 169.86, 157.07, 156.36, 155.32, 153.17, 151.81, 151.70, 151.15, 147.62, 147.54, 145.89, 145.60, 139.83, 139.72, 134.73, 134.64, 131.32, 131.30, 130.81, 130.77, 128.93, 128.90,

128.22, 128.08, 127.51, 127.47, 126.96, 126.80, 126.75, 124.87, 124.78, 123.56, 122.76, 122.71, 117.15, 117.12, 62.47, 52.44, 40.78, 31.77, 21.12, 20.15, 13.82. IR (KBr) $\tilde{\nu}$/cm^{-1}: 3417 (N–H), 1638 (C=O). ESI-MS (positive-ion mode) *m*/*z* found: 900.6 $[M - Cl^{-}]^{+}$ calcd: 900.3. HR-MS (ESI, positive mode) *m*/*z* found: 900.2837 $[M - Cl^{-}]^{+}$ calcd for $IrC_{47}H_{41}N_5O_2$ 900.2889.

[Ir(bsn)$_2$(bpy-C4)](Cl) (**3b**)

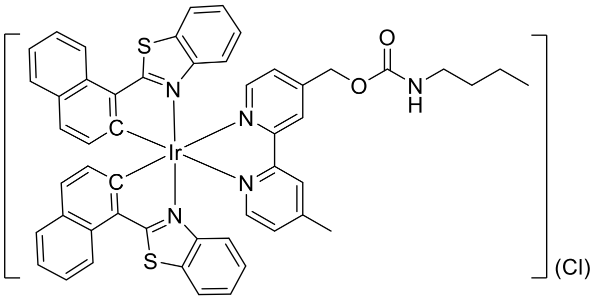

The synthetic procedure was similar to that of complex **1b**, except that [Ir$_2$(bsn)$_4$Cl$_2$] (90 mg, 0.06 mmol) was used instead of [Ir$_2$(ppy)$_4$Cl$_2$]. The solvent was removed under reduced pressure to afford complex **3b** as a red solid. Yield: 112 mg (89%). ^{1}H NMR (300 MHz, CDCl$_3$, 298 K, TMS): δ 10.18 (s, 1H, H3 of bpy), 9.63 (s, 1H, H3' of bpy), 8.68 (d, *J* = 8.4 Hz, 2H, H8 of naphthyl ring of bsn), 8.55 (t, *J* = 5.7 Hz, 1H, H6 of bpy), 7.98 (d, *J* = 8.1 Hz, 2H, H5 of naphthyl ring of bsn), 7.87 (d, *J* = 5.7 Hz, 1H, H6' of bpy), 7.82 – 7.67 (m, 5H, H4 of benzothiazole ring and H7 of naphthyl ring of bsn and H5 of bpy), 7.55 – 7.39 (m, 4H, H5 and H6 of benzothiazole ring of bsn), 7.27 (s, 1H, H5' of bpy), 7.20 – 7.09 (m, 4H, H4 and H6 of naphthyl ring of bsn), 6.60 (d, *J* = 8.4 Hz, 2H, H7 of benzothiazole ring of bsn), 6.32 (t, *J* = 8.1 Hz, 2H, H3 of naphthyl ring of bsn), 5.29 (d, *J* = 6.9 Hz, 2H, CH$_2$ of bpy), 3.23 (q, *J* = 6.9 Hz, 2H, NHCH_2CH$_2$CH$_2$CH$_3$), 2.79 (s, 3H, CH$_3$ on bpy), 1.62 – 1.58 (m, 2H, NHCH$_2$CH_2CH$_2$CH$_3$), 1.40 (sext, *J* = 7.5 Hz, 2H, NHCH$_2$CH$_2$CH_2CH$_3$), 0.91 (t, *J* = 7.2 Hz, 3H, NHCH$_2$CH$_2$CH$_2$CH_3). ^{13}C NMR (150 MHz, CDCl$_3$, 298 K, TMS): δ 178.31, 178.25, 160.81, 160.74, 157.79, 156.35, 156.01, 154.17, 152.11, 148.47, 148.44, 147.91, 134.10, 134.05, 132.55, 132.51, 131.69, 131.66, 131.43, 131.38, 130.93, 130.91, 130.89, 130.36, 128.86, 128.81, 128.66, 128.63,

128.50, 128.47, 125.80, 125.77, 124.31, 124.29, 124.21, 123.44, 122.89, 121.62, 117.44, 117.34, 62.62, 40.83, 31.79, 29.70, 28.93, 21.33, 20.16, 13.83. IR (KBr) $\tilde{\nu}$/cm^{-1}: 3519 (N–H), 1628 (C=O). ESI-MS (positive-ion mode) *m*/*z* found: 1012.5 $[M - Cl^-]^+$ calcd: 1012.2. HR-MS (ESI, positive mode) *m*/*z* found: 1012.2297 $[M - Cl^-]^+$ calcd for $IrC_{51}H_{41}N_5O_2S_2$ 1012.2331.

3-(((1*r*,3*r*,5*R*,7*S*)-Adamantan-2-ylidene)(methoxy)methyl)-2-chlorophenyl acetate (**ADE**$_{\mathbf{E}}$)

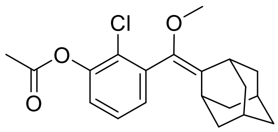

A mixture of **ADE**$_{\mathbf{OH}}$ (200 mg, 0.66 mmol) and pyridine (266 μL) in acetic anhydride (5 mL) was stirred at 80°C under an inert atmosphere of N_2 for 12 h. The solvent was evaporated to dryness yielding a pale-yellow solid, which was purified by column chromatography on silica gel using *n*-hexane/ethyl acetate (10:1, *v*/*v*) as the eluent. The solvent was removed under vacuum to afford the product as a white solid. Yield: 148 mg (65%). ^{1}H NMR (300 MHz, CD_3OD, 298 K): δ 7.30 – 7.25 (m, 1H, H4 of phenyl ring), 7.22 – 7.10 (m, 2H, H5 and H6 of phenyl ring), 3.38 – 3.26 (m, 4H, OCH_3 and H of adamantane), 2.38 (s, 3H, $OCOCH_3$), 2.10 (s, 1H, H of adamantane), 1.99 – 1.68 (m, 12H, H of adamantane). ESI-MS (positive-ion mode) *m*/*z* found: 347.4 $[M + H^+]^+$ calcd: 347.1.

2-Chloro-3-((1*r*,3*r*,5*r*,7*r*)-4'-methoxyspiro[adamantane-2,3'-[1,2]dioxetan]-4'-yl)phenyl acetate (**ADO$_E$**)

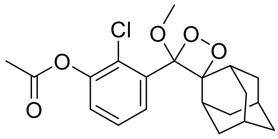

A mixture of **ADE$_E$** (100 mg, 0.29 mmol) and methylene blue (9 mg, 0.029 mmol) in dry CH_2Cl_2 (10 mL) was bubbled with O_2 at 298 K for 6 h while being irradiated with a warm light lamp (40 W). The solvent was evaporated to dryness yielding a blue solid, which was purified by column chromatography on silica gel using CH_2Cl_2/MeOH (50:1, *v*/*v*) as the eluent. The solvent was removed under vacuum to afford the product as a white solid. Yield: 73 mg (67%). ^{1}H NMR (300 MHz, CD_3OD, 298 K): δ 8.02 (d, *J* = 8.1 Hz, 1H, H6 of phenyl ring), 7.45 (t, *J* = 8.1 Hz, 1H, H5 of phenyl ring), 7.28 – 7.20 (m, 1H, H4 of phenyl ring), 3.23 (s, 3H, OCH_3), 3.03 (s, 1H, H of adamantane), 2.38 (s, 3H, $OCOCH_3$), 2.30 (d, *J* = 12.9 Hz, 1H, H of adamantane), 2.03 – 1.35 (m, 12H, H of adamantane). ESI-MS (positive-ion mode) *m*/*z* found: 379.4 $[M + H^+]^+$ calcd: 379.1.

Physical Measurements and Instrumentation

^{1}H, ^{13}C, and ^{31}P nuclear magnetic resonance (NMR) spectra were recorded on a Bruker AVANCE III 300, 400, or 600 MHz NMR spectrometer at 298 K using deuterated solvents. Positive-ion electrospray ionization (ESI) mass spectra were recorded on a Perkin Elmer Sciex API 3200MD mass spectrometer at 298 K. Matrix-assisted laser desorption/ionization-time-of-flight (MALDI-TOF) mass spectra of the samples were recorded on an Applied Biosystems 4800 Plus MALDI TOF/TOF™ Analyzer. High resolution (HR)-ESI mass spectra were recorded on a SCIEX X500R Q-TOF at 298 K. Infrared (IR) spectra of the samples in KBr pellets were recorded in the range of 4000 – 400 cm^{-1} using a Perkin Elmer FTIR–1600 spectrometer. Electronic absorption spectra were recorded on an Agilent 8453 diode array spectrophotometer. Steady-state emission spectra were obtained on a HORIBA FluoroMax-4 spectrofluorometer. Unless specified otherwise, all solutions for photophysical studies were degassed with at least four successive freeze-pump-thaw cycles and stored in a 10-cm^3 round bottomed flask equipped with a sidearm 1-cm fluorescence cuvette and sealed from the atmosphere by a Rotaflo HP6/6 quick-release Teflon stopper. Emission quantum yields were measured by the optically dilute method[9] using an aerated aqueous solution of $[Ru(bpy)_3]Cl_2$ (Φ_{em} = 0.040, λ_{ex} = 455 nm)[10] as the standard solution. The concentrations of the standard and sample solutions were adjusted until the absorbance at the excitation wavelength (455 nm) was 0.1. Emission lifetimes were measured on an Edinburgh Instruments LP920 laser flash

photolysis spectrometer using the third harmonic output (355 nm; 6 – 8 ns fwhm pulse width) of a Spectra-Physics Quanta-Ray Q-switched LAB-150 pulsed Nd:YAG laser (10 Hz) as the excitation source.

Determination of Singlet Oxygen ($^1O_2$) Generation Quantum Yields (Φ_Δ)

The Φ_Δ values of the complexes were measured by the optically dilute method[9] using $[Ru(bpy)_3]Cl_2$ in aerated CH_3CN (Φ_Δ = 0.57)[11] as a reference. An air-equilibrated CH_3CN solution (2 mL) containing the complex was introduced to a quartz cuvette of 1-cm path length. The concentrations of the reference and sample solutions were adjusted until absorbance at 450 nm was *ca*. 0.1. The solutions were excited at λ = 450 nm and the emission spectra of $^1O_2$ at 1,200 – 1,350 nm were recorded on an Edinburgh Instruments FLS980 spectrometer equipped with an R5509-73 NIR photomultiplier tube (PMT) and C9940-02 exclusive coolers. A 1,000-nm long-pass filter was placed between the sample solution and the NIR PMT detector to avoid high-order diffraction from the visible light. The Φ_Δ value of the complex was determined using the following equation, where the subscripts *s* and *r* refer to the sample and reference solutions, respectively:

$$\Phi_s = \Phi_r \left(\frac{I_r}{I_s}\right)\left(\frac{B_r}{B_s}\right)\left(\frac{n_s}{n_r}\right)^2\left(\frac{D_s}{D_r}\right)$$

where Φ is luminescence quantum yield, I is excitation intensity, B is $1 - 10^{-AL}$, A is absorbance at the excitation wavelength, L is path length in cm, n is refractive index of the solvent, and D is integrated intensity.

Supramolecular Host–Guest Interaction Studies

The supramolecular host–guest interaction between the iridium(III) TMCD complexes **1a** – **3a** and the spiroadamantyl phenoxy-1,2-dioxetane probe **ADO$_\mathbf{P}$** was studied using ^{31}P NMR spectroscopy. The ^{31}P NMR spectra of solutions of the complex ([Ir] = 0.5 – 4.0 mM for complex **1a** and 0.6 – 2.0 mM for complexes **2a** and **3a**) and probe **ADO$_\mathbf{P}$** (4.0 mM) in D_2O at 298 K were recorded, and the chemical shift of the ^{31}P resonance of the phosphate group of probe **ADO$_\mathbf{P}$** was monitored. A plot of $1/\Delta\delta$ against 1/[Ir] was constructed according to the Benesi–Hildebrand equation:

$$\frac{1}{\Delta\delta} = \frac{1}{(K_\mathrm{a}\Delta\delta_\mathrm{max}[\mathrm{Ir}])} + \frac{1}{\Delta\delta_\mathrm{max}}$$

where $\Delta\delta$ is the chemical shift change of the ^{31}P resonance of probe **ADO$_\mathbf{P}$** in the presence of the complex at a concentration of [Ir], $\Delta\delta_\mathrm{max}$ is the maximum chemical shift change of the ^{31}P resonance of probe **ADO$_\mathbf{P}$** upon full complexation with the complex, and K_a is the association constant. The K_a values were determined by dividing the y-intercept of the plot by the slope.

Chemiluminescence (CL) Measurements

All CL measurements were conducted in white 96-well plates using a SpectraMax i3x Multi-Mode Microplate Reader (Molecular Devices) in luminescence mode.

For CL spectra measurements, an aliquot (10 μL) of probe **ADO_P** (2 mM in H_2O) and an aliquot (10 μL) of TMCD-containing compounds (TMCD and the TMCD complexes **1a** – **3a** and **3c**; 2 mM in H_2O) were added to Tris buffer (50 mM, pH 9.0) (80 μL), and the mixture was incubated in the dark at 298 K for 1 h. The CL spectra were recorded immediately after the addition of an aliquot (100 μL) of ALP (2 unit mol^{-1}). The final concentrations of probe **ADO_P**, TMCD-containing compounds, and ALP were 100 μM, 100 μM, and 1 unit mol^{-1}, respectively. Control samples contained TMCD (100 μM) and TMCD-free complexes (100 μM) for comparison with the TMCD complexes (100 μM). The overlap integral $J(\lambda)$ for the spectral overlap between the CL spectrum of probe **ADO_P** in Tris buffer (50 mM, pH 9.0) in the presence of ALP, and the absorption spectra of the TMCD complexes **1a** – **3a** and **3c** in H_2O, was calculated based on the following equation:

$$J(\lambda) = \int_0^\infty F_{\mathrm{D}}(\lambda) \times \varepsilon_{\mathrm{A}}(\lambda) \times \lambda^4 \mathrm{d}\lambda$$

where F_{D} is the corrected CL intensity of the donor, probe **ADO_P**, upon ALP activation, with the emission intensity normalized to unity, and ε_{A} is the extinction coefficient of the acceptor, complexes **1a** – **3a** and **3c**. The chemiluminescence resonance energy transfer (CRET)

efficiency was calculated as the ratio of the acceptor emission integral to the total emission integral under chemiexcitation.[12]

For CL kinetic profile measurements, an aliquot (10 μL) of phenoxy-1,2-dioxetanes (probes **ADO$_P$**, **ADO$_E$**, and **ADO$_G$**; 200 μM in H_2O) and an aliquot (10 μL) of TMCD-containing compounds (TMCD and the TMCD complexes **1a** – **3a** and **3c**; 2 mM in H_2O) were added to Tris buffer (50 mM, pH 9.0; for probe **ADO$_P$**) or PBS (1X, pH 7.4; for probes **ADO$_E$** and **ADO$_G$**) (80 μL), and the mixture was incubated in the dark at 298 K for 1 h. The CL kinetic profiles were recorded immediately and monitored after the addition of an aliquot (100 μL) of the respective enzyme (ALP, PLE, and β-gal; 2 unit mol^{-1}). The final concentrations of phenoxy-1,2-dioxetanes, TMCD-containing compounds, and enzymes were 10 μM, 100 μM, and 1 unit mol^{-1}, respectively.

The CL quantum yields (Φ_{CL}) were determined using luminol (10 μM) in sodium phosphate buffer (0.1 M, pH 11.6) in the presence of H_2O_2 (0.01%) (Φ_{CL} = 1.14 × 10^{-2} Einstein mol^{-1})[13] as a reference. The Φ_{CL} values were calculated using the following equation, where the subscripts *s* and *r* refer to the sample and reference solutions, respectively:

$$\Phi_s = \Phi_r \left(\frac{n_r}{n_s}\right)\left(\frac{Q_s}{Q_r}\right)\left(\frac{f(\lambda_s)}{f(\lambda_r)}\right)$$

where Φ is CL quantum yield, *n* is the number of moles of chemiluminescent agent, *Q* is integrated intensity under kinetic curve, and $f(\lambda)$ is sensitivity of the detector at the emission

wavelength. The CL half-lives ($t_{1/2}$) were calculated by fitting the decay portion of the kinetic curve to a single exponential decay function.

For the enzyme inhibition experiments, ALP, PLE, and β-gal (2 unit mol^{-1}) were pretreated with PIC3 (1:5 solution), BNPP (20 mM), and EDTA (40 mM), respectively, and incubated at 298 K for 1 h before being added to the solutions containing the corresponding phenoxy-1,2-dioxetane probes and TMCD-containing compounds. The subsequent procedures were the same as described above.

For the AD competition experiments, TMCD-containing compounds (200 μM) were simultaneously incubated with probe **ADO_E** (20 μM) and AD (2 mM) in PBS (1X, pH 7.4) at 298 K for 1 h before the addition of PLE (2 unit mol^{-1}). The subsequent procedures were the same as described above.

CRET-Induced Reactive Oxygen Species (ROS) Generation *In Vitro*

The CRET-induced ROS generation capability of adduct **ADO_P–3a** was examined using $DCFH_2$ as an indicator. A solution (1 mL) of probe **ADO_P** (1 mM) and complex **3a** (10 μM) in PBS (1X, pH 7.4) was prepared and incubated in the dark at 298 K for 1 h. Subsequently, an aliquot (2 μL) of $DCFH_2$ (20 mM in DMSO) was added, followed by the addition of ALP (1 mL, 2 unit mL^{-1}) was added. The final concentrations of probe **ADO_P**, complex **3a**, $DCFH_2$, and ALP were 500 μM, 5 μM, 20 μM, and 1 unit mol^{-1}, respectively. The fluorescence

spectrum of the solution (λ_{ex} = 480 nm, λ_{em} = 500 – 600 nm) was recorded at 0, 1, 2, 4, and 6 h after the addition of ALP, with the sample kept in the dark between measurements. Samples without ALP served as negative controls.

To identify the types of ROS generated, SOSG, DHR123, and HPF were used as indicators for $^1O_2$, superoxide anion radical ($O_2^{\bullet-}$), and hydroxyl radical ($HO^{\bullet}$), respectively. The sample preparation and reagent concentrations were the same as described above, except that $DCFH_2$ (20 μM) was replaced with the appropriate indicator (10 μM).

To confirm the origin of $^1O_2$ generation, a solution (1 mL) of probe **ADO_P** (1 mM), complex **3a** (10 μM), and SOSG (20 μM) in PBS (1X, pH 7.4) was prepared in a quartz cuvette sealed with a septum and purged with N_2 in the dark for 30 min prior to the addition of ALP (1 mL, 2 unit mL^{-1}), which was also gently purged with N_2 for 30 min. The cuvette was sealed to prevent air exposure and kept in the dark between measurements.

Cell Culture

HeLa and HEK293 cells were cultured in growth medium containing DMEM with 10% FBS and 1% penicillin/streptomycin at 37°C under a 5% CO_2 atmosphere. They were subcultured every 2 – 3 days.

Cellular Uptake

HeLa cells in growth medium were seeded in a 35-mm tissue culture dish and incubated at 37°C under a 5% CO_2 atmosphere for 48 h. The growth medium was then removed and the cells were incubated with complexes **1a** – **3a** (5 μM) in growth medium, or complexes **1b** – **3b** (5 μM) in growth medium/DMSO (99:1, *v*/*v*), at 37°C under a 5% CO_2 atmosphere for 2 h. After the treatment, the medium was removed, and the cells were washed with PBS (1 mL × 3). The cells were then trypsinized and harvested with PBS (2 mL). The resultant solution was heated with 65% HNO_3 (2 mL) at 70°C for 2 h, cooled to room temperature, and analyzed by NexION 2000 ICP-MS (PerkinElmer SCIEX Instruments).

MTT Assays

HeLa cells in growth medium were seeded in 96-well flat-bottomed microplates (*ca.* 10,000 cells per well) and incubated at 37°C under a 5% CO_2 atmosphere for 48 h. The growth medium was then replaced with complexes **1a** – **3a** in growth medium or complexes **1b** – **3b** in growth medium/DMSO (99:1, *v*/*v*), with concentrations ranging from 10^{-3} to 10^{-8} M. After incubation at 37°C under a 5% CO_2 atmosphere for 2 h, the medium was removed, and the cells were replenished with phenol red-free growth medium. One of the microplates was kept in the dark for 10 min, while the other microplate was irradiated with an LED (450 nm, 15.5 mW cm^{-2}) cellular photocytotoxicity irradiator (PURI Materials, Shenzhen, China) for 10 min. After the

treatment, the medium was replaced with fresh growth medium and the cells were incubated at 37°C under a 5% CO_2 atmosphere for an additional 20 h. After this incubation, the medium was replaced with fresh growth medium (90 μL) and a solution of MTT in PBS (10 μL, 5 mg mL^{-1}), and the cells were incubated at 37°C under a 5% CO_2 atmosphere for 4 h. The growth medium was then removed, and DMSO (100 μL) was added to each well. The microplates were incubated at 37°C under a 5% CO_2 atmosphere for 15 min. The absorbance of the solutions at 570 nm was measured with a SPECTRAmax 340 microplate reader (Molecular Devices Corp., Sunnyvale, CA).

Live-Cell Confocal Imaging

HeLa cells in growth medium were seeded on a sterilized coverslip in a 35-mm tissue culture dish and incubated at 37°C under a 5% CO_2 atmosphere for 48 h. The growth medium was then removed and the cells were incubated with complexes **1a** (20 μM; λ_{ex} = 405 nm, λ_{em} = 550 – 650 nm), **2a**, **3a** (5 μM; λ_{ex} = 405 nm, λ_{em} = 550 – 650 nm) in growth medium, or complexes **1b** – **3b** (5 μM; λ_{ex} = 405 nm, λ_{em} = 550 – 650 nm) in growth medium/DMSO (99:1, *v*/*v*), at 37°C under a 5% CO_2 atmosphere for 2 h. After the treatment, the medium was removed, and the cells were washed with PBS (1 mL × 3) before being imaged using a Leica TCS SPE (inverted configuration) confocal microscope with a 63× oil-immersion objective lens. For the co-staining experiments, after treatment with the complexes and washing with PBS, the cells

were treated with MitoTracker Deep Red (100 nM; λ_{ex} = 635 nm, λ_{em} = 650 – 680 nm) in growth medium at 37°C under a 5% CO_2 atmosphere for 20 min. After the treatment, the medium was removed, and the cells were washed with PBS (1 mL × 3) before being imaged.

CRET-Induced ROS Generation in Cells

HeLa and HEK293 cells in growth medium were seeded on a sterilized coverslip in a 35-mm tissue culture dish and grown at 37°C under a 5% CO_2 atmosphere for 48 h. The growth medium was then replaced with fresh growth medium containing probe **ADO$_P$** (500 μM), complex **3a** (5 μM), or adduct **ADO$_P$–3a** ([**ADO$_P$**] = 500 μM, [**3a**] = 5 μM) and incubated in the dark at 37°C under a 5% CO_2 atmosphere for 4 h. After the treatment, the medium was removed, and the cells were washed with PBS (1 mL × 3) before being incubated in fresh growth medium in the dark at 37°C under a 5% CO_2 atmosphere for an additional 20 h. After this incubation, the medium was removed, and the cells were washed with PBS (1 mL × 3) and treated with DCFH-DA (5 μM; λ_{ex} = 488 nm, λ_{em} = 510 – 530 nm) in fresh growth medium for 30 min. The medium was then removed, and the cells were washed with PBS (1 mL × 3) before imaging using a Leica TCS SP8 confocal microscope with a 63× oil-immersion objective lens. For the ALP inhibition experiments, HeLa cells were pretreated with DQB (20 μM) in growth medium/DMSO (99:1, *v*/*v*) or PLC (0.2 unit mL^{-1}) in growth medium at 37°C under a 5% CO_2 atmosphere for 1 h and washed with PBS (1 mL × 3) prior to the treatment of

probe **ADO$_P$**, complex **3a**, or adduct **ADO$_P$–3a**. The subsequent procedures were the same as described above.

"Dark" Photodynamic Therapy (PDT) in Cells

HeLa and HEK293 cells in growth medium were seeded in 96-well flat-bottomed microplates (*ca.* 10,000 cells per well) and incubated at 37°C under a 5% CO_2 atmosphere for 48 h. The growth medium was then replaced with fresh growth medium containing probe **ADO$_P$** (0 – 800 μM), complex **3a** (5 μM), or adduct **ADO$_P$–3a** ([**ADO$_P$**] = 0 – 800 μM, [**3a**] = 5 μM) and incubated in the dark at 37°C under a 5% CO_2 atmosphere for 4 h. After the treatment, the medium was removed, and the cells were washed with PBS (100 μL × 3) before being incubated in fresh growth medium in the dark at 37°C under a 5% CO_2 atmosphere for an additional 20 h. After this incubation, the medium was replaced with fresh growth medium (90 μL) and a solution of MTT in PBS (10 μL, 5 mg mL^{-1}), and the cells were incubated at 37°C under a 5% CO_2 atmosphere for 4 h. The growth medium was then removed, and DMSO (100 μL) was added to each well. The microplates were incubated at 37°C under a 5% CO_2 atmosphere for 15 min. The absorbance of the solutions at 570 nm was measured with a SPECTRAmax 340 microplate reader (Molecular Devices Corp., Sunnyvale, CA).

Live/Dead Cell Staining Assays

HeLa cells in growth medium were seeded in 35-mm tissue culture dishes and incubated at 37°C under a 5% CO_2 atmosphere for 48 h. The growth medium was then replaced with fresh growth medium containing probe **ADO$_P$** (600 μM), complex **3a** (5 μM), or adduct **ADO$_P$–3a** ([**ADO$_P$**] = 300 or 600 μM, [**3a**] = 5 μM) and incubated in the dark at 37°C under a 5% CO_2 atmosphere for 4 h. After the treatment, the medium was removed, and the cells were washed with PBS (1 mL × 3) before being incubated in fresh growth medium in the dark at 37°C under a 5% CO_2 atmosphere for an additional 20 h. After this incubation, the medium was removed, and the cells were washed with PBS (1 mL × 3) and treated with Calcein-AM (1 μM; λ_{ex} = 488 nm, λ_{em} = 510 – 540 nm) and PI (10 μM; λ_{ex} = 532 nm, λ_{em} = 610 – 640 nm) in fresh growth medium for 30 min. The medium was then removed, and the cells were washed with PBS (1 mL × 3) before imaging using a Leica TCS SPE (inverted configuration) confocal microscope with a 63× oil-immersion objective lens. For comparison studies, HeLa cells were incubated with complex **3a** (5 μM) in growth medium in the dark at 37°C under a 5% CO_2 atmosphere for 4 h. After removing the medium and washing with PBS (1 mL × 3), the cells were replenished with phenol red-free growth medium and irradiated with an LED (450 nm, 15.5 mW cm^{-2}) cellular photocytotoxicity irradiator (PURI Materials, Shenzhen, China) for 10 min. After irradiation, the medium was removed, and the cells were incubated in fresh growth

medium in the dark at 37°C under a 5% CO_2 atmosphere for 20 h. The subsequent procedures were the same as described above.

“Dark” PDT in 3D Multicellular Tumor Spheroids (MCTSs)

HeLa cells in growth medium (2×10^4 cells per mL) were seeded in two 96-well cell carrier spheroid ultra-low-attachment microplates and incubated at 37°C under a 5% CO_2 atmosphere for one week. The growth medium was replaced with fresh growth medium every 3 days, resulting in the formation of HeLa MCTSs with a diameter over 250 μm. The growth medium was then replaced with fresh growth medium containing probe **ADO$_\mathbf{P}$** (800 μM), complex **3a** (5 μM), or adduct **ADO$_\mathbf{P}$–3a** ([**ADO$_\mathbf{P}$**] = 800 μM, [**3a**] = 5 μM) and incubated in the dark at 37°C under a 5% CO_2 atmosphere for 4 h. After the treatment, the medium was removed, and the cells were washed with PBS (100 μL × 3) before being incubated in fresh growth medium in the dark at 37°C under a 5% CO_2 atmosphere for an additional 20 h. After this incubation, the medium was removed, and the MCTSs were washed with PBS (100 μL × 3) and treated with Calcein-AM (1 μM; λ_{ex} = 488 nm, λ_{em} = 510 – 540 nm) and PI (10 μM; λ_{ex} = 532 nm, λ_{em} = 610 – 640 nm) in fresh growth medium for 30 min. The medium was then removed, and the MCTSs were washed with PBS (100 μL × 3) before imaging using a Leica TCS SPE (inverted configuration) confocal microscope with a 10× objective lens.

Annexin V Staining Assays

HeLa cells in growth medium were seeded in 35-mm tissue culture dishes and incubated at 37°C under a 5% CO_2 atmosphere for 48 h. The growth medium was then replaced with fresh growth medium containing probe **ADO$_\mathbf{P}$** (600 μM), complex **3a** (5 μM), or adduct **ADO$_\mathbf{P}$–3a** ([**ADO$_\mathbf{P}$**] = 600 μM, [**3a**] = 5 μM) and incubated in the dark at 37°C under a 5% CO_2 atmosphere for 4 h. After the treatment, the medium was removed, and the cells were washed with PBS (1 mL × 3) before being incubated in fresh growth medium in the dark at 37°C under a 5% CO_2 atmosphere for an additional 20 h. After this incubation, the medium was removed, and the cells were washed with PBS (1 mL × 3) and treated with Alexa Fluor 647–Annexin V conjugate (5 μL; λ_{ex} = 633 nm, λ_{em} = 650 – 680 nm) in fresh growth medium for 15 min. The medium was then removed, and the cells were washed with PBS (1 mL × 3) before imaging using a Leica TCS SPE (inverted configuration) confocal microscope with a 63× oil-immersion objective lens. For comparison studies, HeLa cells were incubated with complex **3a** (5 μM) in growth medium in the dark at 37°C under a 5% CO_2 atmosphere for 4 h. After removing the medium and washing with PBS (1 mL × 3), the cells were replenished with phenol red-free growth medium and irradiated with an LED (450 nm, 15.5 mW cm^{-2}) cellular photocytotoxicity irradiator (PURI Materials, Shenzhen, China) for 10 min. After irradiation, the medium was removed, and the cells were incubated in fresh growth medium in the dark at 37°C under a 5% CO_2 atmosphere for 20 h. The subsequent procedures were the same as described above.

Table S1. Electronic absorption spectral data of the iridium(III) complexes at 298 K.

Complex	Solvent	λ_{abs}/nm (ε/dm^3 mol^{-1} cm^{-1})
**1a**	CH_2Cl_2	260 (47,400), 276 sh (39,680), 312 sh (20,280), 340 sh (9,110), 383 sh (5,650), 418 sh (3,235)
	CH_3CN	260 (50,340), 276 sh (39,855), 312 sh (19,350), 340 sh (9,000), 383 sh (5,350), 418 sh (2,850)
	H_2O	256 (45,900), 308 sh (20,260), 336 sh (8,825), 377 sh (5,835), 410 sh (3,175)
**2a**	CH_2Cl_2	272 (53,115), 285 sh (49,150), 308 sh (24,610), 336 (24,230), 351 sh (21,795), 442 (5,420)
	CH_3CN	272 (54,120), 285 sh (46,280), 308 sh (24,155), 336 (23,825), 351 sh (20,455), 442 (5,130)
	H_2O	268 (50,450), 309 sh (23,080), 337 (21,915), 349 sh (20,430), 436 (4,780)
**3a**	CH_2Cl_2	274 (49,810), 285 sh (40,935), 308 sh (24,240), 347 (30,025), 381 sh (17,575), 404 (13,050), 441 (8,915), 475 (9,065)
	CH_3CN	274 (48,510), 285 sh (38,440), 308 sh (23,780), 347 (29,210), 381 sh (15,990), 404 (11,945), 441 (8,515), 475 (8,140)

	H_2O	272 (39,220), 293 sh (29,430), 310 sh (20,770), 349 (23,295), 385 sh (13,270), 408 (10,255), 456 (6,845), 468 (7,000)
**1b**	CH_2Cl_2	260 (48,810), 276 (39,185), 312 sh (20,560), 340 sh (9,140), 383 sh (5,520), 418 (3,275)
	CH_3CN	260 (46,340), 276 (38,485), 312 sh (18,820), 340 sh (9,080), 383 sh (5,415), 418 (3,045)
**2b**	CH_2Cl_2	272 (50,135), 285 sh (46,115), 308 sh (22,805), 336 (22,675), 351 sh (20,325), 442 (4,635)
	CH_3CN	272 (48,300), 285 sh (41,360), 308 sh (21,865), 336 (21,780), 351 sh (18,670), 442 (4,785)
**3b**	CH_2Cl_2	274 (44,500), 285 sh (35,595), 308 sh (21,585), 347 (26,550), 381 sh (15,205), 404 (11,150), 441 (7,505), 475 (7,860)
	CH_3CN	274 (43,635), 285 sh (35,325), 308 sh (22,105), 347 (27,160), 381 sh (14,775), 404 (11,050), 441 (7,860), 475 (7,620)

Table S2. CL maxima (λ_{max}), quantum yields (Φ_{CL}), and half-lives ($t_{1/2}$) of probe **ADOP** (10 μM), adduct **ADOP**–TMCD ([**ADOP**] = 10 μM, [TMCD] = 100 μM), and adducts **ADOP–1a**, **ADOP–2a**, and **ADOP–3a** ([**ADOP**] = 10 μM, [Ir] = 100 μM) in Tris buffer (50 mM, pH 9.0) at 298 K in the presence of ALP (1 unit mol^{-1}).

Probe/Adduct	λ_{max}/nm	Φ_{CL}/Einstein mol^{-1}	$t_{1/2}$/min
**ADOP**	478	1.12×10^{-2}	22.7
**ADOP**–TMCD	478	1.58×10^{-2}	60.7
**ADOP–1a**	536	1.31×10^{-2}	37.5
**ADOP–2a**	564	28.43×10^{-2}	84.0
**ADOP–3a**	604	6.01×10^{-2}	83.9

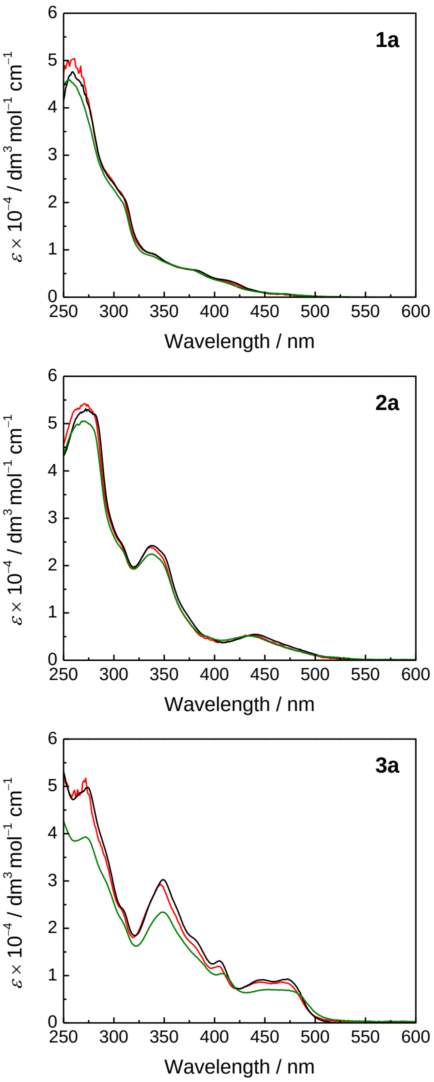

Figure S1. Electronic absorption spectra of complexes **1a** – **3a** in CH_2Cl_2 (black), CH_3CN (red), and H_2O (green) at 298 K.

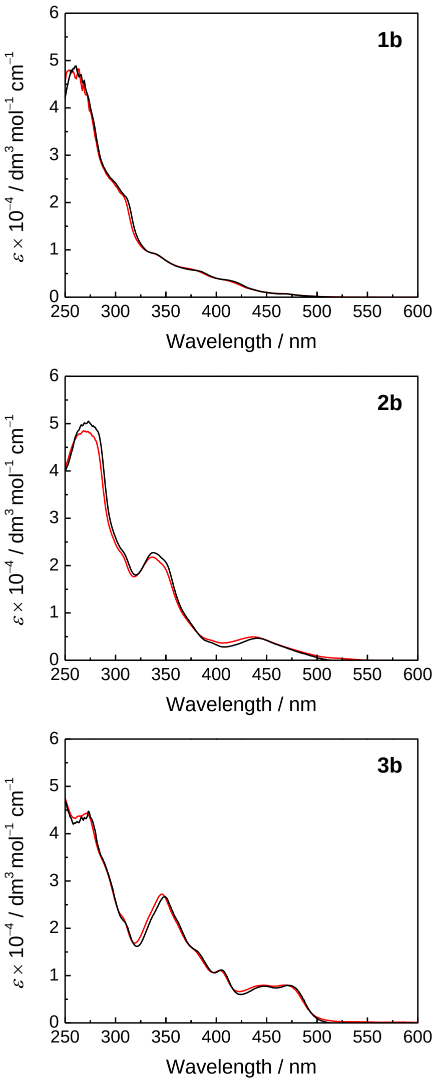

Figure S2. Electronic absorption spectra of complexes **1b** – **3b** in CH_2Cl_2 (black) and CH_3CN (red) at 298 K.

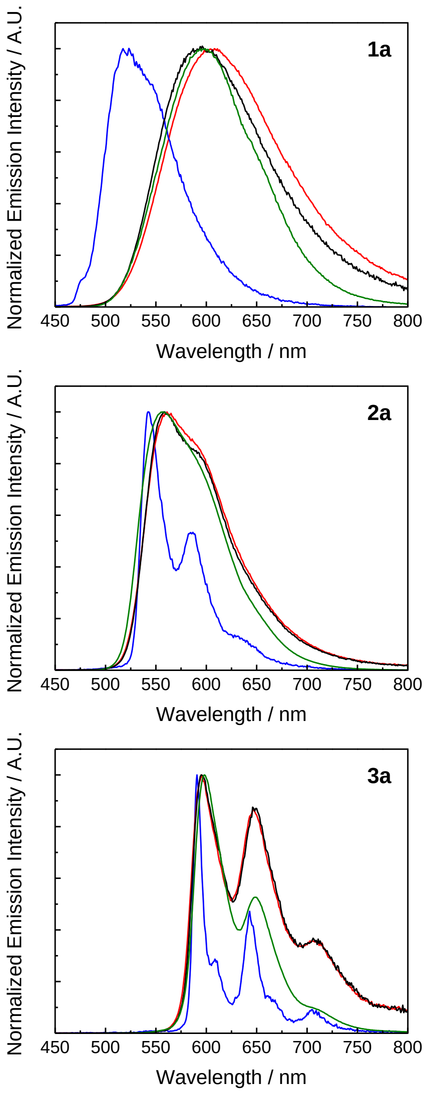

Figure S3. Normalized emission spectra of complexes **1a** – **3a** in CH_2Cl_2 (black), CH_3CN (red), and H_2O (green) at 298 K and alcohol glass (blue) at 77 K.

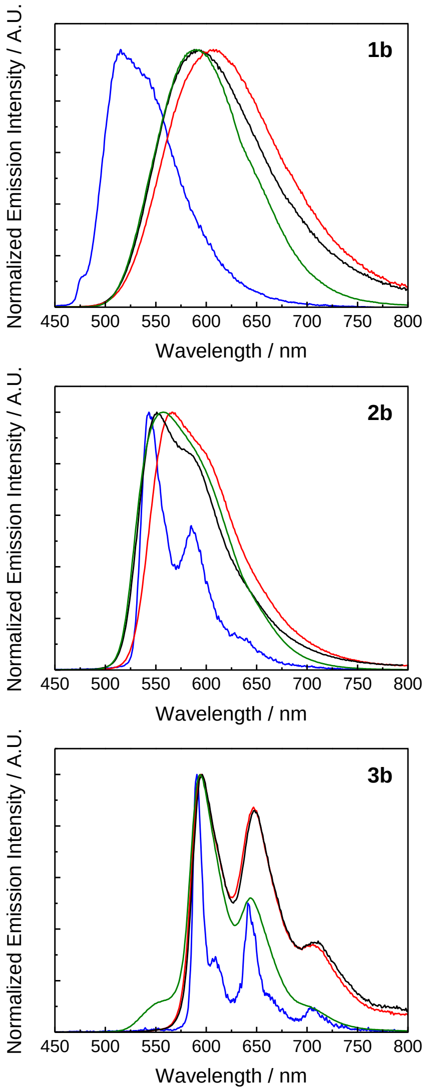

Figure S4. Normalized emission spectra of complexes **1b** – **3b** in CH_2Cl_2 (black), CH_3CN (red), and H_2O/MeOH (7:3, *v*/*v*) (green) at 298 K and alcohol glass (blue) at 77 K.

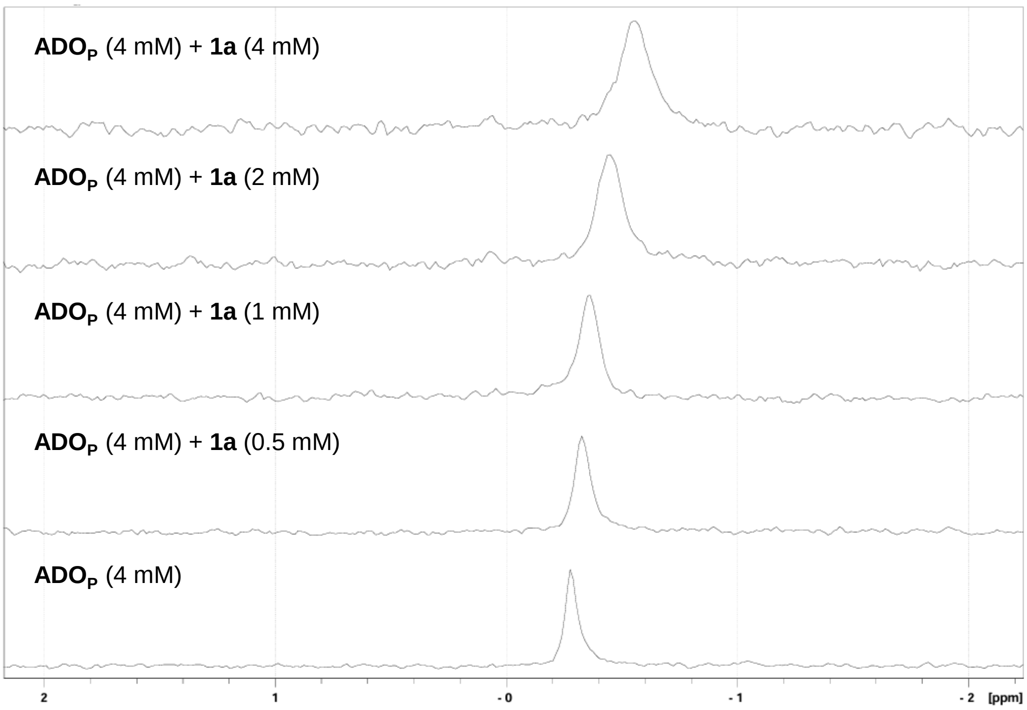

Figure S5. ^{31}P NMR spectra of probe **ADOP** (4 mM) in D_2O at 298 K in the absence and presence of complex **1a** (0.5, 1, 2, and 4 mM).

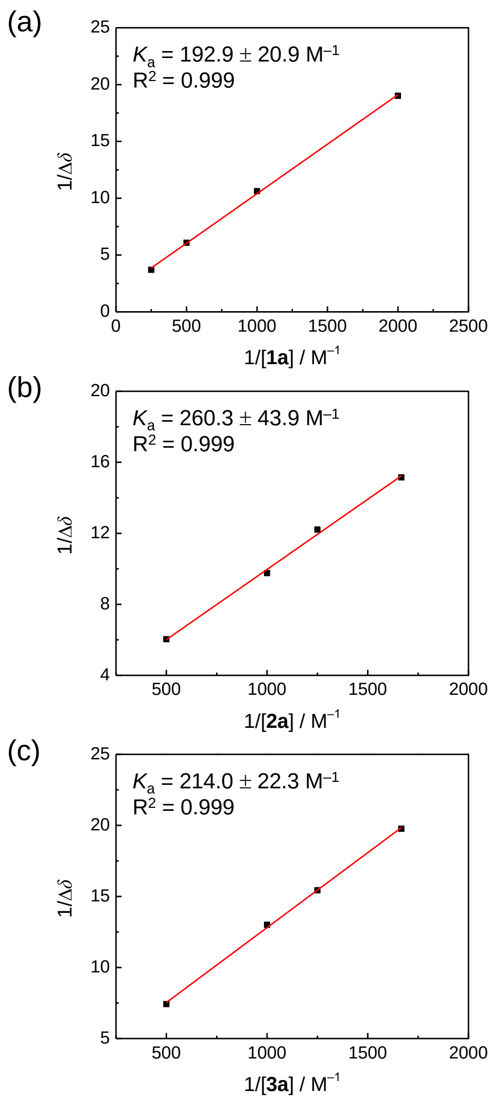

Figure S6. Benesi–Hildebrand plots for the supramolecular host–guest interaction of (a) complex **1a**, (b) complex **2a**, and (c) complex **3a** with **ADOP** in D_2O at 298 K.

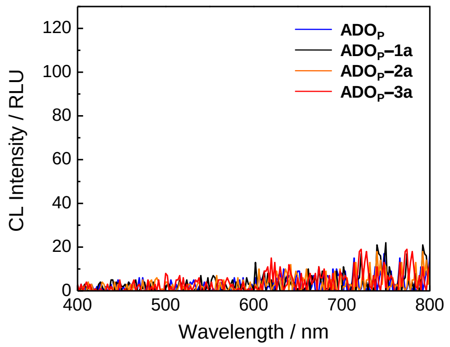

Figure S7. CL spectra of probe **ADO$_P$** (100 μM) (blue), adduct **ADO$_P$–1a** (black), adduct **ADO$_P$–2a** (orange), and adduct **ADO$_P$–3a** (red) ([**ADO$_P$**] = 100 μM, [Ir] = 100 μM) in Tris buffer (50 mM, pH 9.0) in the absence of ALP.

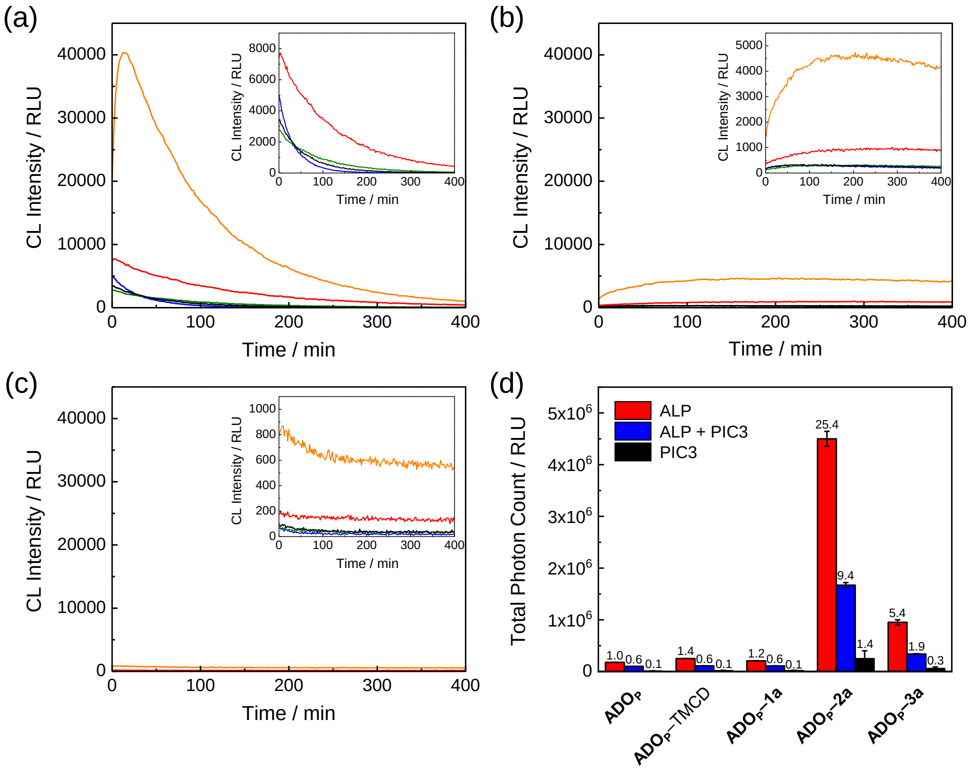

Figure S8. CL kinetic profiles of probe **ADOP** (10 μM) (blue), adduct **ADOP**–TMCD ([**ADOP**] = 10 μM, [TMCD] = 100 μM) (green), adduct **ADOP–1a** (black), adduct **ADOP–2a** (orange), and adduct **ADOP–3a** (red) ([**ADOP**] = 10 μM, [Ir] = 100 μM) in Tris buffer (50 mM, pH 9.0) at 298 K in the presence of (a) ALP (1 unit mL^{-1}), (b) PIC3 (1:10 solution)-pretreated ALP (1 unit mL^{-1}), or (c) PIC3 (1:10 solution). Insets: Enlarged view of the CL kinetic profiles for probe **ADOP** (blue), adduct **ADOP**–TMCD (green), adduct **ADOP–1a** (black), adduct **ADOP–2a** (orange), and adduct **ADOP–3a** (red) under the respective treatments. (d) Total photon counts for probe **ADOP** (10 μM), adduct **ADOG**–TMCD ([**ADOP**] = 10 μM, [TMCD] = 100 μM), adduct **ADOP–1a**, adduct **ADOP–2a**, and adduct **ADOP–3a** ([**ADOG**] = 10 μM, [Ir] = 100 μM) in Tris buffer (50 mM, pH 9.0) at 298 K in the presence of ALP (1 unit mL^{-1}) (red), PIC3 (1:10 solution)-pretreated ALP (1 unit mL^{-1}) (blue), or (c) PIC3 (1:10 solution) (black).

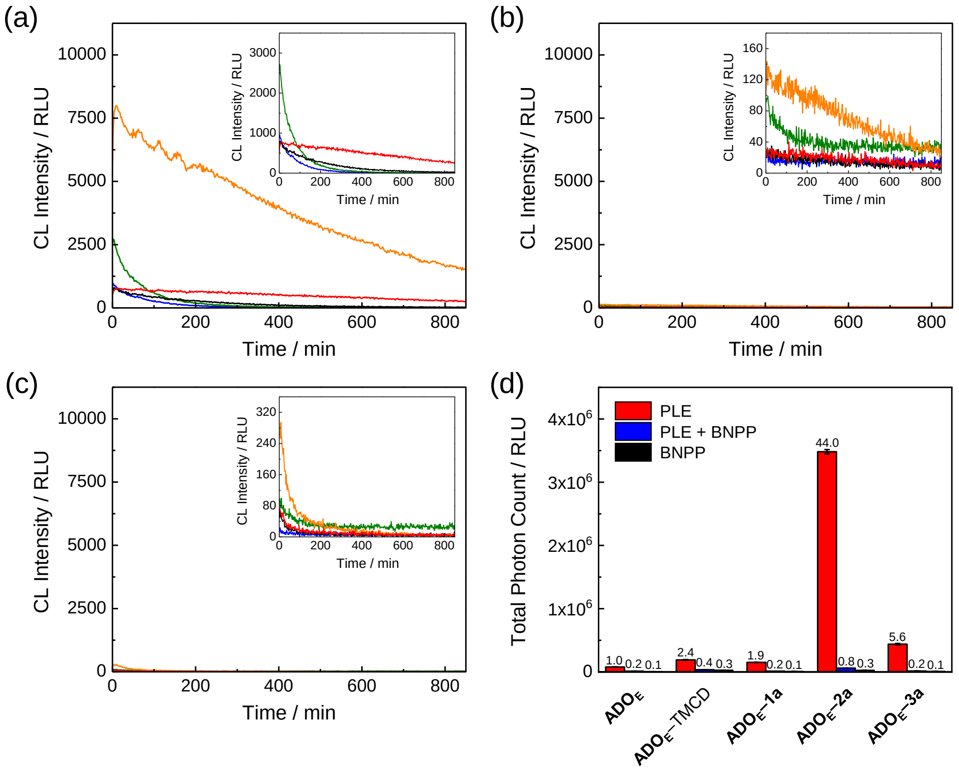

Figure S9. CL kinetic profiles of probe **ADO$_{E}$** (10 μM) (blue), adduct **ADO$_{E}$**–TMCD ([**ADO$_{E}$**] = 10 μM, [TMCD] = 100 μM) (green), adduct **ADO$_{E}$–1a** (black), adduct **ADO$_{E}$–2a** (orange), and adduct **ADO$_{E}$–3a** (red) ([**ADO$_{E}$**] = 10 μM, [Ir] = 100 μM) in PBS (1X, pH 7.4) at 298 K in the presence of (a) PLE (1 unit mL^{-1}), (b) BNPP (10 mM)-pretreated PLE (1 unit mL^{-1}), or (c) BNPP (10 mM). Insets: Enlarged view of the CL kinetic profiles for probe **ADO$_{E}$** (blue), adduct **ADO$_{E}$**–TMCD (green), adduct **ADO$_{E}$–1a** (black), adduct **ADO$_{E}$–2a** (orange), and adduct **ADO$_{E}$–3a** (red) under the respective treatments. (d) Total photon counts for probe **ADO$_{E}$** (10 μM), adduct **ADO$_{E}$**–TMCD ([**ADO$_{E}$**] = 10 μM, [TMCD] = 100 μM), adduct **ADO$_{E}$–1a**, adduct **ADO$_{E}$–2a**, and adduct **ADO$_{E}$–3a** ([**ADO$_{E}$**] = 10 μM, [Ir] = 100 μM) in PBS (1X, pH 7.4) at 298 K in the presence of PLE (1 unit mL^{-1}) (red), BNPP (10 mM)-pretreated PLE (1 unit mL^{-1}) (blue), or (c) BNPP (10 mM) (black).

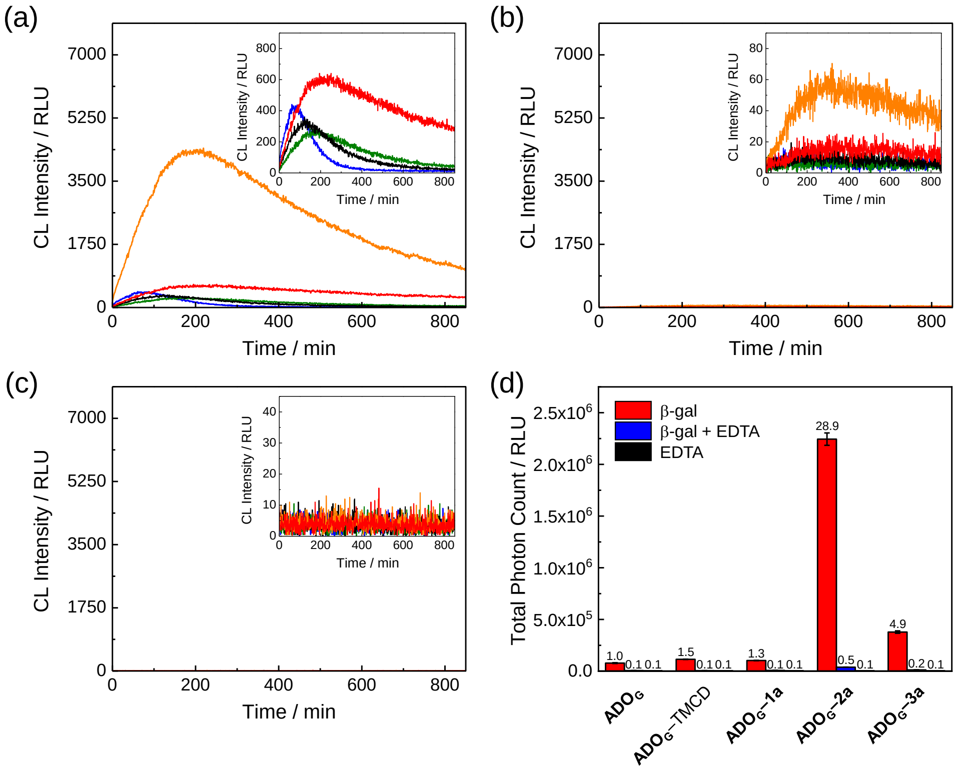

Figure S10. CL kinetic profiles of probe **ADO$_{\rm G}$** (10 μM) (blue), adduct **ADO$_{\rm G}$**–TMCD ([**ADO$_{\rm G}$**] = 10 μM, [TMCD] = 100 μM) (green), adduct **ADO$_{\rm G}$–1a** (black), adduct **ADO$_{\rm G}$–2a** (orange), and adduct **ADO$_{\rm G}$–3a** (red) ([**ADO$_{\rm G}$**] = 10 μM, [Ir] = 100 μM) in PBS (1X, pH 7.4) at 298 K in the presence of (a) β-gal (1 unit mL^{-1}), (b) EDTA (20 mM)-pretreated β-gal (1 unit mL^{-1}), or (c) EDTA (20 mM). Insets: Enlarged view of the CL kinetic profiles for probe **ADO$_{\rm G}$** (blue), adduct **ADO$_{\rm G}$**–TMCD (green), adduct **ADO$_{\rm G}$–1a** (black), adduct **ADO$_{\rm G}$–2a** (orange), and adduct **ADO$_{\rm G}$–3a** (red) under the respective treatments. (d) Total photon counts for probe **ADO$_{\rm G}$** (10 μM), adduct **ADO$_{\rm G}$**–TMCD ([**ADO$_{\rm G}$**] = 10 μM, [TMCD] = 100 μM), adduct **ADO$_{\rm G}$–1a**, adduct **ADO$_{\rm G}$–2a**, and adduct **ADO$_{\rm G}$–3a** ([**ADO$_{\rm G}$**] = 10 μM, [Ir] = 100 μM) in PBS (1X, pH 7.4) at 298 K in the presence of β-gal (1 unit mL^{-1}) (red), EDTA (20 mM)-pretreated β-gal (1 unit mL^{-1}) (blue), or (c) EDTA (20 mM) (black).

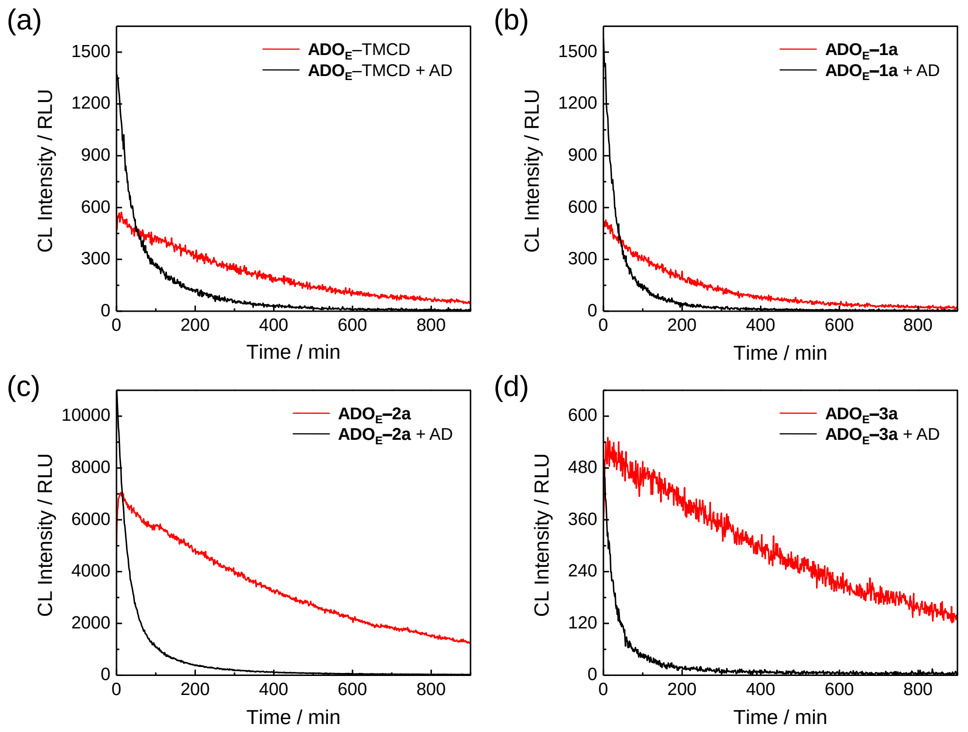

Figure S11. CL kinetic profiles of (a) adduct **ADO$_E$**–TMCD ([**ADO$_E$**] = 10 μM, [TMCD] = 100 μM), (b) adduct **ADO$_E$–1a**, (c) adduct **ADO$_E$–2a**, and (d) adduct **ADO$_E$–3a** ([**ADO$_E$**] = 10 μM, [Ir] = 100 μM) in PBS (1X, pH 7.4) in the presence of PLE (1 unit mL^{-1}) without (red) or with (black) the addition of AD (1 mM).

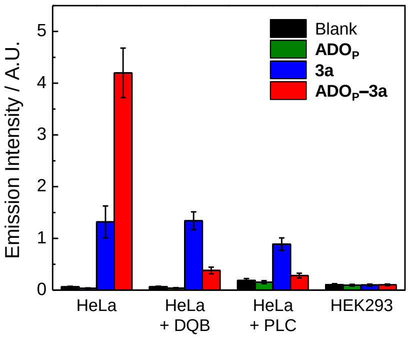

Figure S12. Corrected cell fluorescence of HeLa cells without pretreatment, HeLa cells pretreated with DQB (20 μM, 1 h), HeLa cells pretreated with PLC (0.2 unit mL^{-1}, 1 h), and HEK293 cells, incubated with blank medium (4 h; black), probe **ADOP** (500 μM, 4 h; green), complex **3a** (5 μM, 4 h; blue), or adduct **ADOP–3a** ([**ADOP**] = 500 μM, [**3a**] = 5 μM, 4 h; red), and stained with DCFH-DA (5 μM, 30 min; λ_{ex} = 488 nm, λ_{em} = 510 – 530 nm). Error bars represent standard deviations of three independent replicates.

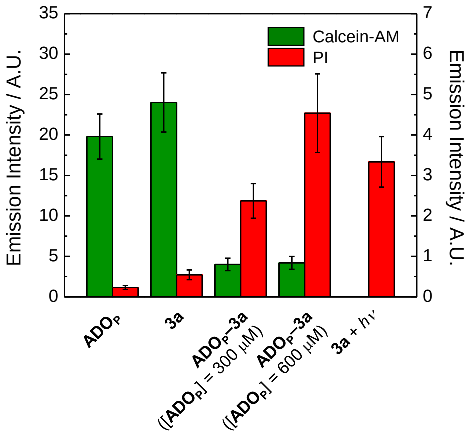

Figure S13. Corrected cell fluorescence of HeLa cells incubated with probe **ADOP** (600 μM, 4 h), complex **3a** (5 μM, 4 h), adduct **ADOP–3a** ([**ADOP**] = 300 or 600 μM, [**3a**] = 5 μM, 4 h), or complex **3a** (5 μM, 4 h) followed by light irradiation (450 nm, 15.5 mW cm^{-2}, 10 min), and stained with Calcein-AM (1 μM, 30 min; λ_{ex} = 488 nm, λ_{em} = 510 – 540 nm; green) and PI (10 μM, 30 min; λ_{ex} = 532 nm, λ_{em} = 610 – 640 nm; red). Error bars represent standard deviations of three independent replicates.

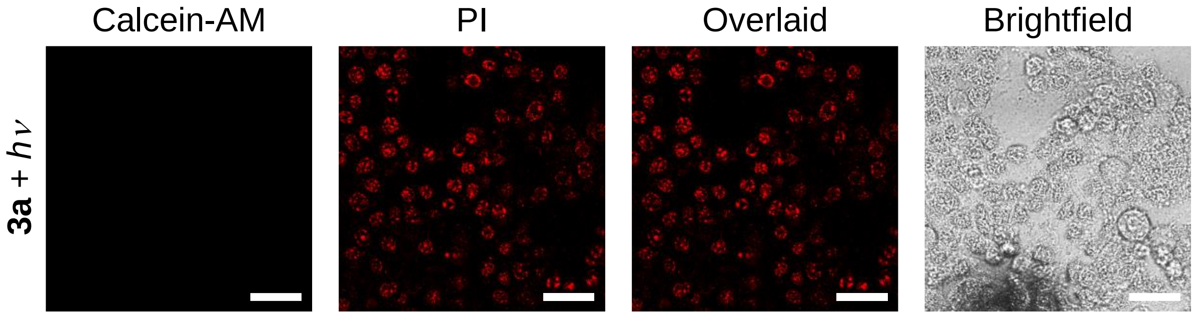

Figure S14. LSCM images of HeLa cells incubated with complex **3a** (5 μM, 4 h) followed by light irradiation (450 nm, 15.5 mW cm^{-2}, 10 min), and stained with Calcein-AM (1 μM, 30 min; λ_{ex} = 488 nm, λ_{em} = 510 – 540 nm) and PI (10 μM, 30 min; λ_{ex} = 532 nm, λ_{em} = 610 – 640 nm). Scale bar = 25 μm.

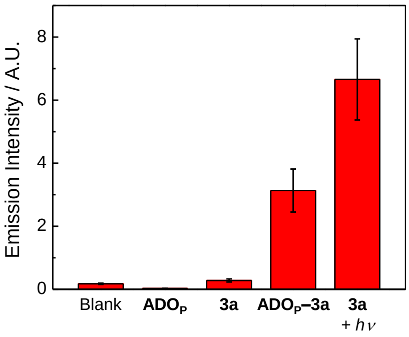

Figure S15. Corrected cell fluorescence of HeLa cells incubated with blank medium (4 h), probe **ADOP** (600 μM, 4 h), complex **3a** (5 μM, 4 h), adduct **ADOP–3a** ([**ADOP**] = 600 μM, [**3a**] = 5 μM, 4 h), or complex **3a** (5 μM, 4 h) followed by light irradiation (450 nm, 15.5 mW cm^{-2}, 10 min), and stained with Alexa Fluor 647–Annexin V conjugate (5 μL, 15 min; λ_{ex} = 633 nm, λ_{em} = 650 – 680 nm). Error bars represent standard deviations of three independent replicates.

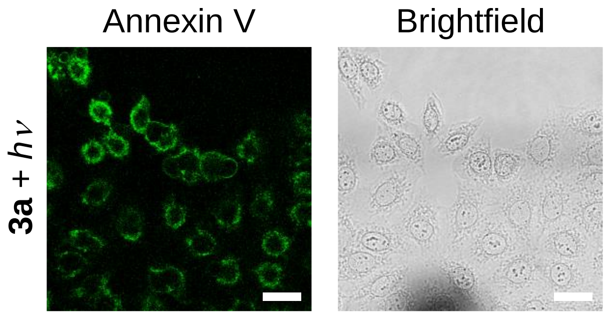

Figure S16. LSCM images of HeLa cells incubated with complex **3a** (5 μM, 4 h) followed by light irradiation (450 nm, 15.5 mW cm^{-2}, 10 min), and stained with Alexa Fluor 647–Annexin V conjugate (5 μL, 15 min; λ_{ex} = 633 nm, λ_{em} = 650 – 680 nm). Scale bar = 25 μm.

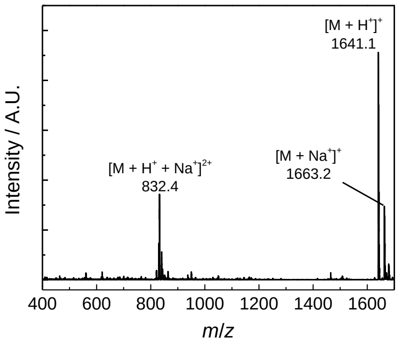

Figure S17. ESI mass spectrum of the ligand bpy-TMCD in MeOH.

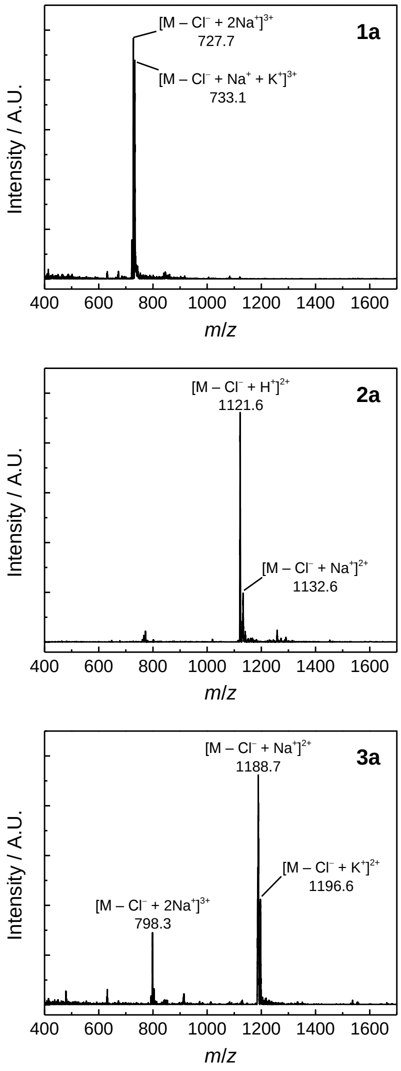

Figure S18. ESI mass spectra of complexes **1a** – **3a** in MeOH.

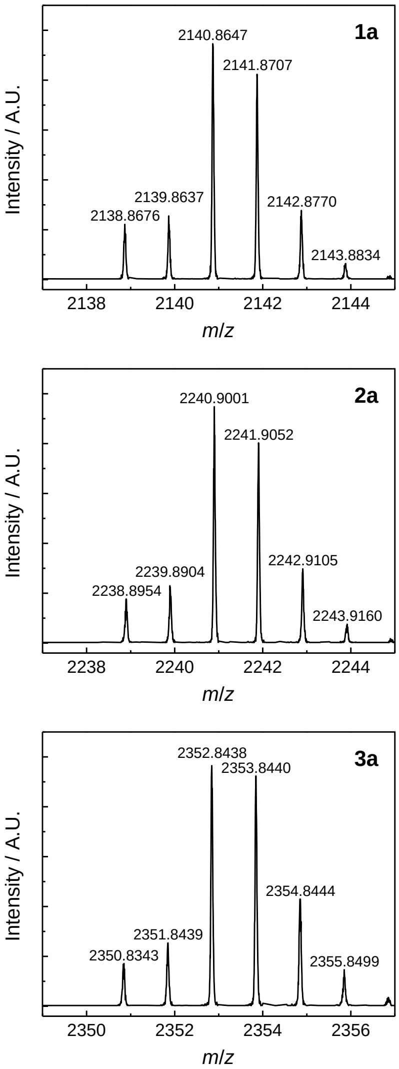

Figure S19. HR-ESI mass spectra of complexes **1a** – **3a** in MeOH.

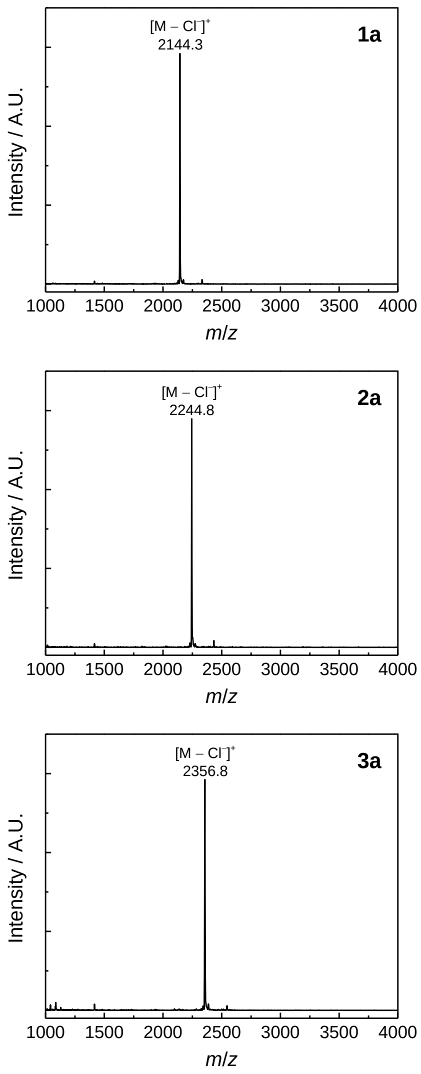

Figure S20. MALDI-TOF mass spectra of complexes **1a** – **3a**.

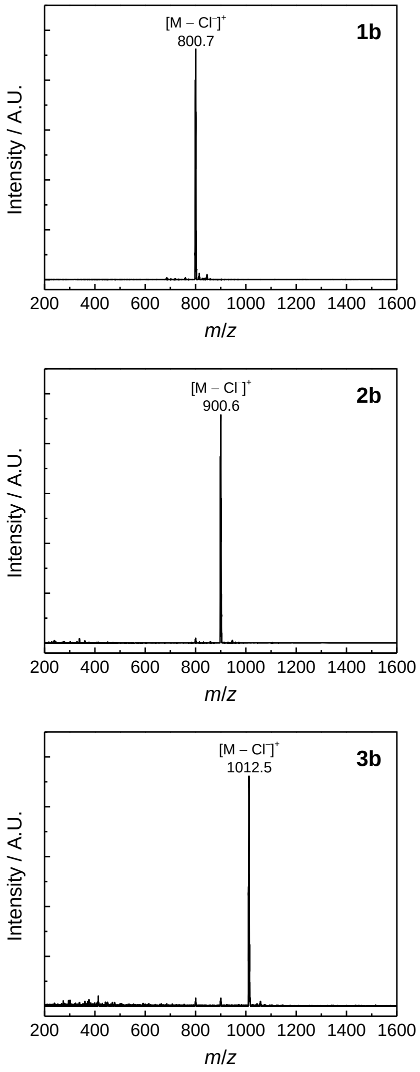

Figure S21. ESI mass spectra of complexes **1b** – **3b** in MeOH.

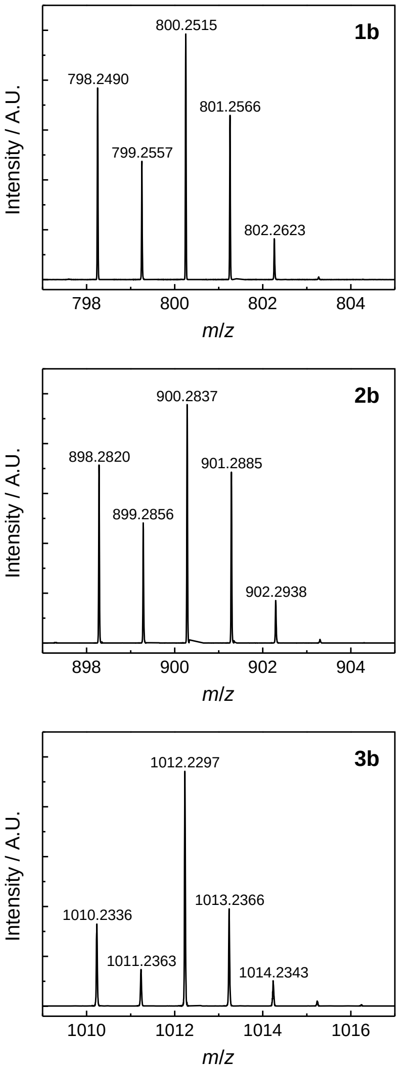

Figure S22. HR-ESI mass spectra of complexes **1b** – **3b** in MeOH.

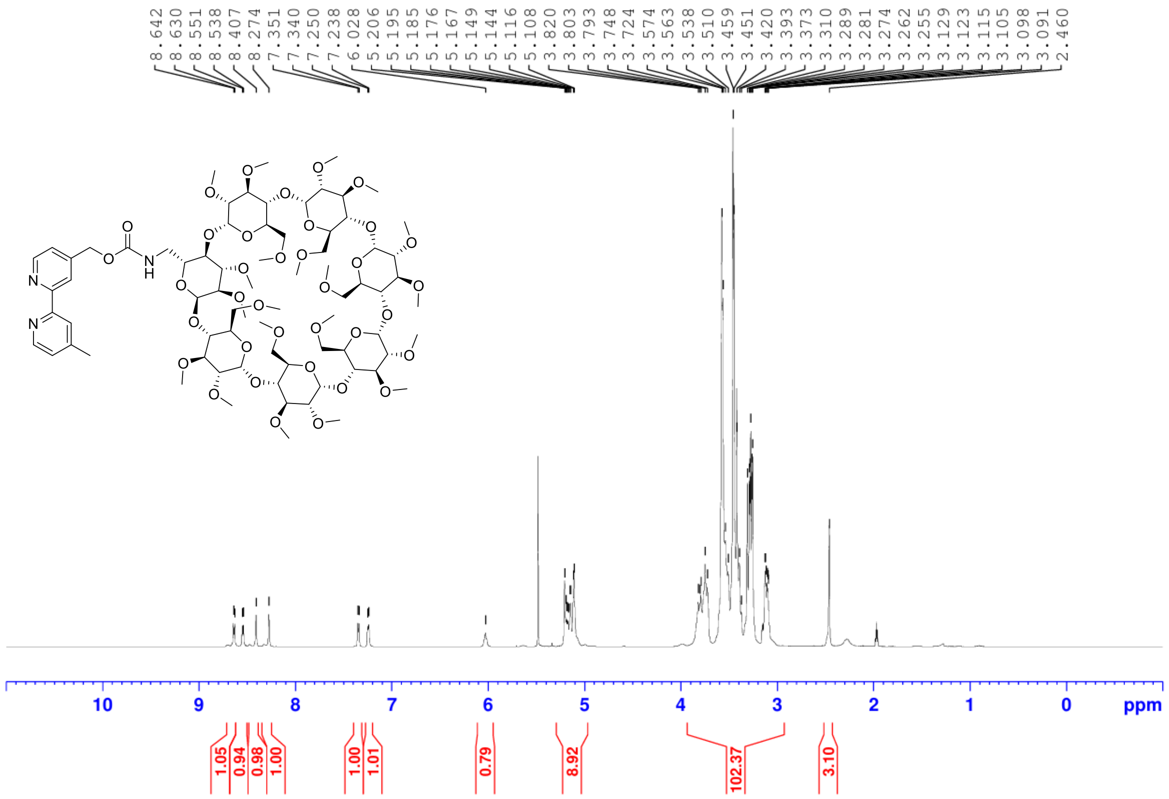

Figure S23. ^{1}H NMR spectrum of the ligand bpy-TMCD in CD_3CN at 298 K.

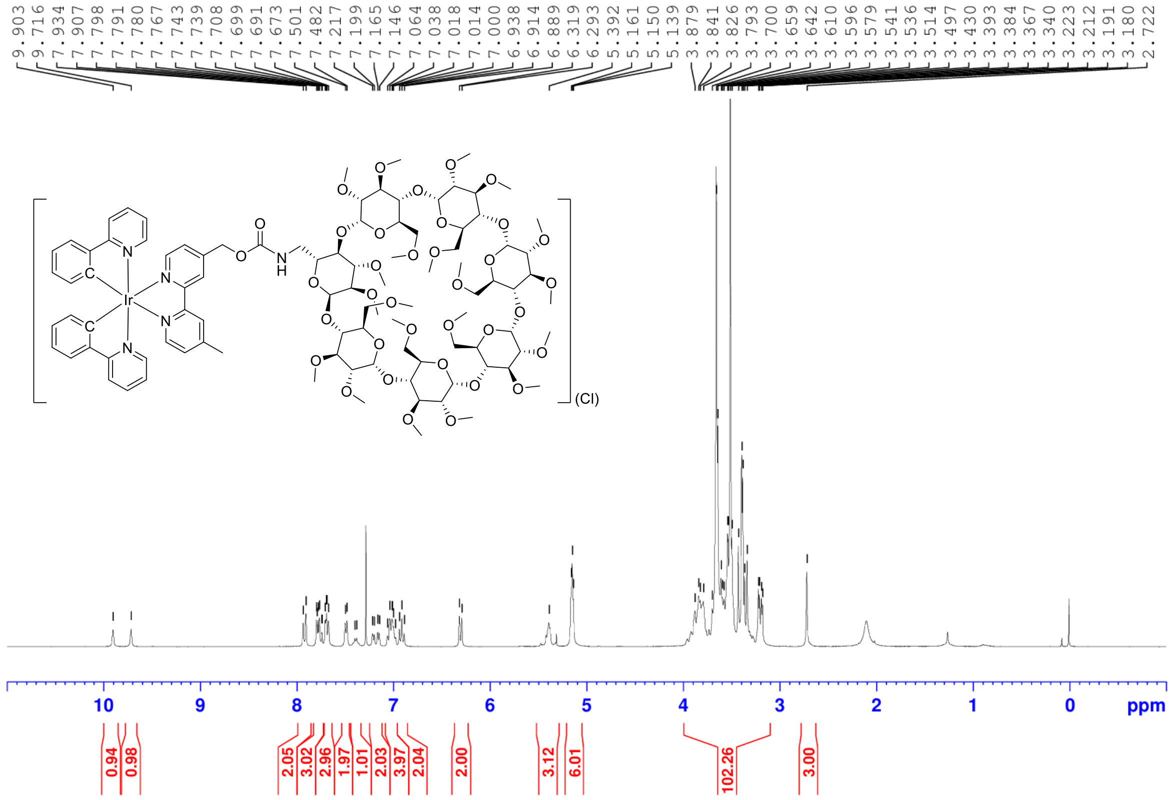

Figure S24. ^{1}H NMR spectrum of complex **1a** in $CDCl_3$ at 298 K.

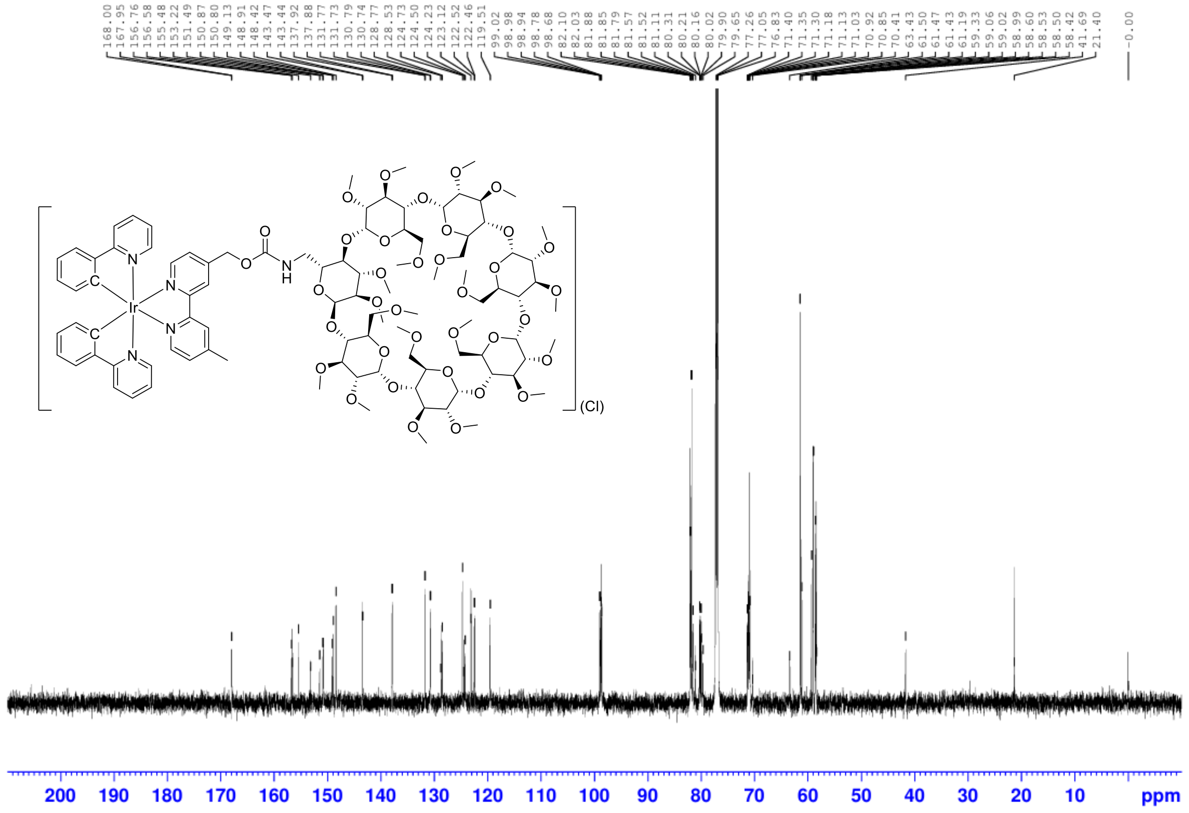

Figure S25. ^{13}C NMR spectrum of complex **1a** in $CDCl_3$ at 298 K.

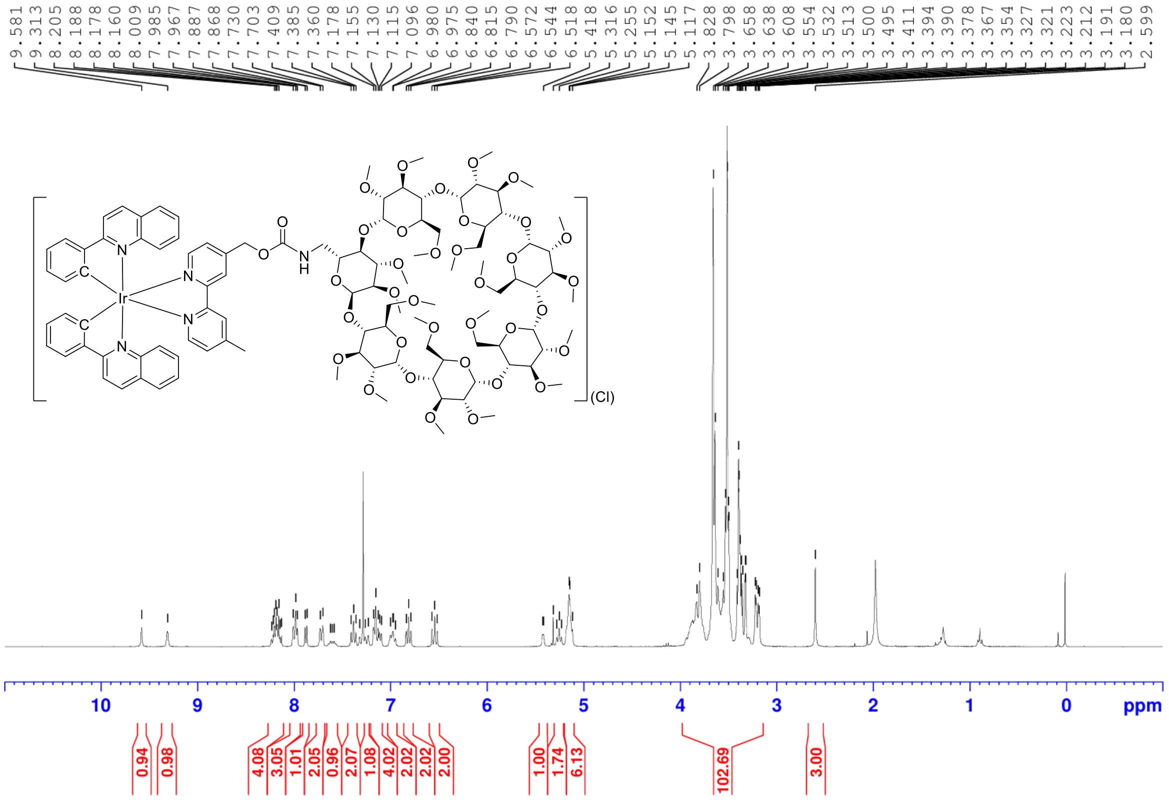

Figure S26. ^{1}H NMR spectrum of complex **2a** in $CDCl_3$ at 298 K.

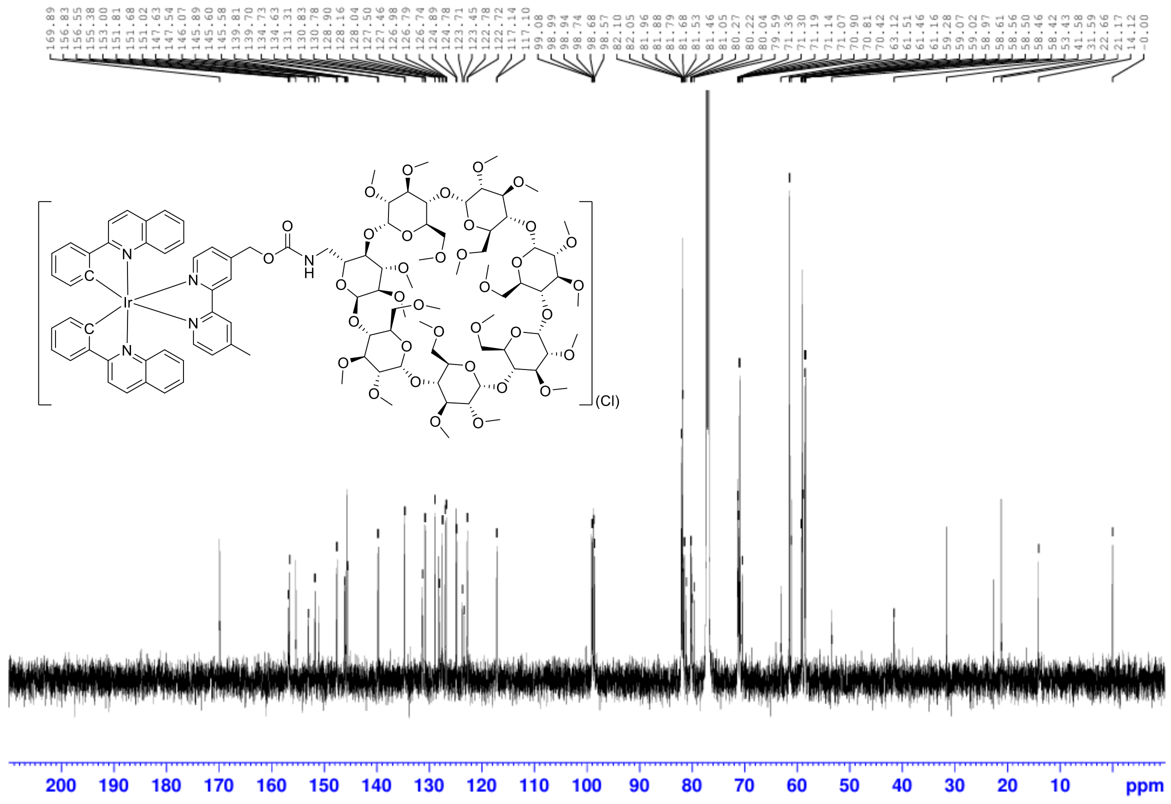

Figure S27. ^{13}C NMR spectrum of complex **2a** in $CDCl_3$ at 298 K.

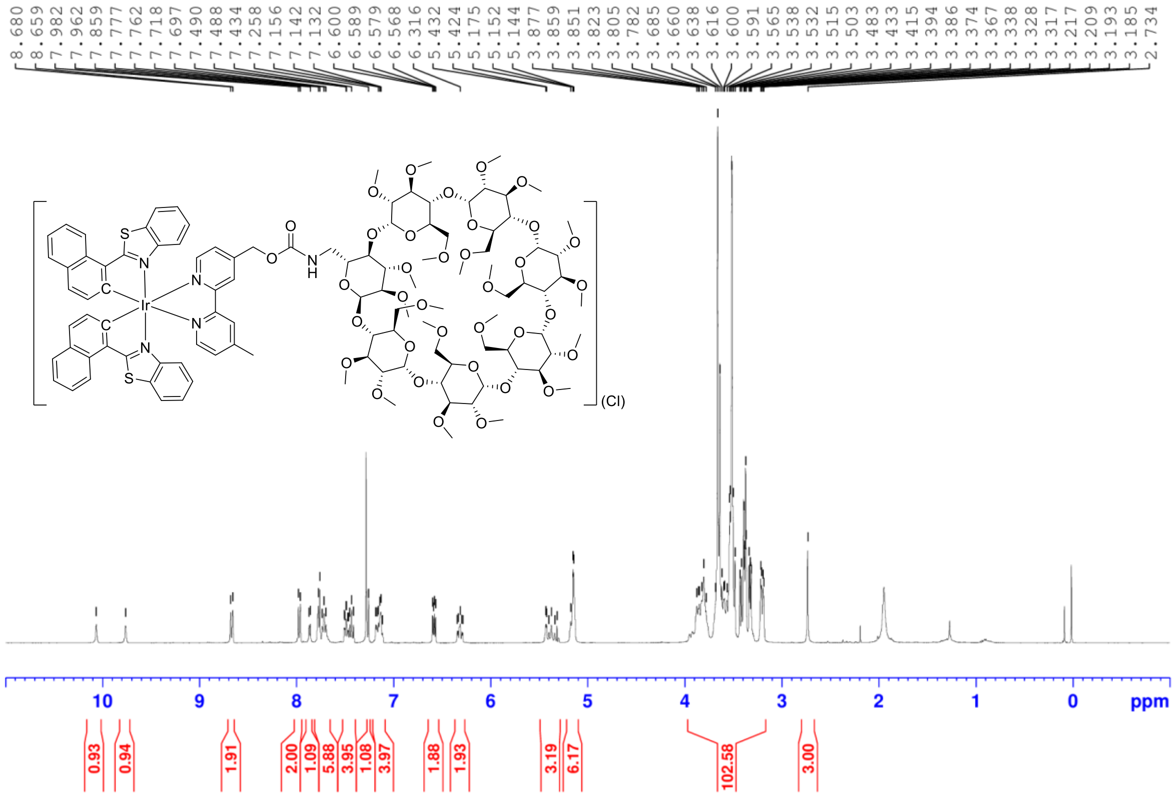

Figure S28. ^{1}H NMR spectrum of complex **3a** in $CDCl_3$ at 298 K.

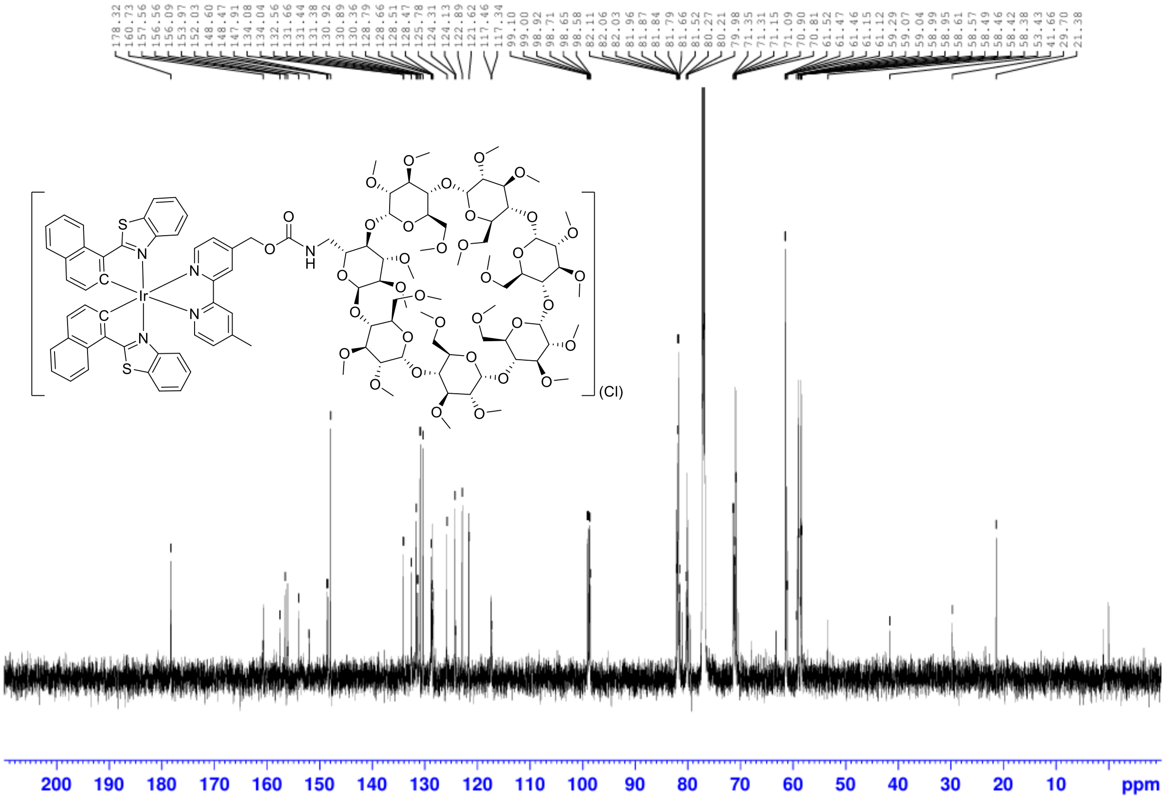

Figure S29. ^{13}C NMR spectrum of complex **3a** in $CDCl_3$ at 298 K.

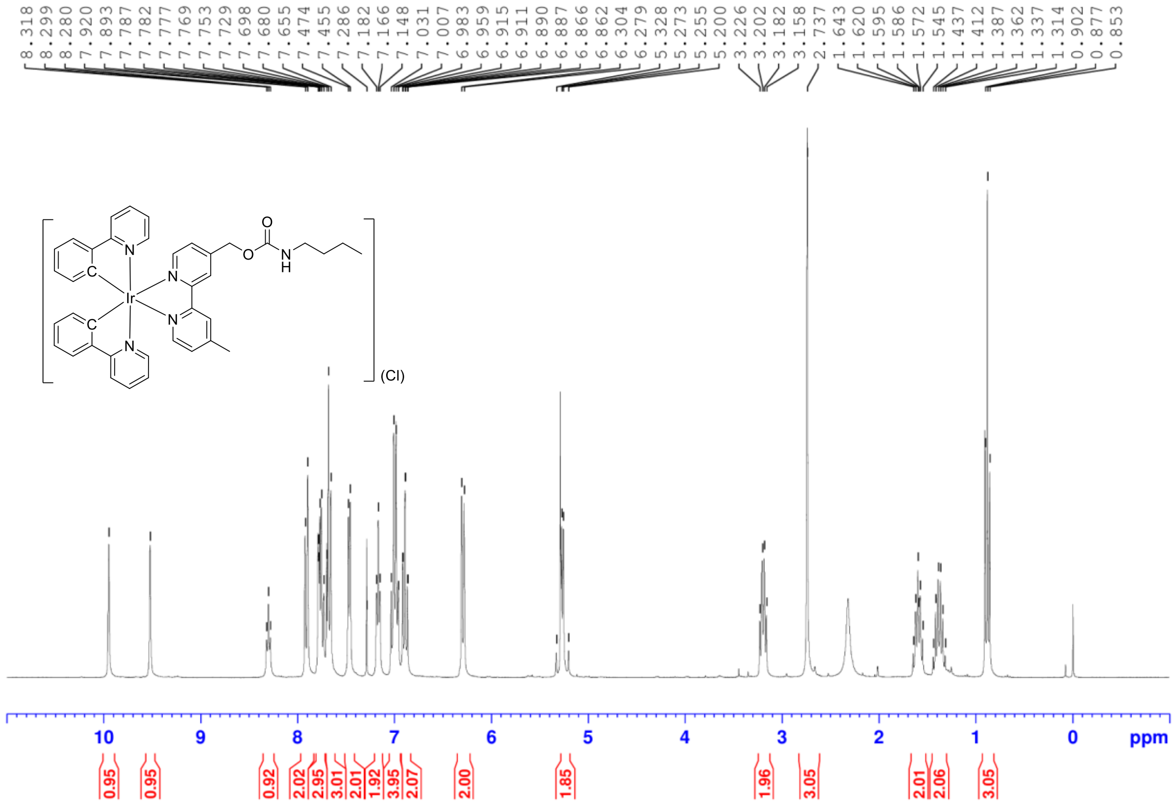

Figure S30. ^{1}H NMR spectrum of complex **1b** in $CDCl_3$ at 298 K.

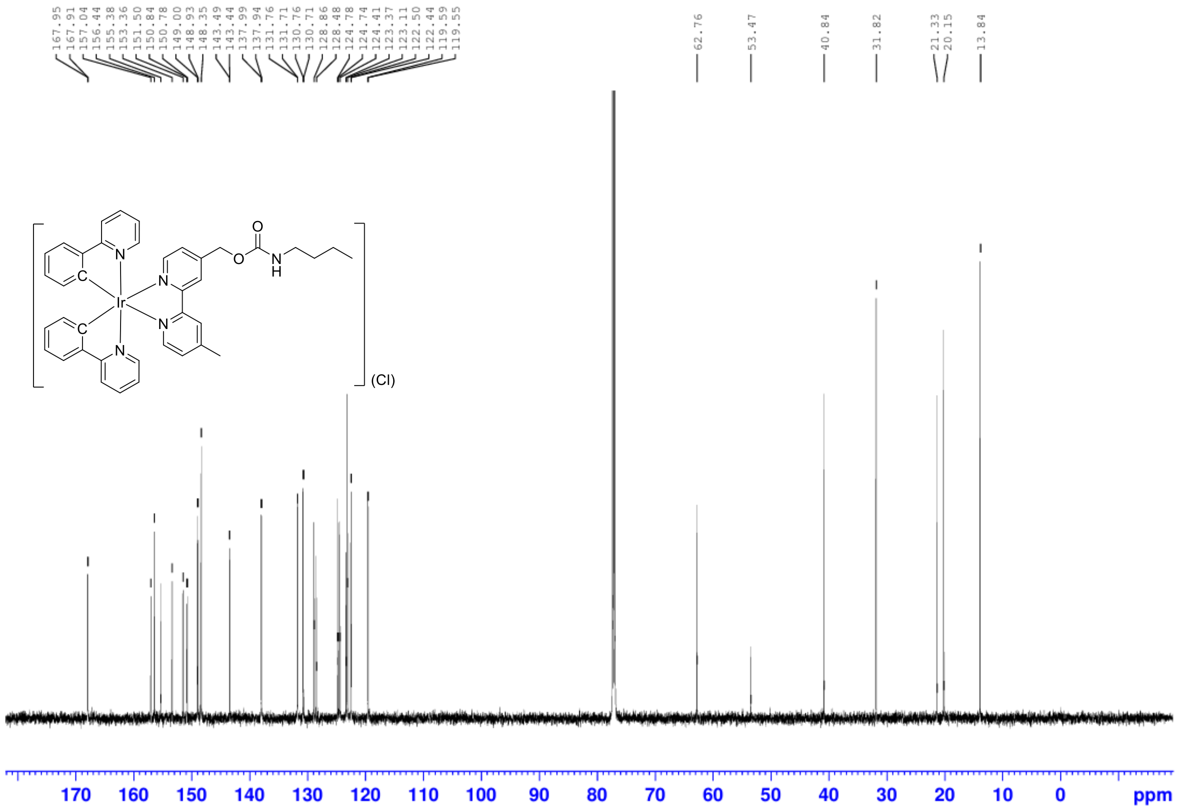

Figure S31. ^{13}C NMR spectrum of complex **1b** in $CDCl_3$ at 298 K.

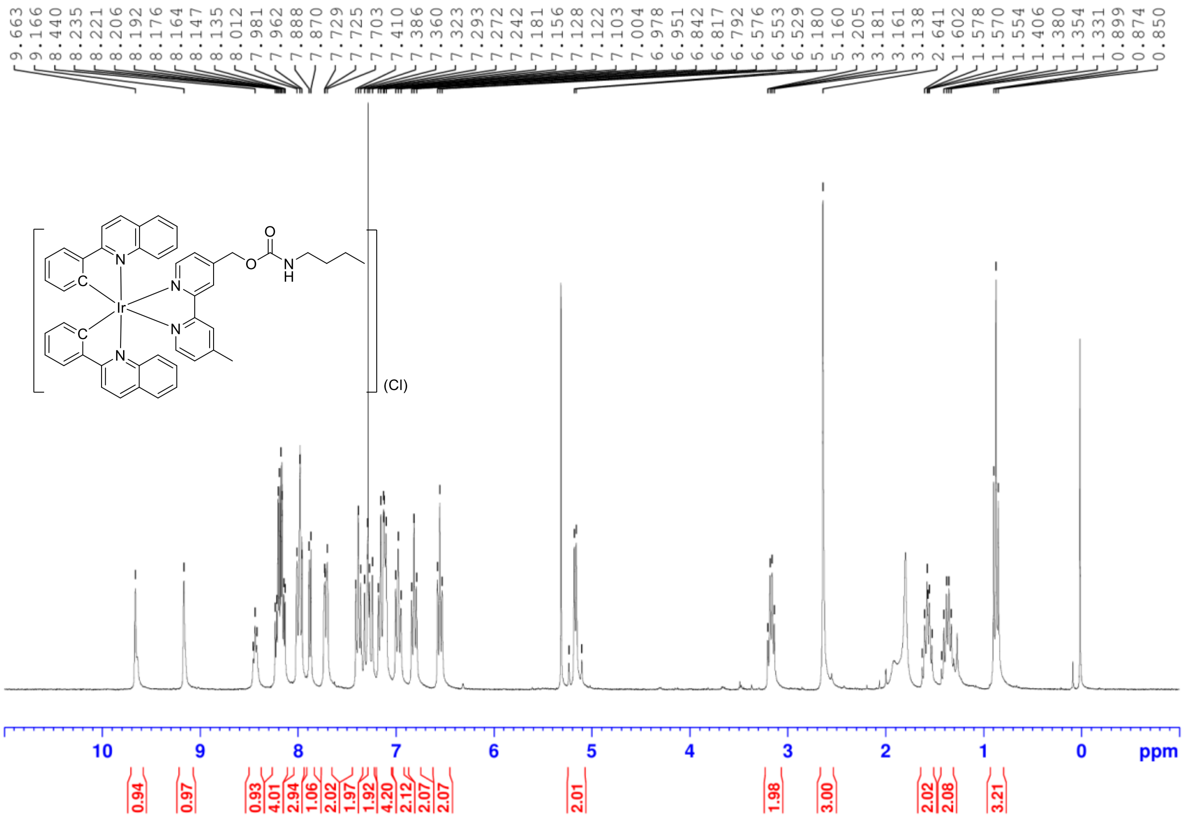

Figure S32. ^{1}H NMR spectrum of complex **2b** in $CDCl_3$ at 298 K.

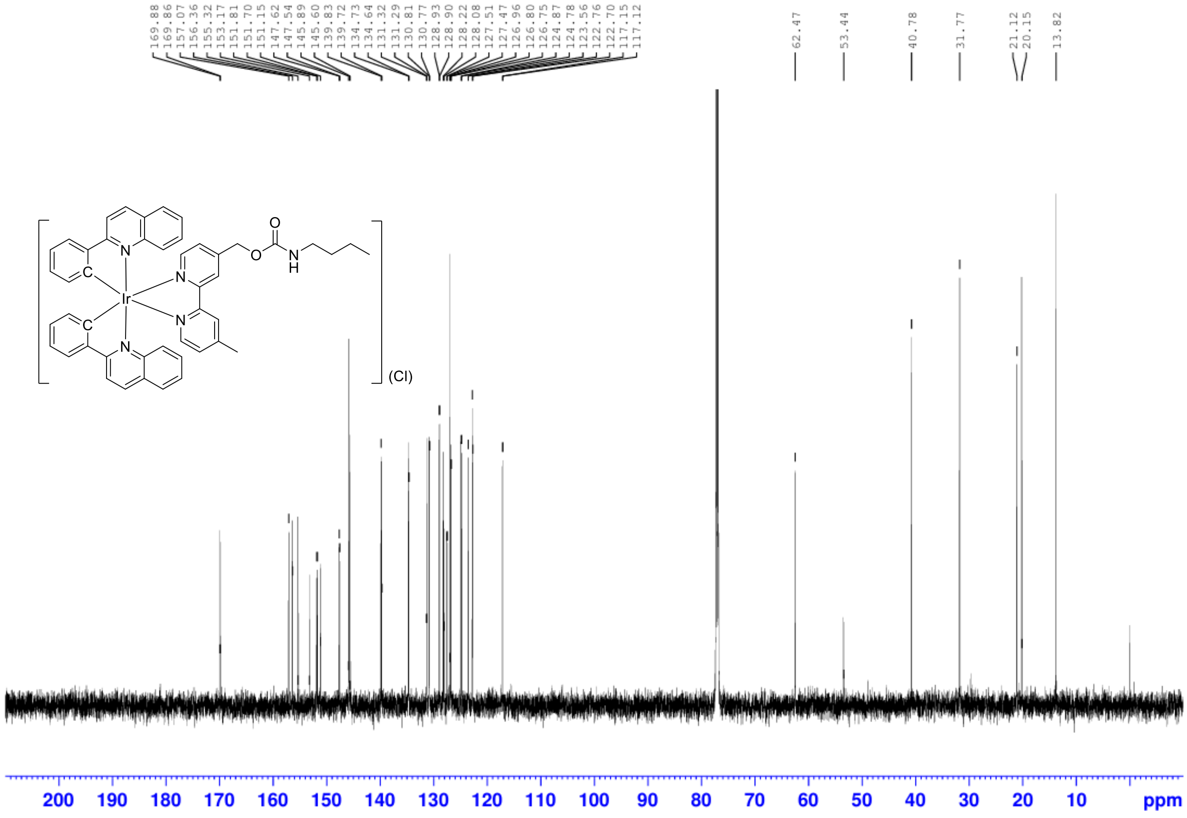

Figure S33. ^{13}C NMR spectrum of complex **2b** in $CDCl_3$ at 298 K.

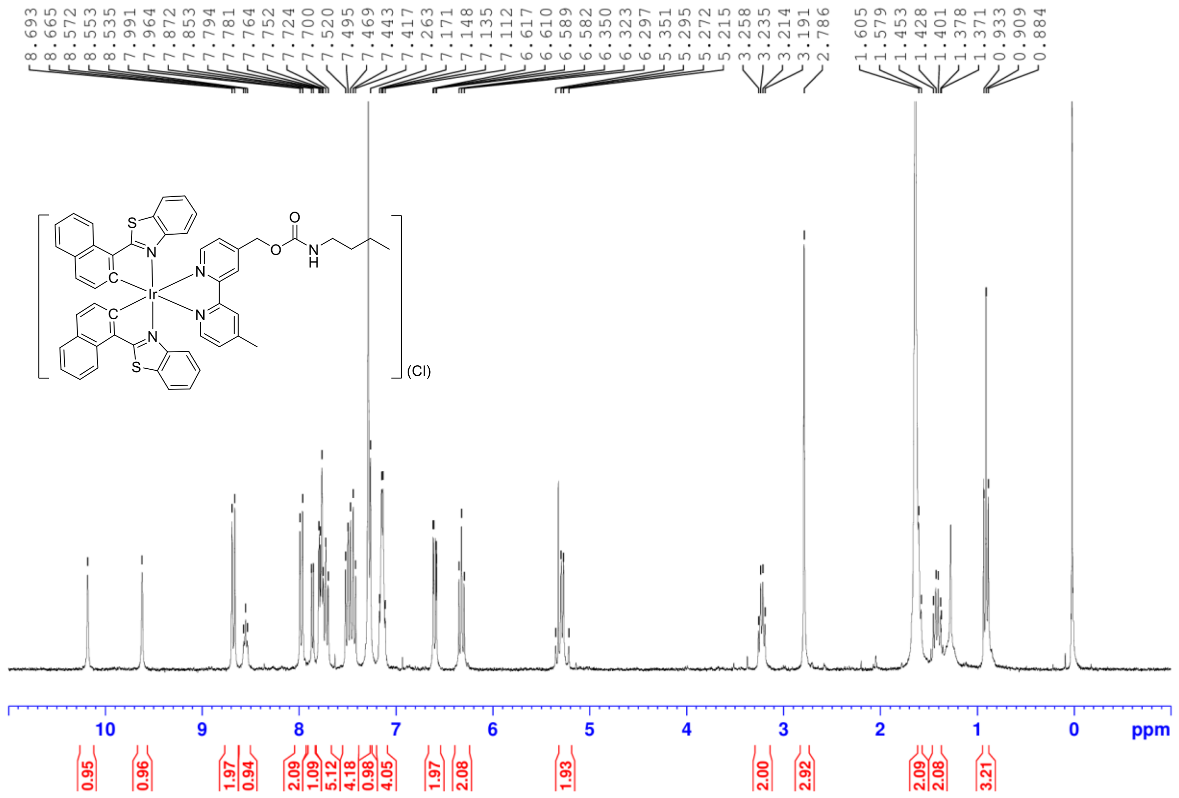

Figure S34. ^{1}H NMR spectrum of complex **3b** in $CDCl_3$ at 298 K.

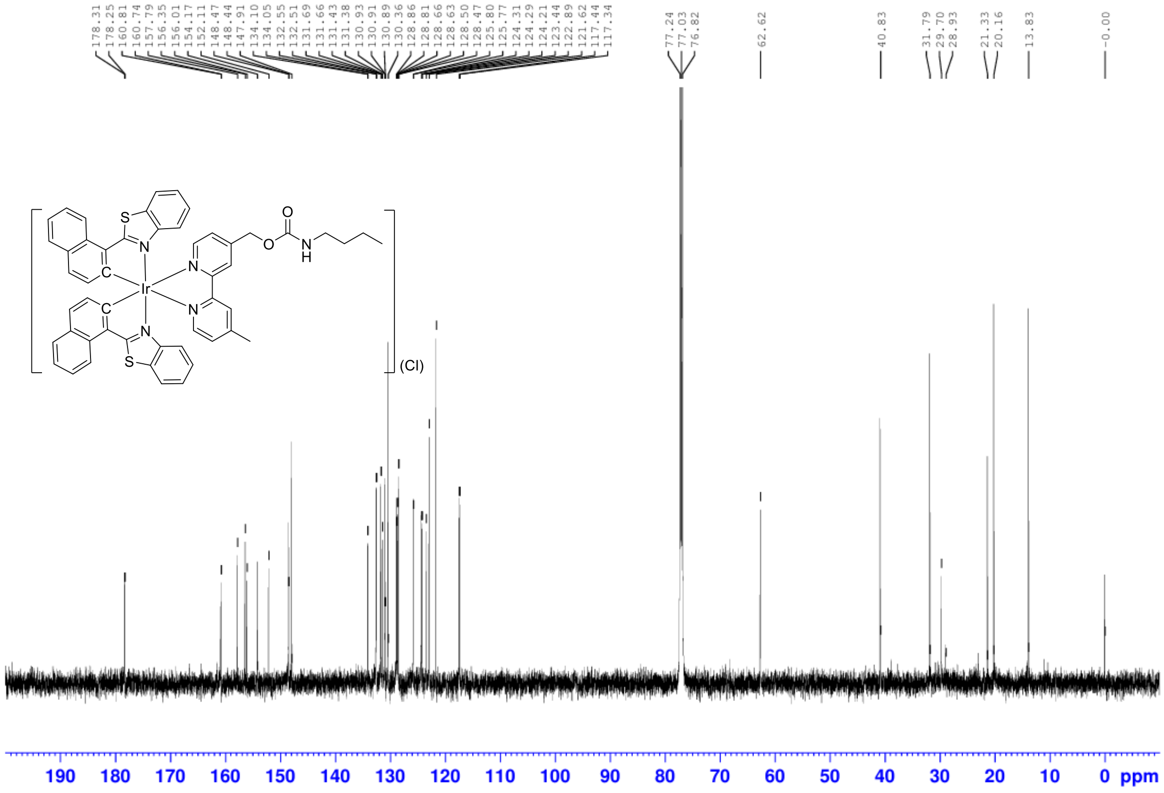

Figure S35. ^{13}C NMR spectrum of complex **3b** in $CDCl_3$ at 298 K.

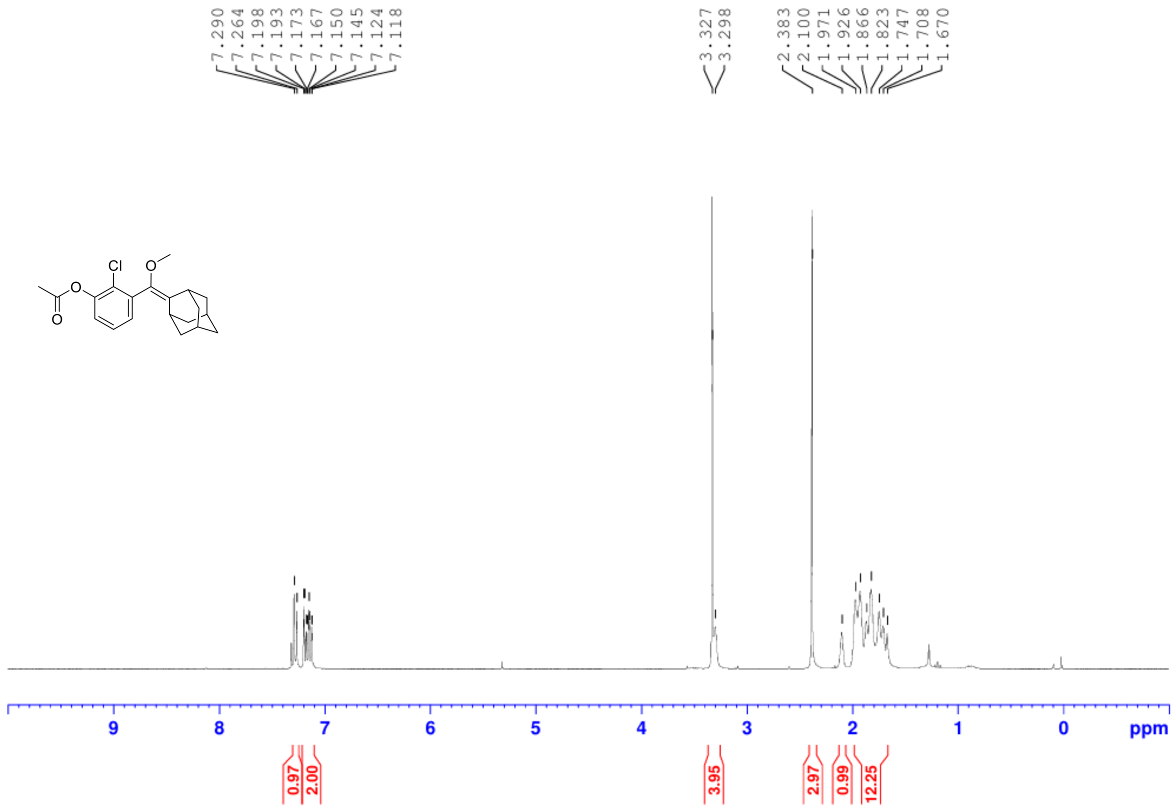

Figure S36. ^{1}H NMR spectrum of the intermediate **ADE$_E$** in CD_3OD at 298 K.

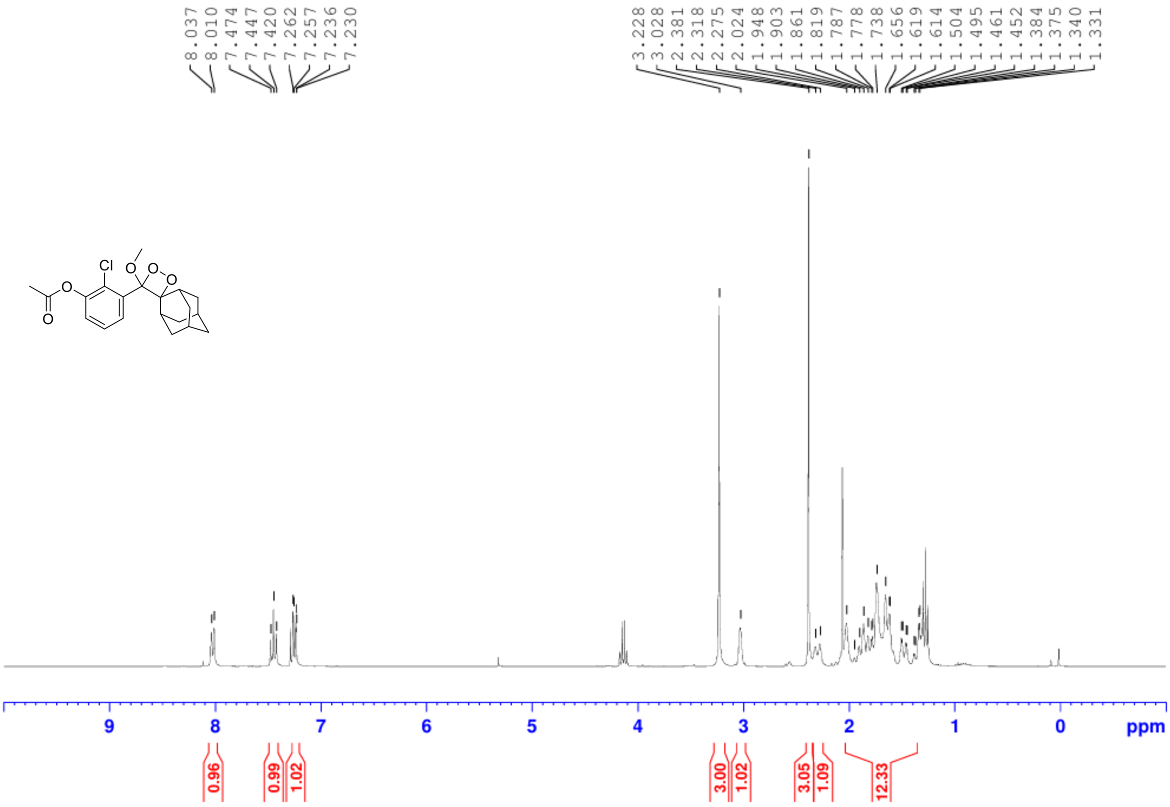

Figure S37. ^{1}H NMR spectrum of probe **ADO$_E$** in CD_3OD at 298 K.

APPENDIX

Synthesis, Characterization, and Properties of [Rh(bsn)$_2$(bpy-TMCD)](Cl) (3c)

[Rh(bsn)$_2$(bpy-TMCD)](Cl) (**3c**)

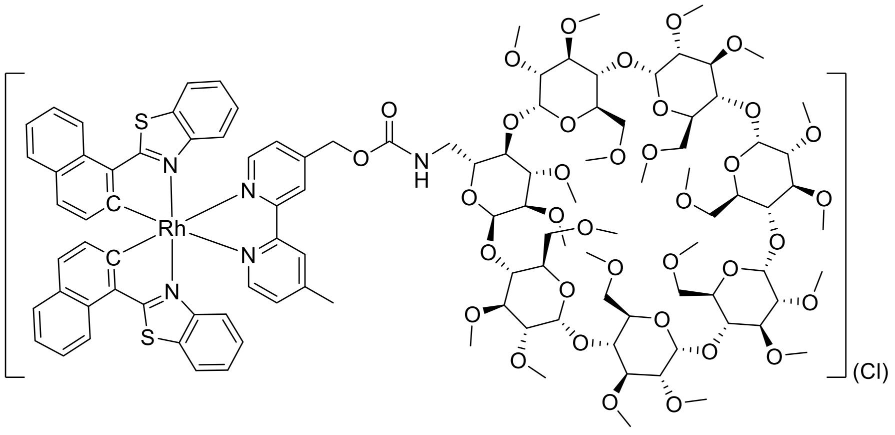

The synthetic procedure was similar to that of complex **1a**, except that [Rh$_2$(bsn)$_4$Cl$_2$] (53 mg, 0.04 mmol) was used instead of [Ir$_2$(ppy)$_4$Cl$_2$]. The solvent was removed under reduced pressure to afford complex **3c** as a yellow solid. Yield: 114 mg (81%). ^{1}H NMR (300 MHz, CDCl$_3$, 298 K, TMS): δ 9.84 (s, 1H, H3 of bpy), 9.61 (s, 1H, H3' of bpy), 8.68 (d, J = 8.6 Hz, 2H, H8 of naphthyl ring of bsn), 7.99 (d, J = 8.0 Hz, 2H, H5 of naphthyl ring of bsn), 7.91 (d, J = 5.7 Hz, 1H, H6 of bpy), 7.86 – 7.70 (m, 5H, H5 of bpy and H4 of benzothiazole ring and H7 of naphthyl ring of bsn), 7.67 – 7.55 (m, 1H, H6' of bpy), 7.54 – 7.35 (m, 5H, H5' of bpy and H5 and H6 of benzothiazole ring of bsn), 7.21 – 7.08 (m, 4H, H4 and H6 of naphthyl ring of bsn), 6.65 – 6.57 (m, 2H, H7 of benzothiazole ring of bsn), 6.47 – 6.35 (m, 2H, H3 of naphthyl ring of bsn), 5.44 – 5.33 (m, 2H, CH$_2$ of bpy), 5.21 – 5.09 (m, 7H, (NHCH$_2$CHO)OC*H*CH and O$_2$C*H*CH of TMCD), 4.01 – 3.13 (m, 102H, OC*H*(CH$_2$)(CH), OC*H*CH$_2$, NHC*H*$_2$, OCH$_2$, and OCH$_3$ of TMCD), 2.68 (s, 3H, CH$_3$ on bpy). ^{13}C NMR (150

MHz, $CDCl_3$, 298 K, TMS): δ 176.29, 176.10, 173.79, 156.59, 156.35, 154.94, 154.04, 152.07, 148.19, 148.07, 147.63, 134.11, 131.53, 131.45, 130.46, 130.25, 128.58, 128.45, 128.29, 128.13, 125.97, 124.81, 123.95, 123.43, 122.72, 121.85, 118.06, 117.95, 99.06, 98.97, 98.91, 98.72, 98.61, 82.04, 81.95, 81.80, 81.57, 81.45, 81.04, 80.19, 79.97, 79.63, 77.25, 77.04, 76.83, 71.35, 71.20, 71.14, 70.91, 70.82, 70.40, 63.33, 61.45, 61.15, 59.24, 59.00, 58.58, 58.44, 53.43, 41.61, 29.70, 21.41. IR (KBr) $\tilde{\nu}$/cm^{-1}: 3413 (N–H), 2928 (C–H), 1617 (C=O), 1038 (C–O–C). MALDI-TOF-MS (CCA) *m/z* found: 2264.6 [M – Cl^-]$^+$ calcd: 2264.4. HR-MS (ESI, positive mode) *m*/*z* found: 2263.8545 [M – Cl^-]$^+$ calcd for $RhC_{109}H_{141}N_5O_{36}S_2$ 2263.7886.

Table A1. Electronic absorption spectral data of complex **3c** at 298 K.

Solvent	λ_{abs}/nm (ε/dm^3 mol^{-1} cm^{-1})
CH_2Cl_2	270 sh (41,235), 307 (22,580), 332 sh (21,460), 344 (25,955), 416 (13,680), 437 (14,605)
CH_3CN	270 sh (39,605), 307 (22,000), 332 sh (21,040), 344 (25,210), 415 (12,760), 433 (13,140)
H_2O	270 sh (35,855), 307 (21,275), 333 sh (18,860), 345 (21,730), 416 (11,790), 438 (11,510)

Table A2. Photophysical data of complex **3c**.

Medium (T/K)	λ_{em}/nm[a]	τ_o/μs[b]	Φ_{em}[c]
CH_2Cl_2 (298)	587 (max), 635, 690 sh	29.49	0.035
CH_3CN (298)	586 (max), 634, 689 sh	19.66	0.026
H_2O (298)	588 (max), 639, 697 sh	52.24	0.025
Glass[d] (77)	580 (max), 598, 631, 649, 690, 713 sh	111.34	

[a] λ_{ex} = 350 nm.

[b] The lifetimes were measured at the emission maxima (λ_{ex} = 355 nm).

[c] $[Ru(bpy)_3]Cl_2$ was used as the reference (Φ_{em} = 0.040 in aerated H_2O, λ_{ex} = 455 nm).[10]

[d] EtOH/MeOH (4:1, *v*/*v*).

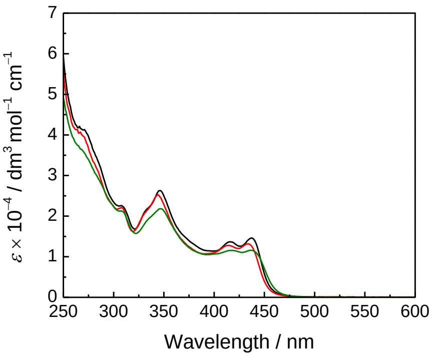

Figure A1. Electronic absorption spectra of complex **3c** in CH_3CN (black), CH_2Cl_2 (red), and H_2O (green) at 298 K.

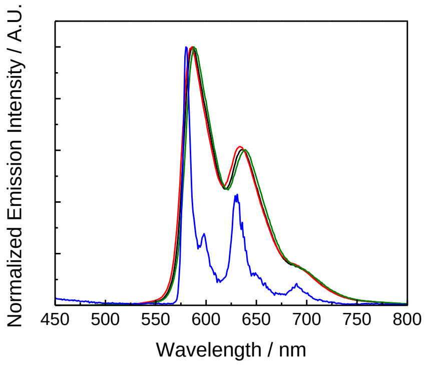

Figure A2. Normalized emission spectra of complex **3c** in CH_2Cl_2 (black), CH_3CN (red), and H_2O (green) at 298 K and alcohol glass at 77 K (blue).

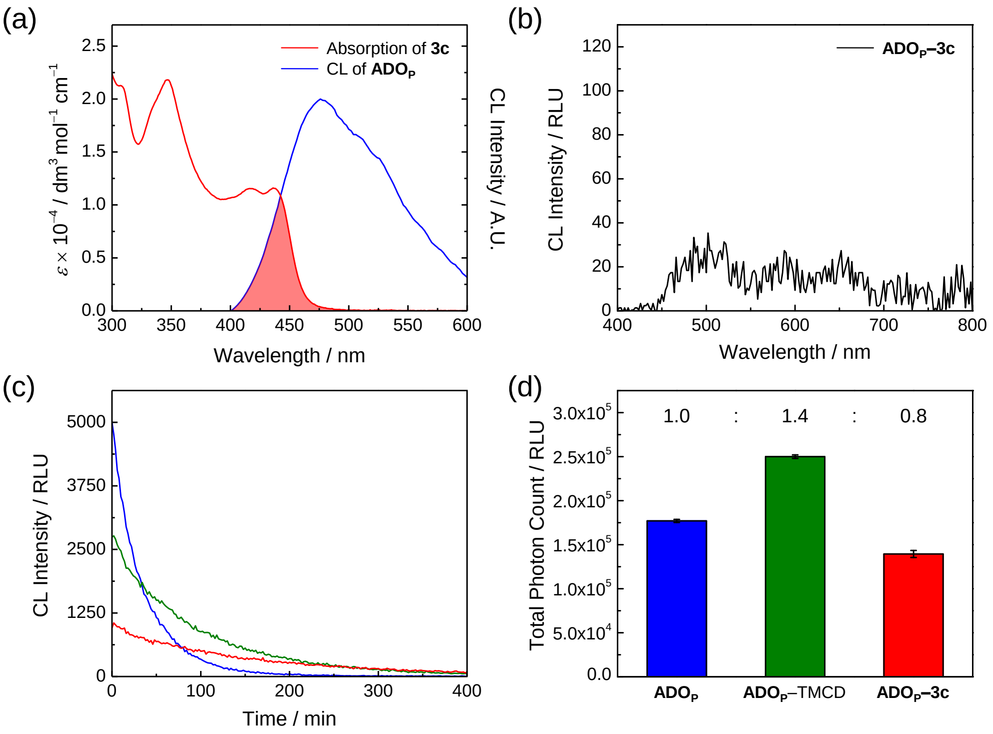

Figure A3. (a) Absorption spectrum of complex **3c** in H_2O (red) overlaid with the CL spectrum of probe **ADO$_P$** (blue) in Tris buffer (50 mM, pH 9.0) at 298 K in the presence of ALP (1 unit mL^{-1}). Shaded region indicates spectral overlap. (b) CL spectrum of adduct **ADO$_P$–3c** ([**ADO$_P$**] = 100 µM, [**3c**] = 100 µM) in Tris buffer (50 mM, pH 9.0) in the presence of ALP (1 unit mL^{-1}). (c) CL kinetic profiles and (d) total photon counts for probe **ADO$_P$** (10 µM) (blue), adduct **ADO$_P$**–TMCD ([**ADO$_P$**] = 10 µM, [TMCD] = 100 µM) (green), and adduct **ADO$_P$–3c** ([**ADO$_P$**] = 10 µM, [**3c**] = 100 µM) (red) in Tris buffer (50 mM, pH 9.0) at 298 K in the presence of ALP (1 unit mL^{-1}).

Discussion

Complex **3c** displayed intense spin-allowed intraligand (^{1}IL) ($\pi \rightarrow \pi^*$) (bpy-TMCD and bsn) absorption bands at *ca*. 270 – 345 nm and weaker spin-allowed metal-to-ligand charge-transfer (^{1}MLCT) (dπ(Rh) $\rightarrow$ π^*(bsn)) transitions at *ca*. 415 – 450 nm (Table A1 and Figure A1).[14–16] Upon photoexcitation, it exhibited a vibronically structured and long-lived emission band (Table A2 and Figure A2) in both fluid solutions at 298 K and in low-temperature alcohol glass at 77 K, indicative of an excited state with predominant ^{3}IL ($\pi \rightarrow \pi^*$) (bsn) character.[14–16] However, it showed very weak emission (Φ_{em} = 0.025 – 0.035; Table A2), attributed to the presence of a thermally accessible nonemissive metal-centered state. Despite large overlap between the CL spectrum of probe **ADO_P** and the absorption spectrum of complex **3c** (J = 7.59 × 10^{15} M^{-1} cm^{-1} nm^4; Figure A3a), the CL response of their host–guest adduct **ADO$_P$–3c** was very weak upon ALP activation (Figure A3b). Its total light emission was even lower than that of adduct **ADO$_P$**–TMCD (Figure A3c and d) under the same experimental conditions. The very weak CL signal implies that CRET occurred from probe **ADO$_P$** to complex **3c**. However, due to its very low emission quantum yields (Table A2), the rhodium(III) complex functioned more as a CL quencher rather than a CL enhancer.

(a)

(b)

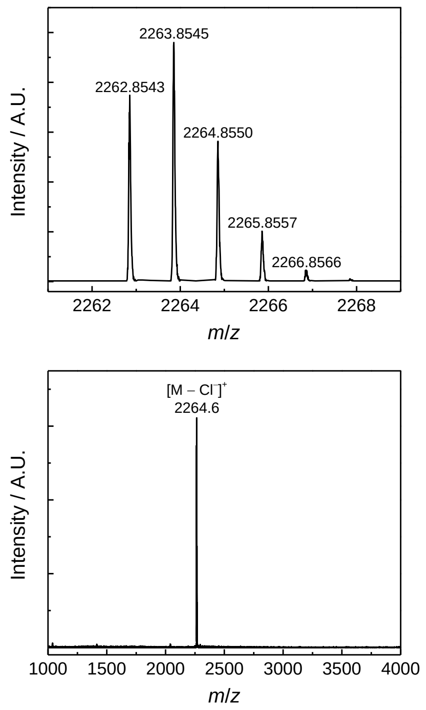

Figure A4. (a) HR-ESI and (b) MALDI-TOF mass spectra of complex **3c**.

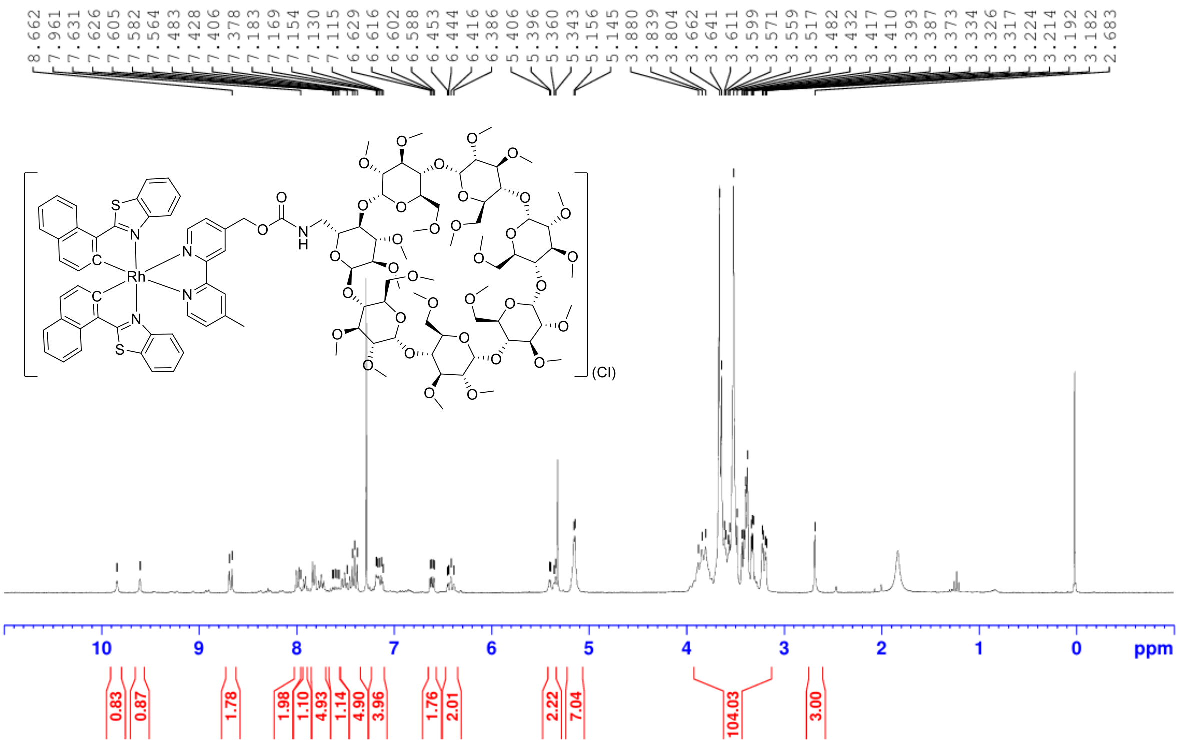

Figure A5. ^{1}H NMR spectrum of complex **3c** in $CDCl_3$ at 298 K.

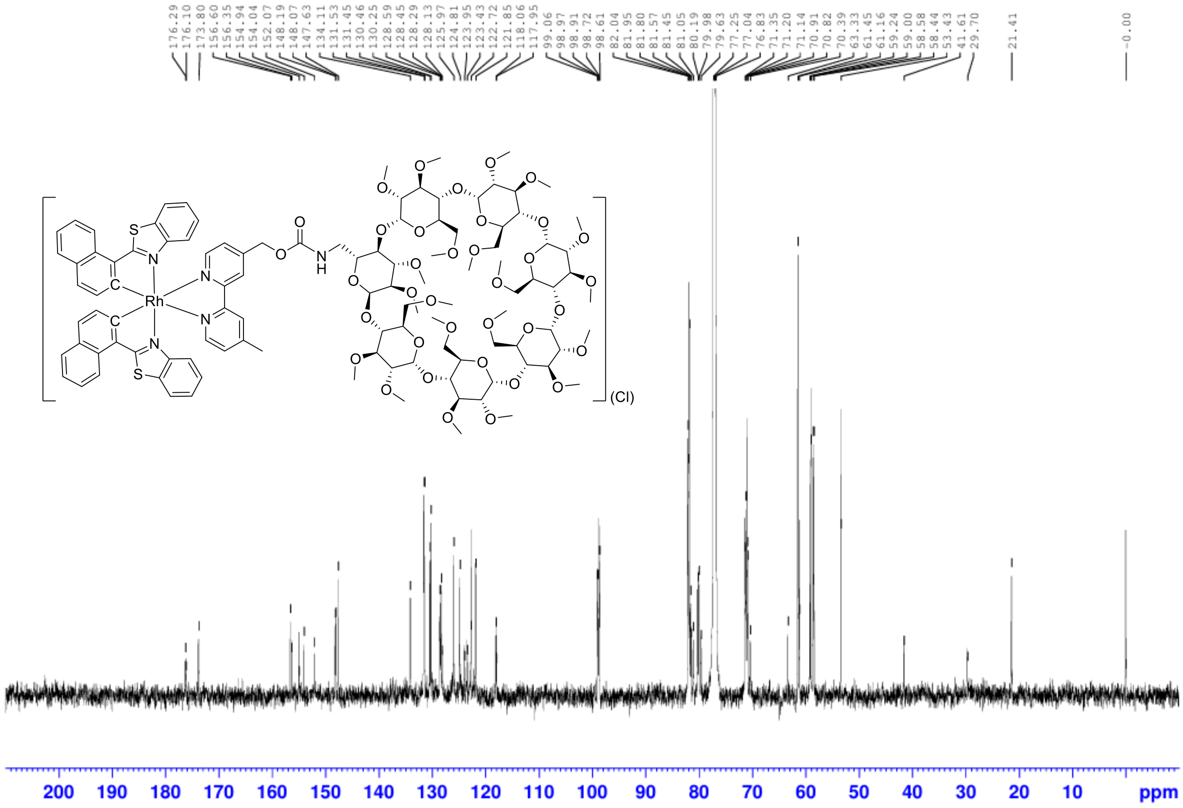

Figure A6. ^{13}C NMR spectrum of complex **3c** in $CDCl_3$ at 298 K.

REFERENCES

1. Perrin, D. D.; Armarego, W. L. F. *Purification of Laboratory Chemicals*, 3rd ed.; Pergamon Press: New York, 1988.
2. Lo, K. K.-W.; Li, C.-K.; Lau, J. S.-Y. Luminescent Cyclometalated Iridium(III) Arylbenzothiazole Biotin Complexes. *Organometallics* **2005**, *24*, 4594–4601.
3. Lo, K. K.-W.; Chung, C.-K.; Lee, T. K.-M.; Lui, L.-H.; Tsang, K. H.-K.; Zhu, N. New Luminescent Cyclometalated Iridium(III) Diimine Complexes as Biological Labeling Reagents. *Inorg. Chem.* **2003**, *42*, 6886–6897.
4. Tang, T. S.-M.; Yip, A. M.-H.; Zhang, K. Y.; Liu, H.-W.; Wu, P. L.; Li, K. F.; Cheah, K. W.; Lo, K. K.-W. Bioorthogonal Labeling, Bioimaging, and Photocytotoxicity Studies of Phosphorescent Ruthenium(II) Polypyridine Dibenzocyclooctyne Complexes. *Chem. Eur. J.* **2015**, *21*, 10729–10740.
5. Gnaim, S.; Scomparin, A.; Eldar-Boock, A.; Bauer, C. R.; Satchi-Fainaro, R.; Shabat, D. Light Emission Enhancement by Supramolecular Complexation of Chemiluminescence Probes Designed for Bioimaging. *Chem. Sci.* **2019**, *10*, 2945–2955.
6. Lee, L. C.-C.; Tsang, A. W.-Y.; Liu, H.-W.; Lo, K. K.-W. Photofunctional Cyclometalated Iridium(III) Polypyridine Complexes Bearing a Perfluorobiphenyl Moiety for Bioconjugation, Bioimaging, and Phototherapeutic Applications. *Inorg. Chem.* **2020**, *59*, 14796–14806.

7. Sprouse, S.; King, K. A.; Spellane, P. J.; Watts, R. J. Photophysical Effects of Metal–Carbon σ Bonds in Ortho-Metalated Complexes of Ir(III) and Rh(III). *J. Am. Chem. Soc.* **1984**, *106*, 6647–6653.

8. Hananya, N.; Boock, A. E.; Bauer, C. R.; Satchi-Fainaro, R.; Shabat, D. Remarkable Enhancement of Chemiluminescent Signal by Dioxetane–Fluorophore Conjugates: Turn-ON Chemiluminescence Probes with Color Modulation for Sensing and Imaging. *J. Am. Chem. Soc.* **2016**, *138*, 13438–13446.

9. Demas, J. N.; Crosby, G. A. The Measurement of Photoluminescence Quantum Yields. A Review. *J. Phys. Chem.* **1971**, *75*, 991–1024.

10. Suzuki, K.; Kobayashi, A.; Kaneko, S.; Takehira, K.; Yoshihara, T.; Ishida, H.; Shiina, Y.; Oishi, S.; Tobita, S. Reevaluation of Absolute Luminescence Quantum Yields of Standard Solutions Using a Spectrometer with an Integrating Sphere and a Back-Thinned CCD Detector. *Phys. Chem. Chem. Phys.* **2009**, *11*, 9850–9860.

11. Abdel-Shafi, A. A.; Beer, P. D.; Mortimer, R. J.; Wilkinson, F. Photosensitized Generation of Singlet Oxygen from Vinyl Linked Benzo-Crown-Ether–Bipyridyl Ruthenium(II) Complexes. *J. Phys. Chem. A* **2000**, *104*, 192–202.

12. Li, Z.; Wang, Y.; Zhang, G.; Xu, W.; Han, Y. Chemiluminescence Resonance Energy Transfer in the Luminol–CdTe Quantum Dots Conjugates. *J. Lumin.* **2010**, *130*, 995–999.

13. Lee, J.; Seliger, H. H. Absolute Spectral Sensitivity of Phototubes and the Application to the Measurement of the Absolute Quantum Yields of Chemiluminescence and Bioluminescence. *Photochem. Photobiol.* **1965**, *4*, 1015–1048.

14. Lo, K. K.-W.; Li, C.-K.; Lau, K.-W.; Zhu, N. Luminescent Cyclometalated Rhodium(III) Bis(pyridylbenzaldehyde) Complexes with Long-lived Excited States. *Dalton Trans.* **2003**, 4682–4689.

15. Wei, F.; Lai, S.-L.; Zhao, S.; Ng, M.; Chan, M.-Y.; Yam, V. W.-W.; Wong, K. M.-C. Ligand Mediated Luminescence Enhancement in Cyclometalated Rhodium(III) Complexes and Their Applications in Efficient Organic Light-Emitting Devices. *J. Am. Chem. Soc.* **2019**, *141*, 12863–12871.

16. Jiang, K. G.-M.; Wei, F.; Leung, P. K.-K.; Wu, S.; Lo, K. K.-W.; Wong, K. M.-C. Rhodamine-Functionalised Rhodium(III) Complexes: Dual Role as Bioimaging Agents and Controllable Reactive Oxygen Species Photosensitisers for Photocytotoxicity Applications. *Chem. Sci.* **2025**, *16*, 22127–22135.
